# Supplementary material for: Interventions supporting the translation of gerontological evidence into practice to optimize functional outcomes for hospitalized older adults: A scoping review
Source: PLoS One. 2025 Jun 16;20(6):e0324953. doi: 10.1371/journal.pone.0324953 (PMC12169582; doi:10.1371/journal.pone.0324953)
Supplement: S2 Table — (DOCX) [file pone.0324953.s002.docx]

**S-2 Table.**

*Inclusion and Exclusion Criteria*

| Inclusion Criteria | Exclusion Criteria |
| --- | --- |
| - Any design (quantitative, qualitative, mixed methods) with a focus on initiatives or care delivery models to improve functional outcomes for persons, 65 years of age and older, in hospital - Address multiple interventions (e.g. maintenance of more than one basic activity of daily living) OR a single focused intervention and outcome (e.g. maintaining mobility or continence). - Includes educational component for health care providers as part of the practice change - Report of contextual factors (interventions) that were addressed in addition to education - Outcome measures that include (patient) functional outcomes | Fails to satisfy any/all inclusion criteria. |
