# Supplementary material for: Interventions supporting the translation of gerontological evidence into practice to optimize functional outcomes for hospitalized older adults: A scoping review
Source: PLoS One. 2025 Jun 16;20(6):e0324953. doi: 10.1371/journal.pone.0324953 (PMC12169582; doi:10.1371/journal.pone.0324953)
Supplement: S3 Table — (DOCX) [file pone.0324953.s003.docx]

**S-3 Table.**

*Search Strategies for MEDLINE*

| Line # | Search Terms |
| --- | --- |
| 1 | Geriatrics/ or exp Aged/ or Health Services for the Aged/ or (elders or elderly or geriatric* or "old age?" or (seniors not "high school") or "older adult*" or centenarian* or nonagenarian* or octogenarian* or septuagenarian* or sexagenarian* or dottering or decrepit or tottering or overaged or "oldest old").mp. |
| 2 | Hospitalization/ |
| 3 | hospital units/ or hospitals/ |
| 4 | ((acute adj1 care) or hospital? or "hospital unit?" or hospitali?ation*).mp. |
| 5 | 2 or 3 or 4 |
| 6 | 1 and 5 |
| 7 | "Activities of Daily Living"/ |
| 8 | (ADL or BADL).ti,kw. |
| 9 | ("activit* of daily living" or "daily life activit*").mp. |
| 10 | Self Care/ |
| 11 | ("self care" or selfcare or "personal care").mp. |
| 12 | ("functional status" or "functional decline").ti,ab,kw. |
| 13 | "functional* independen*".ti,ab,kw. |
| 14 | ("functional mobility" or transferring).ti,ab,kw. |
| 15 | walking/ or dependent ambulation/ |
| 16 | ((walk* or mobility or mobili?ation) adj (assisted or unassisted or aided or unaided or independent* or dependent* or abilit* or function*)).ti,ab,kw. |
| 17 | (ambulation adj1 (assisted or unassisted or aided or unaided or independent* or dependent* or abilit* or function*)).ti,ab,kw. |
| 18 | Hygiene/ or oral hygiene/ |
| 19 | ("personal hygiene" or "personal grooming" or (hair adj (brush* or comb* or styl*)) or toothbrush* or "tooth brush*" or floss* or "oral care").ti,ab,kw. |
| 20 | ((bath* or shower* or clean*) adj (assisted or unassisted or aided or unaided or independent* or dependent*)).ti,ab,kw. |
| 21 | (dress* adj1 (assisted or unassisted or aided or unaided or independent* or dependent*)).ti,ab,kw. |
| 22 | ((toilet* adj (hygiene or cleanliness or behaviour?r or routine*)) or toileting or continence).ti,ab,kw. |
| 23 | (defecation/ or urination/) and (hygiene or hygienic or clean* or behaviour?r or routine*).mp. |
| 24 | ("bladder control" or "bowel? control").ti,ab,kw. |
| 25 | eating/ or drinking/ |
| 26 | (((eat* or drink*) adj1 (assisted or unassisted or aided or unaided or independent* or dependent*)) or "self feed*").ti,ab,kw. |
| 27 | or/7-26 |
| 28 | 6 and 27 |
| 29 | (intervention? or program* or initiative? or strateg*).mp. |
| 30 | 28 and 29 |
| 31 | case reports.pt. |
| 32 | comment.pt. |
| 33 | editorial.pt. |
| 34 | 31 or 32 or 33 |
| 35 | 30 not 34 |
| 36 | (qualitative* or quantitative* or "mixed-method*").ti,ab,kf. |
| 37 | (((systematic or scoping or synthesis or umbrella or integrative or rapid or comprehensive or meta or realist or concept or evidence or narrative or literature) adj3 (review? or anayls?s or synthes?s or ethnography or study or studies or map? or mapping)) or meta-analys?s).ti,kf,pt. |
| 38 | meta-analysis/ or "systematic review"/ |
| 39 | ("health technolog* assessment?" or HTA?).ti,kf,pt. |
| 40 | ("randomi?ed control* trial?" or RCT).ti,kf,pt. |
| 41 | "clinical trial?".ti,kf,pt. |
| 42 | 36 or 37 or 38 or 39 or 40 or 41 |
| 43 | 35 and 42 |
