# Supplementary material for: Interventions supporting the translation of gerontological evidence into practice to optimize functional outcomes for hospitalized older adults: A scoping review
Source: PLoS One. 2025 Jun 16;20(6):e0324953. doi: 10.1371/journal.pone.0324953 (PMC12169582; doi:10.1371/journal.pone.0324953)
Supplement: S4 Table — (DOCX) [file pone.0324953.s004.docx]

**S-4 Table.**

*Table of Included Studies (N=38)*

| **Author (year) Country** | **Method, study aim, sample setting** | **Education component** | **Interventions in addition to education** | **Outcomes assessed and relevant findings** |
| --- | --- | --- | --- | --- |
| Allegri et al. (2022).  Italy | **Method**  Pre-post with group matching analysis (Quasi-experimental designs)  **Objective**  To evaluate whether a short training focused on improving dementia care practices of the hospital staff was able to counteract functional loss and to decrease negative outcomes at discharge  among hospitalized older adults with cognitive impairment.  **Sample**  68 hospitalized patients aged 65 years and over with cognitive impairment (intervention group: 34 and control group:34).  **Setting**  General Medicine, Surgery, and Orthopedics units | A five-hour training course, including five one-hour teaching modules were delivered to hospital staff (doctors, nurses, physical therapists and healthcare assistants) working in the medical units involved in the study.  Module 1 - improving the detection of signs and symptoms of dementia and cognitive impairment, depression, BSPD, and specific needs of the patients. Module 2 - drug prescription. Module 3 - management of BPSD and delirium., Module 4 - detection of pain. Module 5 - communication and interaction with patients. | **Level of intervention-** medium scale, unit based single hospital model of care.  **Care process change:** Italian Dementia-Friendly Hospital trial (IDENTITA) was implemented. Focus on cognitive impairment including dementia, delirium, depression-BSPD, medication, pain and communication | **Functional outcomes**  Functional status measured using modified Barthel index.  **Cognitive outcomes**  Cognitive impairment measured using MMSE. |
| Apolinario et al. (2022).  Brazil | **Method**  Prospective cohort program evaluation (design not reported).  (Quasi-experimental designs)  **Objective**  To describe the Safe Hospital for the Elderly Program and assess the initial results of its implementation in a philanthropic hospital in the city of Sao Paulo.  **Sample**  865 frail hospitalized older adults (582 in the implementation period and 283 in the consolidation period).  **Setting**  Philanthropic hospital | Education targeted to professionals to interpret the results of the frailty screening questionnaire and the AMPI (Multidimensional Assessment of the Elderly), and acquire basic knowledge about eight complications in the hospital environment: (1) fall, (2) pressure injury, (3) bronchoaspiration, (4) delirium, (5) mechanical restraint, (6) malnutrition, (7) medication iatrogenesis, and (8) functional decline.  Staff were also given resources such as booklet, video lessons, infographics, on-site small group training, and training workshops. | **Level of intervention-** medium scale, single hospital, model of care  **Care process change:** Implementation of the Safe Hospital Program for the Elderly developed by a multidisciplinary team. Frailty screening used to select patients. Multidisciplinary assessment that included cognitive, delirium, depressive symptom, ADLs, mobility, nutrition screening, risk of bronchial aspiration, medications at risk, communication and social support.  Protocols on prevention of falls, pressure injury, bronchial aspiration, prevention, detection and management of delirium and rational use of mechanical restraints, mobility stimulation, screening of inappropriate medications, approach to the elderly, and nutritional risk. Multidisciplinary rounds to discuss cases.  **Facilitation**  Nurse case manager  **Discharge planning or care transition planning**  Identification of factors related to long stay, map of de-hospitalization resources and development of a discharge planning program with emphasis on transitions of care. | **Functional outcomes**  Functional decline assessed by Barthel index.  Occurrence of one or more falls during hospitalization. |
| Bakker et al. (2014)  Netherlands | **Method**  Before-after study including mixed-methods process and an effect evaluation.  (Quasi-experimental designs)  **Objective**  To evaluate the implementation of the CareWell in Hospital program, which is an innovative intervention designed to prevent complications and stimulate early rehabilitation among frail elderly inpatients.  **Sample**  191 pre-intervention and 195 post-intervention  70 years and older  **Setting**  2 surgical wards and 1 internal medicine | Physicians and nurses receive group education from the geriatrics team on components of the CareWell in Hospital program including a coaching at work. | **Level of intervention-** medium scale, single hospital, model of care  **Care Process Change**  The CareWell in Hospital (CWH) program (based on the Hospital Elder Life Program (HELP) Inouye et al 2000) was developed and pilot tested.  Development of a care pathway for screening and management of patients at risk for geriatric syndromes.  Intervention included:  Screening, medication review, Carewell plan (recommendations about care and well-being) in: somatic problems, physical functioning, social environment, psychosocial functioning, and communication), follow-up, geriatric assessments, multidisciplinary meetings, therapeutic activities. Team of trained volunteers.  **Facilitation**  Coaching from the geriatric team (geriatric nurse specialist and geriatrician). | **Functional outcomes**  Physical functioning assessed using Groningen Activity Restriction Scale (GARS)  **Cognitive outcomes**  Delirium assessed by Confusion Assessment Method  Cognitive functioning assessed by Dutch version of the Mini-Mental State Examination (MMSE). |
| Boltz et al. (2014)  United States | **Method**  Comparative repeated measures design with intervention and control arm.  (Quasi-experimental designs)  **Objective**  To test the feasibility of  Family-centered Function-focused Care (Fam-FFC) and to examine the impact of Fam-FFCon functional outcomes of hospitalized older medical patients.  **Sample**  97 dyads (partnership between older patient and a caregiver)  **Setting**  Three medical units of a community teaching hospital | 1^st^ month nursing staff receive education session on prevention and management of delirium and functional decline; assessment of mood, cognition, and function; incorporation of function-focused care into care routines; ways to communicate, partnering, and motivating patients and family; and discharge planning.  On the 2^nd^, 3^rd^, and 4^th^ month, educational reminders where distributed to the intervention group through staff mailboxes and educational boards. | **Level of intervention-** medium scale, single hospital, model of care  **Care Process Change**  The implementation of Family centered function focused care (Fam-FFC) which includes environmental modification, education of family members, and involvement of patient and family in their plan of care from admission to after discharge.  **Environmental Changes**  Assessment and modifications on the environmental layout of the units such as lighting, seating, bed height, toilet height, communication boards for staff and patients, and accessibility of sensory and mobility aids.  **Administrative decisions**  Patient and/or family members are included in shift reports.  **Facilitation**  Family-centered resource nurse (FCRN) (RN with experience in gerontology and certified in gerontology) 10 hours a week. Nursing supervisor as unit champion for the study.  **Discharge planning/transition care**  Follow up by FCRN | **Functional outcomes**  ADL performance assessed by Barthel index (walking item to measure walking performance)  Gait and balance assessed by Tinetti Scale.  **Cognitive outcomes**  Delirium assessed by Delirium Severity Scale. |
| Bryant et al. (2019).  USA | **Method**  Retrospective cohort study  (Quasi-experimental designs, comparison of pre-post intervention groups)  **Objective**  To determine if an interdisciplinary care pathway for frail trauma patients improved in-hospital  mortality, complications, and 30-day readmissions.  **Sample**  269 patients frail patients aged ≥65 years admitted to the trauma service (Pre-intervention: 125 and post-intervention: 144)  **Setting**  Trauma center | Residents and physician assistants received an educational presentation and a pocket card for easy reference.  Educational efforts for all the nurses, ancillary staff, residents, and attending staff who frequently care for older patients in trauma units and in the ICU.  Mandatory educational modules were also developed. | **Level of intervention-** medium scale, single hospital, model of care  **Care process changes**  A standardized, evidence-based interdisciplinary pathway of care was developed and tested to reduce delirium and complications in frail geriatric trauma patients.  The domains of the pathway included: on the pathway for delirium prevention, mobility, medication management, prognostic assessment, reducing other complications, social needs/goal setting and injury prevention.  Patients 65 and older screened for frailty in ER. Standardized order set for patients (orders included early mobilization, nutrition, delirium prevention, identifying proxy, advanced directives and geriatric physio, nutrition and SW consults. All patients receive fall prevention education prior to discharge. Interdisciplinary bi-weekly rounds with trauma geriatrician.  **Administrative decisions**  **Facilitation**  Nurse champions.  **Discharge planning/transition care**  Interdisciplinary family meeting for hospital length of stay of more than 5 days. | **Cognitive outcomes**  Delirium assessed by Confusion Assessment Method. |
| Chang et al. (2007)  Australia | **Method**  Part of multi-phase design.  This study is comparative repeated measures design (Quasi-experimental design, comparison of pre-post intervention groups)  Note:  Part of a larger Mixed Method – Participatory Action Research including Chang (2003)  **Objective**  To compare the efficacy of a model of care for older adults when implemented to (a) an aged care specific ward and (b) a standard medical ward admitting older adults.  **Sample**  56 older adult patients from the aged care ward group and 60 older adult patients from the standard medical ward group  **Setting**  Acute Medicine Units | The education was delivered to nurses through in service type meetings over a span of 3-week period. The different models of care specific to older adults in acute care were discussed.  The key reference group provided feedback to other nurses, which facilitated ongoing learning among them. | **Level of intervention-** medium scale, single hospital, model of care  **Care Process Changes**  Nurses participated in action research phase to develop model of care for their unit Guided by Orem’s self-care and the NICHE medication protocol concepts. Interdisciplinary team on aged care ward included geriatrician. Communication among nurses about patients’ functional status and knowledge medication.  **Environmental Changes**  Physical environmental modifications were implemented to address the need of older adults such as railings for fall prevention and patients assigned closer to nursing stations.  **Facilitation**  Each ward created a key reference group of 3 to 5 nurses, who collaborated with the research team to tailor the model to the context of their ward as well as facilitate sharing of knowledge between nursing staff. | **Functional outcomes**  ADL assessed by Barthel index. |
| Cohen et al. (2019).  Israel | **Method**  A quasi-experimental pre-post two group comparative design.  **Objective**  To examine the effect of WALK-FOR on the prevention of hospital-associated functional decline (HAFD) at discharge and at 1-month follow-up from acute hospitalization in internal *medical units.*  **Sample**  Intervention group: 188  Control group: 189  **Setting**  Two hospital internal medical units, each unit consisting 46 bed-in a medical center | Staff (nurses and nurse aides) underwent theoretical and  practical training to assess mobility and to safely mobilize patients. | **Level of intervention-** small scale, single hospital, single focus  **Care process changes:**  Implementation of the WALK -FOR mobility program  Unit-tailored mobility programs were structured for unit staff, patients, and their caregivers, which were distributed through in-personal communication, video clips, brochures, and posters.  Nurses assessed mobility and provided mobility recommendations at admission.  **Environmental**  Environmental modifications included removing physical obstacles from corridors, marking walking trails, and purchasing extra walkers.  **Administrative decisions**  Electronic medical records were modified to include the patient's mobility ability as well reports on daily walking distance. | **Functional outcomes**  Individual’s subjective assessment of their independence level in performing basic activities of daily living (BADL) operationalized as decrease of 5 points or more in BADL score, assessed by Modified Barthel Index.  Instrumental Activities of Daily Living (IADL) assessed using Lawton’s IADL eight item scale for premorbid status.  Community mobility assessed using an activity summary index (subscale of Yale Physical Activity Survey (YPAS). |
| Gazineo et al. (2021)  Italy | **Method**  Randomized Controlled Trial  **Objective**  To evaluate if an individualized assisted walking program (IAWP) for hospitalized older patients could improve walking ability compared with usual geriatric care and rehabilitation.  **Sample**  Of the total 387 included patients (193 were randomly assigned to the intervention group and 194 to the usual/control group.  **Setting**  32-bed geriatric unit of a university hospital in Bologna, Italy. | One nurse received training in the walking protocol and patient and family education (not the full team). Very limited description of training. | **Level of intervention-** small scale, single hospital, single unit, single focus  **Care process changes: Mobility**  Individualized assisted walking program (IAWP) was delivered to an intervention group by a trained nurse. The IAWP program included early assessment within 24 hours of admission, moving from the supine to sitting position and from sitting to standing position, supervised ambulation and education about the importance of daily ambulation for patients and their caregivers.  Intervention group patients were helped to move from the supine to sitting position with their legs hanging over the side of the bed, from sitting to standing and an active phase of walking with assistance. Intervention was carried out in daily basis (except weekends) for 20-30 minutes and continued from the first day of admission until the day before discharge.  **Facilitation**  A daily briefing session was held between the trained nurse and the geriatrician in charge prior to intervention to assess the feasibility for each patient. | **Functional outcomes**  Change in walking ability (hospital admission to discharge) assessed with Braden Activity subscale (BAS). |
| Gil et al. (2023).  Israel | **Method**  Quasi-experimental three-group comparative design (pre-implementation usual care, immediate intervention and 2 year post implementation groups) .  **Objective**  To explore whether the WALK-FOR (walking for better outcomes and recovery) intervention’s effect on the daily step count of acutely ill older adults is sustainable 2 years after its implementation.  **Sample**  (pre-implementation or control group: 150; immediate post-implementation: 144 and 2-year post implementation: 72)  **Setting**  46-bed internal medicine units in a medical center in Northern Israel. | Unit staff, including nurses, nurses’ aides and physical therapists received theoretical and practical training on the WALK-FOR intervention program with the aim to raise knowledge and awareness of mobility during hospitalization.  Note: Education component reported in original report Cohen et al. (2019) (cited by authors) | **Level of intervention-** small scale, single hospital, single unit, single focus  **Care process changes**  The WALK-FOR intervention program was designed to promote in-hospital mobility of patients.  Patients were asked to walk on the marked trail  along the corridor at least twice daily in order to complete at least 900 steps. Patients wore accelerometers (Actical) on the ankle which were used to monitor daily step counts up to 3 days.  **Environmental**  Physical obstacles were removed from corridors, walking trails were marked.  **Administrative decisions**  Extra-walkers were purchased. | **Functional outcomes**  Mobility during hospitalization was defined as the average daily number of steps (monitored by ankle-worn accelerometers) Self-reported range and frequency of their mobility inside and outside of their rooms. Yale Physical Activity Survey activity summary index to report Community mobility.  Also measured ADL by Modified Barthel index, IADL by Lawton IADL scale. |
| Hamilton & Lyon (1995).  Canada | **Method**  A quasi-experimental, one-group pretest/post-test design  **Objective**  To evaluate effectiveness of a 6 bed module for geriatric assessment and rehabilitation patients’ functional status.  **Sample**  74 patients aged 65 years and older with medical stability (did not require bed rest) and functional impairment in one or more areas of ADL.  Those with contractures (affecting mobility) and severe cognitive impairment were excluded.  **Setting**  6 bed geriatric assessment and rehabilitation module within a 70 bed medical unit in a 258 -bed community hospital | Geriatric module included three resource manuals: (1) Urinary continence in the elderly: A nurse program, (2) Improving functional mobility in the elderly: A nursing program, and (3) The geriatric module manual (included policies and procedures).  Staff who rotated in the geriatric module for 8-week periods were oriented to the module and the study.  Staff received education on urinary incontinence and mobility programs from clinical nurse specialists (service purchased from another hospital for 18 month development and support period) . | **Level of intervention-** small scale, single hospital, single unit, 2 foci  **Care process changes**  Mobility and urinary incontinence program delivered by nurses.  **Administrative decisions**  Enhanced nursing resources during day shift in 6 bed module (1.5 FTE added); medical unit staff covered evenings and nights.  **Facilitation**  Clinical nurse specialist available on site 1 day per week. Nurse manager implemented modular nursing and provided consistent administrative support. | **Functional outcomes**  Functional status assessed using Barthel index of activities of daily living at admission to module from medical unit, every two weeks and discharge.  **Cognitive outcomes**  Cognitive function assessed by MMSE. |
| Heim et al. (2016)  Netherlands | **Method**  Pre-program vs post program implementation  (Quasi-experimental designs)  **Objective**  Report on development, implementation and evaluation of a regional transitional care programme aimed  at improving the recovery rate of frail hospitalized older patients (70+).  **Sample**  Data on functional decline were complete for 721 (frail) and 827 (non-frail)  patients who filled out and returned the self-administered  questionnaire after 3 months of follow-up.  **Setting**  Four hospitals working with regional health care organizations (nursing home, rehabilitation centre) | Educational component for HCPS - two aspects:  Nursing/medical staff were educated on frailty in older patients.  The trained nurses collaborated on the units to educate staff  to facilitate rehabilitation. They organized clinical lessons, but mostly trained on the job.  Staff of the rehabilitation unit was educated to be better able to care for early discharged patients to enable patients to be transferred  to the well suiting unit sooner. | **Level of intervention-** large scale, healthcare system, model of care  **Care Process Change -**  A screening tool (ADL, undernutrition, falls, delirium) and triage instrument for frailty and related protocols were developed, developed for the hospital component. In hospital Geriatric consultation teams to follow-up on screening results. Care protocols for nurses (details not provided). Weekly multidisciplinary meetings  focused on improving/preserving ADL functioning.  **Environmental**  No hospital environment changes. Nursing home unit (discharge destination option) refurbished to meet needs of patients with cognitive disorders and a rehabilitation need.  **Administrative decisions**  Regional interorganizational program development, implementation, evaluation  Multi party commitment to the transitional care program.  **Facilitation**  Every unit appointed a nurse responsible to implement the protocols in his/her unit.  Each organization appointed staff member responsible for the internal implementation of a digital transfer system.  **Discharge planning or care transition planning**  Triage instrument for best discharge destination and follow up care.  Links to rehabilitation unit  outpatient unit in GP Office, minimum data set for transfers developed (digital transfer, shared expertise between hospital, nursing home, rehabilitation centre | **Functional outcomes**  ADL functioning assessed using Katz index on independence.  Nutritional status assessed using short nutritional assessment questionnaire (SNAQ)  Composite endpoint of adverse outcome (ADL function and/or high health care demand 3 months after hospital admission)  **Cognitive outcomes**  Risk of delirium assessed using researcher developed questions. |
| Holt et al. (2013).  UK | **Method**  Before and after study  (Quasi-experimental designs)  **Objective**  To examine the effect of a multi-component, delirium prevention intervention on rates of incident delirium for  patients admitted to specialist elderly care wards.  **Sample**  436 patients (Before group: 249 and After group: 187)  **Setting**  Three specialist elderly care wards (each 80-bed) in a general hospital | The research team provided education and practice change materials to the ward staff (staff nurses, healthcare assistants). Educational - 30 min interactive lecture with a handout, a delirium quiz, a poster, reference material and case vignettes.  Practice change materials - delirium risk factor modification care plan, a delirium assessment protocol for ward doctors and an escalation flowchart for suspected delirium for nurses. | **Level of intervention-** small scale, single hospital, 3 units, single focus  **Care process changes**  Delirium prevention/ intervention on specialized units consisted of standardized education and delirium, risk factor modification protocol materials. Practice change included delirium risk factor modification care plan placed at end of patient bed, required signed actions 3 times a day. Delirium assessment protocol for ward physicians and escalation flow chart of suspected delirium for nurses.  **Facilitation**  Specialist nurse, consultant geriatrician and nurse manager. Volunteer nurse from each ward group acted a “link” | **Functional outcomes**  Function at discharge assessed using Barthel index score.  **Cognitive outcomes**  Delirium was assessed by daily assessments and CAM (Confusion Assessment Method) and DRS-R-98 (Delirium Rating Scale-Revised-98) instruments. |
| Inouye et al. (1993b). A controlled trial of a nursing-centered intervention in hospitalized elderly medical patients: The Yale Geriatric Care Program.  USA | **Method**  Prospective cohort study with stratified and matched cohort analyses.  (Quasi-experimental designs)  **Objective**  To test the effectiveness of a nursing-centered intervention to prevent functional decline among hospitalized  elderly medical patients. *(Yale Geriatric Care Program)*  **Sample**  216 patients aged ≥70 years (intervention group: 85 and control group: 131)  **Setting**  Five general medicine wards of a 800 -bed urban teaching hospital (Yale-New Haven Hospital). Two intervention units (one was “nurse and physician intervention, the other was “nurse only intervention” plus three control units). | Geriatric resource nurses were provided special training and education in geriatric nursing and were key intervention figures. In addition, unit-based geriatric lecture series were provided for all nurses.  NOTE: Education component described in model protocol/description Inouye et al. (1993a) (published in same journal issue) | **Level of intervention-** medium scale, single hospital, units, model of care.  **Care process changes**  The Yale Geriatric Care Program designed to develop and integrate geriatric nursing expertise as a part of standard nursing care on each intervention unit.  Nurse/physician Intervention included identification of frail older patients with subsequent daily surveillance, twice-weekly rounds of the geriatric care team (nurse focused interventions), special education and support for the geriatric resource nurses, and a unit-based geriatric lecture series for all nurses.  Nurse-only intervention’ which was the same as above, except that the geriatric physicians did not participate in rounds.  Clinical protocols to address six targeted conditions (Geriatric Vital Signs)- Skin impairment, undernutrition, incontinence, confusion (delirium/dementia), falls or functional impairment, sleep disturbance.  **Facilitation**  Geriatric resource nurses (GRN), a masters-prepared gerontological nurse specialist (GNS), and two geriatric physicians. Geriatric Care Team (Geriatric resource nurses, gerontological nurse specialist, geriatric physician, primary nurses of involved patients) twice weekly rounds.  **Discharge Planning** - included in nursing education details not provided | **Functional outcomes**  Pressure ulcer assessed using standardized skin check.  Patients self-reported their ability to perform ADLs, information on incontinence and social activity level.  **Cognitive outcomes**  Cognitive impairment was assessed by MMSE  Delirium by Confusion Assessment Method. |
| Inouye et al. (1999).  USA | **Method**  Controlled clinical trial  (Quasi-experimental designs)  **Objective**  To evaluate the effectiveness of multicomponent strategy for the prevention of delirium *(Elder Life Program)*  **Sample**  N= 852 patients assessed as at risk for delirium, matched as 426 pairs of patients receiving the study intervention and usual care.  **Setting**  800-bed urban teaching hospital with 200 medical beds. Study took place on one intervention medical unit and two usual care medical units | Research nurses and experienced clinical researchers received intensive training and followed standard procedures outlined in a detailed training and coding manual on delirium risk factors-cognitive impairment, sleep deprivation, immobility, visual impairment, hearing impairment and dehydration. | **Level of intervention-** medium scale, single hospital, 1 unit, model of care.  **Care process change**  Elder Life Program was implemented by a trained interdisciplinary team and volunteers.  Intervention protocol focused on 6 target risk factors for delirium (cognitive impairment, sleep deprivation, immobility, visual impairment, hearing impairment, dehydration) and included orientation protocol, therapeutic-activities protocol, nonpharmacologic sleep protocol (warm drink, relaxation music and back massage at bedtime), early mobilization protocol, vision protocol, hearing protocol, dehydration (including early recognition), protocol to improve cognition, sleep, mobility, vision, hearing, and fluid intake respectively. Cognitive simulation activities included discussion of events, structured reminiscence, or word games.  **Environmental**  Board with names of care-team members and daily schedule kept as orientation protocol. Noise reduction strategies: Maintaining quiet hallways, silent pill crushers, vibrating beepers, rescheduling medications and procedures. Adaptive equipment (large print books, fluorescent tape on call bell)  **Administrative decisions**  Minimizing use of immobilizing equipment (e.g., physical restraints), encouraging ambulation.  Accessible portable amplifying devices, special communication techniques.  **Facilitation** Team included of geriatric nurse-specialists, two specially trained Elder Life Specialists, a certified therapeutic recreation specialist, a physical therapy consultant, and a geriatrician. | **Functional outcome**s  Activities of Daily Living assessed using Katz’s index of activities of daily living.  **Cognitive outcomes**  Delirium assessed using Confusion Assessment Method. |
| Inouye et al. (2000)  United States | **Method**  Cohort (not reported, but seems like cohort).  (Quasi-experimental designs)  **Objective**  To describe the *Hospital Elder Life Program (HELP),* a model of care designed to prevent functional and cognitive decline of older persons during hospitalization. Specifically, describe: (1) the roles and responsibilities  of key personnel; (2) the setting and administrative structure· (3) the process of care -including interventions, screening; and enrollment procedures, the intervention process  and adherence rates, and quality assurance measures; (4) the outcomes and benefits of the program; (5) program costs and sources of funding; and (6) issues pertinent to dissemination  to other hospitals.  **Sample**  1507 patients aged 70+.  **Setting**  Three general medicine nursing units at Yale-New Haven Hospital (800-bed urban teaching hospital with 200 medical beds) | Education program delivered by Elder Life nurse specialist, geriatrician and nurse  practitioners to nurses, physicians and volunteers about Elder Life issues- included orientation protocol, therapeutic activities, early mobilization, vision/hearing, oral volume repletion, feeding assistance, sleep enhancement, geriatric nursing assessment and interventions.  Formal didactic sessions, small group training, and resource materials. | **Level of intervention-** medium scale, single hospital, 3 units, model of care.  **Care process changes**  Implementation of the Hospital Elder Life Program. All patients aged ~70 years on specified units screened on admission for 6 risk factors (cognitive impairment, sleep deprivation, immobility, dehydration, vision or hearing impairment). Targeted  interventions for these risk factors (daily visitor/orientation, therapeutic activities (cognitive stimulation), early mobilization (ambulation or active range of motion 3 times daily), vision protocol, hearing protocol, oral volume repletion, feeding assistance, sleep protocol (e.g rescheduling medications and procedures) implemented by an interdisciplinary team that included Elder Life Specialists (see Facilitation below) , trained volunteers, and geriatricians who work closely with primary nurses. Interdisciplinary team for other interventions, consultation expert at twice-weekly interdisciplinary rounds for each patient. Recommended interventions tracked.  **Environmental**  Orientation board with names of care team members and daily schedule. Unit-wide noise-reduction strategies (e.g. silent pill crushers, vibrating beepers, and quiet hallways).  **Administrative decisions**  Monitoring of intervention adherence (quality assurance).  **Facilitation**  Key HELP staff included Elder Life Nurse Specialist, Elder Life Specialist/volunteer coordinator, geriatrician, program director (one of above). Geriatrician consultation Interdisciplinary consultation as needed. Volunteer recognition incentive awards.  **Discharge planning or care transition planning**  Discharge communication form. Community linkages and telephone follow-up. Referrals and communication with community agencies to optimize transition home. Telephone follow-up within 7 days after discharge. | **Functional outcomes**  Functional decline (tool not given)  **Cognitive outcomes**  Delirium (tool not given). |
| Inouye et al. (2006).  USA | **Method**  Cross-sectional survey  **Objective**  To describe the Hospital Elder Life Program  (HELP) across dissemination sites, to detail adaptations,  and to summarize advantages across sites.  **Sample**  11,344 patients  **Setting**  13 HELP sites (academic teaching hospitals) | Volunteers were provided education sessions and supervision by paid staff about the HELP model. | **Level of intervention-** Large scale, 13 hospitals, model of care.  **Care process change**  The HELP program was implemented at academic teaching hospitals.  The HELP team included The Elder Life Nurse Specialist implemented nursing-related assessments and protocols targeted toward the six delirium risk factors.  Volunteers assisted implementation in HELP model interventions. Sites purchased implementation packages (program manuals, business tools, training video tapes/CD, tracking software). There was a help website for their support.  Regular interdisciplinary rounds were available at 69.2% of sites and geriatric consultation at 92.3% of sites.  **Environmental**  Not described but part of program materials.  **Facilitation**  Elder Life Specialist, Elder Life Nurse Specialist, and geriatrician. The Elder Life Specialist is the program and volunteer coordinator. The Elder Life Nurse Specialist is an advanced practice nurse with specialized training in geriatrics  Regular monitoring and educational sessions for volunteers were available to enhance their performance.  **Discharge planning or care transition planning**  38.5% of sites conducted post-discharge telephone follow up. | **Functional outcome**s  Activities of daily living, falls were assessed. Tool not reported.  **Cognitive outcomes**  Cognitive function assessed using Mini Mental State Examination (MMSE).  Delirium assessed (tool not reported). |
| Juneau et al. (2018).  Canada | **Method**  Feasibility study, cross-sectional study  **Objective**  To assess the feasibility of implementing the SPecific Retraining in INTerdisciplinarity (SPRINT) in the context of a Geriatric Assessment Unit (GAU).  **Sample**  19 patients  **Setting**  Geriatric Assessment Unit of a hospital | Geriatric assessment unit (GAU) professionals (nurses, physicians) received 20 min group training on SPRINT prior to study (No more details in education component). | **Level of intervention-** small scale, 1 hospital, 1 unit, single focus.  **Care process changes**  SPRINT included four exercise categories according to a level of mobility. The categories range from patient completing exercises alone to requiring professional supervision. Visual and verbal reminders to professionals and encouragement to participants and caregivers.  **Facilitation**  Physiotherapist and coaches (a registered or ancillary nurse for day shift and a care attendant for evening shift). | **Functional outcomes**  Physical dependency for Activities of Daily Living (ADL) using Functional Autonomy Measurement System (SMAF)  Fall risk using Berg Balance scale  **Cognitive outcomes**  Cognitive state using Mini Mental State Examination (MMSE) |
| King et al. (2016)  UK | **Method**  Cross-sectional study  **Objective**  To determine whether all components of MOVIN (Mobilizing Older adult patients VIa a Nurse-driven intervention) can be implemented simultaneously and to test whether there was a change in nursing practice and unit culture after the intervention on one inpatient unit. To evaluate whether the identified measures were appropriate to detect practice and culture change.  **Sample**  18 RNs completed the training; all nursing staff (32 RNs and 10 CNAs) were exposed to the MOVIN intervention; 10 RNs and 5 CNAs agreed to participate in focus groups and 1 RN participated in a single person interview.  **Setting**  25 bed adult general medical unit at a 648-bed tertiary academic teaching hospital. | RNs and certified nursing assistants received psychomotor skills training.  Two PTs (physical therapists) provided psychomotor skills training using an ambulation skills training program that were created by a researcher and a PT collaborator. | **Level of intervention-** small scale, 1 hospital, 1-unit, single focus.  **Care process changes**  MOVIN mobility program implemented. Communication board and ambulation pathways.  Implementation of the mobility aide role occurred on the first day of the intervention.  **Environmental**  White boards used to share information on patient care.  Visual markers used to measure distances that patients ambulate.  **Administrative decisions**  Ambulation resources (human and equipment) to maximize opportunity for patient ambulation, and ambulation culture to establish nurse ownership and sustainability of patient ambulation.  **Facilitation**  Mobility aide role. Weekly incentives such as drawings for gifts cards and unit celebrations when ambulation goals were met were implemented. | **Functional outcomes**  Ambulation frequency (total number of ambulation occurrences on the unit per week), ambulation distance (total distance in feet of patients ambulated on the unit per week) assessed by observation and communication with patient. |
| Kratz et al. (2015)  Germany | **Method**  Pre-post  (Quasi-experimental designs)  **Objective**  To answer the  following questions:  ● What is the incidence of postoperative delirium in  a general surgical ward in a general hospital?  ● What preoperative factors are predictive of  delirium?  ● Can a specialist geriatric psychiatric nurse reduce  the incidence of postoperative delirium by means  of non-pharmacological interventions?  **Sample**  N= 125 for the prevalence phase, for the intervention phase N = 114 (61 in intervention group, 53 in control group.  **Setting**  Two surgical wards or a teaching hospital delivering standard care (both wards underwent the prevalence phase, but during the intervention phase one ward became the intervention group and the other the control group). | A nurse with special training in the management of delirium (“delirium liaison nurse”) carried out an intervention involving component measures of the Hospital Elder Life Program (HELP) on one of the two wards, with the aim of preventing postoperative delirium. Validation included a training program for nursing staff as a form of  self-preservation therapy. | **Level of intervention-** medium scale, 1 hospital, 2 units, model of care.  **Care process changes -**  Delirium liaison nurse delivered interventions pre- and postoperatively, according to the individual needs of patients, in the following areas:  ● Early mobilization  ● Improved sensory stimulation  ● Improved fluid and nutritional intake  ● Non-drug sleep improvement  ● Cognitive activation  ● Validation  **Environmental**  Lighting to improve sensory stimulation, aids (clocks, calendar, daily newspaper) for reorientation.  **Facilitation**  Delirium liaison nurse supported delivery of interventions including education for relatives on cognitive activation. | **Functional outcomes**  **Cognitive**  Mini–Mental State Examination Score, using NOSGER. Risk **Physical**  Barthel Index. |
| Liu et al. (2018).  Canada | **Method**  Pragmatic, quasi-experimental interrupted time series (ITS) design.  (Quasi-experimental designs)  **Objective**  To implement and evaluate an evidence-based strategy targeting staff to promote early mobilization in older hospitalized  patients.  **Sample**  12,490 patients (Pre-intervention: 3,318; during intervention: 2,786 and post-intervention: 6,386).  **Setting**  11 University affiliated hospitals | Study team provided hospitals with resources (education modules, checklists, mobility algorithms) to implement the intervention and invited them to use or adapt these, or develop new materials.  Local implementation teams at each hospital included a physician, education coordinator, and research coordinator. | **Level of intervention-** medium scale, 11 hospitals, single focus.  **Care process changes**  Mobilization of Vulnerable Elders in Ontario (MOVE ON)  Intervention focused on 1) patients being assessed for mobilization within 24 hours of admission, 2) mobilization 3 times a day, 3) mobility to be progressive and scaled, tailored to patients’ abilities, All hospitals had access to an online community of practice and collaborated in monthly teleconferences.  **Facilitation**  Local education coordinator, physician champion, and research coordinator facilitated implementation of intervention and led/supported the local working group. Implementation coaches worked with local implementation teams to select intervention strategies mapped to identified barriers and facilitators as well as provided support. | **Functional outcomes**  Patient considered mobilized, if out of bed during visual audit. |
| Martinez-Velilla et al. (2016).  Spain | **Method**  Cohort. Prospective intervention study with blinded outcome progression  (Quasi-experimental designs)  **Objective**  To assess if an exercise intervention involving patients and families could modify the  cognitive and affective progression of hospitalized older  patients, from admission to discharge and 30 days after discharge.  **Sample**  29 patients (17: intervention and 12: control).  **Setting**  Geriatric ward of a tertiary hospital | Ward and research team staff were educated to actively encourage mobility and functional independence (Not explained in detail). | **Level of intervention-** small scale, 1 hospital, 1 unit, single focus.  **Care process changes**  Individualized graduated exercise programs were prescribed and supervised by an exercise physiologist and the unit physiotherapist. Patients and caregivers were educated to encourage mobility and functional independence. Participants had to exercise 30-40 min 3 times daily.  **Discharge planning or care transition planning**  Participants continued exercise after discharge for 1 month later, recorded in a calendar and reviewed during follow-up medical visit. | **Functional outcomes**  Dependence in daily activities was assessed using Barthel index. Nutritional status by the mini-nutritional assessment (MNA).  **Cognitive outcomes:**  Dementia assessed using Global deterioration scale (GDS).  Mental function evaluation using the Spanish version of the Folstein Mini-Mental State Examination.  Delirium assessed by the Spanish version of the Delirium Rating Scale Revised-98 and Confusion Assessment Method (CAM).  Depression assessed using Spanish version of the Yesavage abbreviated questionnaire (GDS-Y)  Cognitive processing speed using Trail Making Test. |
| Milisen et al. (2001).  Belgium | **Method**  Longitudinal prospective before/after design (Quasi-experimental designs)  **Objective**  To develop and test the effect of a nurse led interdisciplinary intervention program for delirium on the incidence and course (severity and duration) of delirium, cognitive functioning, functional rehabilitation, mortality, and length of stay in older hip-fracture patients.  **Sample**  60 patients in intervention cohort and 60 patients in a usual care/non intervention cohort.  **Setting**  The emergency department of the university hospitals of Leuven and two traumatological units of an academic medical center | Educational poster placed in the emergency department and the traumatological to educate all nurses on assessment, pathophysiology, diagnosis and treatment of delirium, depression, and dementia. Poster included (1) core symptoms of delirum according to the CAM (Confusion Assessment Method), (2) comparative features, and differences between delirium, dementia, and depression, and (3) the relevance of correct and early recognition of delirium.  All nurses were trained in using the NEECHAM Confusion Scale – screening instrument to assess acute confusion/delirium.  Nurses were recruited as resource nurses by a geriatric nurse specialist. Resource nurses were given special training in the identification and management of older hip-fracture patients at risk for delirium. | **Level of intervention-** medium scale, 1 hospital, 2 units, single focus.  **Care process changes**  **Model of Care Change**  An integrated model of geriatric nursing care  for enhanced quality of nursing care for older hip fracture patients. Nurses identified high risk patients and provide prompt anti delirium interventions, provided anti-delirium interventions from a nursing guide for evaluation of causes of delirium in older hospitalized patients (details not given) although regular administration of pain medications (a short acting weak opioid in combination with a nonopioid analgesic such as acetaminophen) was identified.  **Facilitation**  8 Specially trained unit-based resource nurses all of whom were prepared at the graduate level. Consultation with a geriatric nurse specialist or a psychogeriatrician if needed. | **Functional Outcomes:**  Activities of Daily Living (ADLs) using Katz Index of Activities of Daily Living.  **Cognitive Outcomes**  Delirium using the English version of the CAM (Confusion Assessment Method).  Acute confusion using NEECHAM Confusion scale.  Cognitive status using MMSE (Mini Mental State Examination). |
| Miller et al. (2004).  USA | **Method**  Pre-post, quasi experimental design  (Quasi-experimental designs)  **Objective**  To test the Elder Care Supportive Interventions Protocol (ECSIP),  which was designed to reduce the discomfort of confused, hospitalized  older adults and the associated negative  consequences of delirium,  impaired physical function, and increased need for post-hospital  care; and to foster the interest of undergraduate nursing students in  gerontological nursing practice.  **Sample**  Pre-intervention: 81 patients (control: 38 and intervention: 43) and post-intervention: 52 patients (control: 20 and intervention: 32) Nursing staff from the study unit participated.  **Setting**  A 34-bed geriatric medical unit and a 32-bed orthopedic/trauma surgical unit of a tertiary care, academic hospital. | All nursing staff in the participating unit were provided a 2-hour education program. The education program included significance of confusion and discomfort with hospitalized older adults, the purpose and design of the study, and examination of the Elder Care Support Intervention Protocol (ECSIP).  Undergraduate nursing students received basic undergraduate coursework regarding physical care, assessment of basic needs, patient safety, and therapeutic communication. | **Level of intervention-** medium scale, 1 hospital, 2 units, model of care  **Care process change**  Elder Care Support Intervention Protocol (ECSIP) included four components: staff education, implementation team, elder care assistants (ECAs) and environmental props.  Patients’ information (routines of rest and activity, food preferences, diversional activities, personal history, etc.) obtained from primary caregivers within 24 hours of admission was used to individualize care protocol. Interventions included prevention and control of discomfort and maintaining a familiar environment with meaningful communication and sensory input. Family caregivers were provided with a thank you for coming booklet to encourage their participation in care  **Environment**  Environmental props (no detail provided)  **Facilitation**  Nurse managers and clinical nurse specialists designed and supported staff in implementation. Formal and informal meetings were held re individual patient progression. | **Functional outcome**s  Physical function using Katz index of ADL. Discomfort using the modified Discomfort Screen-Dementia Alzheimer’s Type (DS-DAT)  **Cognitive outcomes**  Severity of delirium assessed by NEECHAM confusion scale. |
| Mudge et al. (2008).  Australia | **Method**  Prospective controlled trial with blinded outcome evaluation  (Quasi-experimental designs)  **Objective**  To evaluate the effect of a structured, multicomponent,  early rehabilitation program on functional  status, delirium, and discharge outcomes of older acute  medical inpatients.  **Sample**  124 patients (Intervention group: 62 and control group: 62)  **Setting**  Internal medicine department of a metropolitan, tertiary public teaching hospital | Ward nurses educated on deconditioning risks and benefits of encouraging independence.  Group in-service. | **Level of intervention-** medium scale, 1 hospital, units, model of care  **Care process changes**  The multicomponent, early rehabilitation program included: a graduated exercise program prescribed and supervised by the unit physiotherapist and multidisciplinary team staff encouraged mobility and functional independence Cognitive interventions delivered by psychology students supervised by a senior psychologist. Information resources included a walking map of ward and surroundings. Socialization, orientation and memory activities conducted.  Daily patient review and grand rounds. Patients provided with diary to record daily activity  program  **Facilitation**  Recognition and fostering of clinical champions. | **Functional outcomes**  Change in functional status (admission and discharge) using modified Barthel index (MBI).  Change in mobility (admission and discharge) using the timed up-and-go test.  **Cognitive outcomes** Delirium identified through chart review. |
| Mudge et al. (2020).  Australia | **Method**  Prospective pre-post (Quasi-experimental designs)  **Objective**  To implement a multidisciplinary co-management model for older vascular  patients and evaluate impact on length of stay (LOS), delirium incidence, functional  decline, medical complications and discharge destination.  **Sample**  Pre-intervention: 112 patients and post-intervention: 123 patients.  **Setting**  23-bed vascular surgical ward in a publicly funded metropolitan teaching hospital. | This study reported that allied health assistants were trained for smooth implementation of “Eat Walk Engage” program.  NOTE Education component described in model protocol/description Mudge et al. (2015) (cited by authors) | **Level of intervention-** medium scale, 1 hospital, 1 unit, model of care  **Care process change**  Eat Walk Engage Intervention included physician reviewed all patients over 65 years and younger patients with complex comorbidities, prioritized cases based on daily discussion with the nursing team; experienced clinical facilitators implemented the model of care to enhance nutrition and hydration, mobility and meaningful cognitive activities of patients. Daily physician rounds, huddles and weekly discharge planning meetings.  **Environmental**  Orientation boards  **Facilitation**  Facilitators involved a local multidisciplinary work group (MDWG), identified care practices for older patients, supported reflection on practice to prioritize improvements; and supported small improvement cycles.  Monthly MDWG meetings were held to encourage team communication and shared goals. | **Functional outcome**s  Functional status measured as the number of basic ADL (bathing, dressing, mobility, transfer, toileting and feeding) documented by nursing staff.  **Cognitive outcomes**  Delirium assessed using Confusion Assessment Method. |
| Mudge et al. (2022)  Australia | **Method**  Cluster randomized CHERISH (Collaboration for Hospitalized Elders Reducing the Impact of Stays in Hospital) trial  (RCT)  **Objective**  To implement and evaluate a ward-based improvement program (“Eat Walk Engage”) to more consistently deliver age-friendly principles of care to older individuals in acute inpatient wards.  **Sample**  539 consecutive  inpatients aged 65 years or older, admitted for 3 days or more to study wards.  **Setting**  8 acute  medical and surgical wards in 4 public hospitals. | A nurse or allied health professional from within each hospital was employed for 2 days per week as a site facilitator and were trained and mentored by 2 experienced facilitators on Eat Walk Engage program –that focused on nutrition and hydration, mobility, meaningful cognitive and social engagement and in multidisciplinary teamwork.  Site facilitators engaged staff to form a multidisciplinary working group (nurse unit manager, nurse educator, physiotherapist, dietitian, and occupational therapist) that reviewed local interview and audit findings to prioritize areas for improvement aligned with key principles and program goals (met for 1 hour per month and worked with the facilitator and other staff as required).  3D-CAM (Confusion Assessment Method) training videos were also provided. | **Level of intervention-** medium scale, 4 hospitals, 8 units, model of care  **Care process changes –**  CHERISH (Collaboration for Hospitalized Elders Reducing the Impact of Stays in Hospital), also called the EAT WALK ENGAGE program. Focused on consistent delivery of nutrition and hydration, mobility and cognitive/social engagement as well as interdisciplinary teamwork. Improved the patient lounge as walking destination, maps and signage to assist navigation, and chairs. Nursing assistant shift times changed to support meals. Nurse assistant/PT/OT to assist patients into chairs to eat. Nurses revise workflow at meal time.  **Environmental**  To support cognition  orientation boards and purchases and resources (eg, puzzles, games, pencils, books, glasses, cognitive resource cupboard, daily newspaper).  Note: Intervention strategies listed in article referred to as “examples” so may not be comprehensive.  **Facilitation**  On each ward, a nurse or allied health professional, acted as site facilitator  **Administrative decisions** | **Functional outcomes**  Composite measure of hospital-associated complications of older people (HAC-OP) such as delirium, disability, hospital-associated incontinence, fall or pressure injury  **Cognitive outcomes**  Delirium assessed by Confusion Assessment Method. |
| Mudge et al. (2023).  Australia | **Method**  Prospective multi-method implementation evaluation  (Quasi-experimental designs)  **Objective**  To understand how Eat Walk Engage worked across sites by (i) describing context, implementation and improvements in each site; (ii) describing and analyzing changes in process measures, (iii) describing how facilitation supported tailored implementation as hypothesised by the underpinning i-PARiHS implementation framework and (iii) proposing how key contextual features of different sites may have contributed to implementation success or failure.  **Sample**  In-patients on the four intervention wards, and multidisciplinary work group members and facilitators.  **Setting**  Four hospitals (2 inner metropolitan, 1 outer metropolitan, 1 regional) | Experienced facilitators provided four half-day initial group training sessions for site facilitators.  Training included didactic and interactive content based on the i-PARiHS facilitation guide, evidence for age-friendly care principles and the prevention and management of hospital-associated complications and provision of key readings.  Allied health or nursing assistants (selected by interview) were provided two weeks of training in care of older people, including instruction manual, face-to-face training with local allied health professionals (e.g., physiotherapist, speech pathologist) and work shadowing with an experienced assistant. | **Level of intervention-** medium scale, 4 hospitals, 4 units, model of care  **Care process changes**  **Model of Care Change**  Eat Walk Engage Programme focused on improving care of older people.  Allied health or nursing assistants set patients up for meals, supervised mobility or exercise activities, provided assisted listening devices, or updated orientation boards.  **Environmental**  Orientation boards  **Administrative decisions**  Project funding supported 24 hour/week experienced facilitator time. to support project management and external facilitation across sites. In addition, 16 hour/week site facilitator plus 20 hour/week multi-professional assistant for each implementation ward.  **Facilitation**  Mentoring included monthly half-day face-to-face peer group meetings.  Telephone and email support were provided between meetings, supporting debriefing, reflection on practice and shared learning.  Experienced facilitators visited. | **Functional outcomes**  Patients were assessed for eating patterns by cross-sectional structured mealtime observations. Observers recorded whether patients required assistance with meal set-up or eating, and whether they received this assistance within 10 minutes.  Observer also noted whether each patient was sitting up when the meal arrived, had the tray table in reach, and was interrupted during the meal. Percentage of mealtimes when patients were sitting in a chair and received timely assistance were evaluated.  **Cognitive outcomes**  Patients were observed systematically for two minutes before moving to the next room in continuous sequential observations between 8 am and 4 pm. The highest level of activity during each observation period was recorded along with physical activity, cognitive/social activity, and company. Percentage of observations where patient was standing or walking and the percentage where they were engaged in cognitive or social functioning (e.g., talking to others, reading, watching television) were evaluated. |
| Naylor et al. (2014)  USA | **Method**  Controlled trial. Prospective comparative effectiveness study  (Quasi-experimental designs)  **Objective**  To report the effects of three evidence-based interventions of varying intensity, each designed to improve outcomes of hospitalized cognitively impaired older adults.  **Sample**  202 older patients (Augmented Standard Care (ASC): 65; Resource Nurse Care (RNC): 71 and Transitional Care Model [TCM]: 66)  **Setting**  3 hospitals within an academic health system. | Education/training component was present only in Resource Nurse Care (RNC) or Transitional Care Model (TCM) interventions.  For RNC intervention, hospital employed RNs completed a web- based module developed by the study team in consultation with clinical experts prior to the intervention. The module focused on managing and transitioning hospitalized cognitively impaired older adults. They also attended seminars on issues relevant to cognitive impairment.  For TCM intervention, Advanced Practice Nurses (APNs) completed orientation designed for resource nurses, along with TCM specific web-based modules and individualized clinical experiences.  RNC intervention: Trained RNs coached other nurses involved in patient care. | **Level of intervention-** medium scale, 3 hospitals, units, model of care  **Care process changes**  One of the three interventions ASC, RNC and TCM were randomly assigned to one of three hospitals. All interventions included screening, reporting and documenting cognitive deficits.  RNC conducted cognitive screening within 24 hours of hospitalization. Findings were communicated to the interdisciplinary team within 2 hours.  TCM protocol consisted of hospital and home visits by advanced practice nurses (APN), physician follow-up, telephone outreach and supplemented care of hospitalized patients (not defined) and APNs substituted for RN home care. | **Functional outcomes**  Functional status (patient’s ability to conduct self-care [BADL] and instrumental activities of daily living [IADL], i.e., patient’s ability to perform higher **l**evel activities such as using the telephone, medication management, household chores) were collected by in-person interviews with caregivers. |
| Peyrusque et al. (2021)  Canada | **Method**  Cross-sectional study. Single arm interventional pragmatic pilot study  **Objective**  To assess the effect of a pragmatic, unsupervised, and specific PA program (SPRINT) on health care practice and functional capacities in hospitalized older patients  **Sample**  19 patients enrolled in SPRINT program  **Setting**  Geriatric Assessment Unit (GAU) | All GAU professionals (nurses, physicians and physiotherapists) received a 20-minute group training on SPRINT prior to study.  Note education previously described in Juneau et al. (2018). | **Level of intervention-** small scale, 1 hospital, 1-unit, single focus.  **Care process changes**  SPRINT included four exercise categories according to a level of mobility. The categories range from patient completing exercises alone to requiring professional supervision. Visual and verbal reminders to professionals and encouragement to participants and caregivers. Patients wore physical activity monitoring using accelerometers. | **Functional outcomes**  Ability of the patient to complete 7 activities of daily living (ADL) (eating, washing, dressing, grooming, urinary function, bowel function and toileting) assessed using the Functional Autonomy Measurement System (SMAF). Mobility was evaluated using the Physiotherapy Functional Mobile Profile (PFMP) tool. |
| Rodrigues et al. (2020).  Portugal | **Method**  2-group randomized controlled trial (RCT)  **Objective**  To test the hypothesis that a nursing care program focused on basic self-care (N_BSC) improves  functional outcomes in older patients admitted to an acute medical unit.  **Sample**  182 older adults (Intervention group: 91 and usual care group: 91)  **Setting**  580-bedded teaching hospital | All nurses and nursing assistants were educated on N_BSC. The N_BSC program was inspired by the Eat Walk Engage model and Function-focused Care Philosophy. Program focused on improving daily walking, trips to the toilet by walking (with support devices or with people support) and all daytime meals seated (out of bed). Education was provided prior to the intervention. | **Level of intervention-** medium scale, 1 hospital, 1 unit, model  **Care process changes**  N_BSC program involved changing physical structures of the service, reorganizing the nursing care, and coaching nurses and nursing assistants. Nurses encouraged and assisted patients to eat/walk or toilet use, ensure patients sitting ready to eat and promoted self-care, walk destinations, and minimized clinical activities during meal times.  **Environmental**  A 30-meter blue line along with handrail in the central corridor for walking support.  Ambulation pathways with distance markers. Hospital furniture rearranged to eliminate obstacles.  Social and dining room. Television, sofa, games, magazines/ newspapers available. 2 hand-held carts to carry portable oxygen cylinder.  **Administrative decisions** | **Functional outcomes**  **Functional status** (Bathing, dressing, eating, and toileting [BADL]) were assessed using Katz index.  Functional status of patients was assessed for 2 weeks prior to admission (based on self-report), at admission (observation based) and at discharge (nursing evaluation). |
| Rubin et al. (2011)  USA | **Method**  Not reported (? cohort study)  (Quasi-experimental designs)  **Objective**  To describe the evolution of the HELP program at Shadyside over the 7-year period from 2002–2008 (inclusive), including adaptations, patient outcomes, cost savings, challenges and successes.  **Sample**  Over 7000 Patients aged 70 and older who met the HELP criteria.  **Setting**  500 bed community teaching hospital | Volunteers received a complete training program. Some volunteers were trained by speech therapists in an enhanced feeding protocol, while some were trained by physical therapists in an enhanced mobilization protocol.  Measured staff knowledge at baseline and re-assessed after 6 months, implies that staff may have received training/education)  Volunteer trainings were fully standardized and well-documented.  Note education previously described in Inouye et al. (1993a & 2000). | **Level of intervention-** medium scale, 1 hospital, 6 units, model  **Care process changes**  HELP model included staffing, operational structure, interventions, and quality assurance. The care processes from Inouye et al. 2000 & 2006. Sleep protocol was modified to include hand rather than back massage.  **Environmental**  Unit-wide noise reduction strategies were already being implemented prior to HELP.  **Administrative decisions**  Hospital leaders agreed to continuously fund the HELP and allowed the HELP program to expand to additional units.  **Facilitation**  Weekly meetings were held with Hospital Elder Life Program Staff and monthly meetings with the program director and a process improvement specialist.  Elder Life Specialist was designated as the ‘lead volunteer coordinator’ to provide front-line supervision and coordination to volunteers.  A bachelor-prepared nurse was enrolled so as to allow nurse specialist to extend her scope. | **Cognitive outcomes**  Delirium assessed by Confusion Assessment Method. |
| Suwanpasu et al. (2015)  Thailand | **Method**  A pre-post test design without a control group  (Quasi-experimental designs)  **Objective**  To assess the effectiveness of a geriatric resource nurse (GRN) programme in maintaining the functional status of elderly  patients hospitalized for community-acquired pneumonia (CAP).  **Sample**  15 men and 8 women (N=23) with CAP and admitted to the hospital between July 2014 and December 2014.  **Setting**  King Chulalongkorn Memorial hospital | The registered nurses (RNs) who volunteered to become GRNs, had worked with elderly patients for at least 5 years and possessed the knowledge, skills, and ability to improve care for hospitalized elders. GRNs were trained to be the frontline experts to assess elderly patients and to consult with a geriatric advanced practice nurse with expertise in handling more complex cases involving elders at high risk.  Geriatric core curriculum included contents such as: geriatric syndromes, geriatric specific disease entities, multidimensional assessment of the older adult (SPICCIES tool), and best practice protocol. SPICCIES assessed sleep disorder, problem with eating or feeding, incontinence, confusion, caregiver preparedness, immobility, evidence of falls, and skin breakdown. | **Level of intervention-** medium scale, 1 hospital,  1 unit, model  **Care process changes**  Used Nurses Improving Care for Health System Elders (NICHE) model to train geriatric resource nurses. Focused on geriatric syndromes, using the geriatric core curriculum protocols. Unit-based clinical rounds with a geriatric advanced practice nurse were held twice a week. Multidisciplinary advisory team including physician, nursing administrator, physiotherapist, and pharmacist was formed.  **Facilitation**  Geriatric advanced practice nurses supported the GRNs to distinguish risk factors and to make recommendations, physical assessment skills, and to encourage communication between health care teams, patients and families.  GRNs were also provided with relevant journal articles as support for recommendations.  GRNS were continuously mentored to improve their skills. | **Functional outcomes**  The SPICES tool was used to assess patients’ sleep disorder, problems with eating or feeding, incontinence, confusion, immobility, evidence of falls, and skin breakdown.  Sleep disorder assessed using the Verran and Synder-Halpern Sleep Scale.  Problems with eating or feeding are evaluated using the Edinburgh Feeding Evaluation in Dementia Questionnaire.  The immobility assessment tool is based on the NANDA statement: “immobility is the individual experiencing a limitation of ability for independent physical movement”.  Evidence of falls evaluated using the Hendrich II Fall Risk Model. Skin breakdown is evaluated using the Braden Scale for Predicting Pressure Sore Risk.  **Cognitive outcomes**  Confusion is assessed using the Thai version of the Confusion Method Assessment algorithm, based on the Diagnostic and Statistical Manual of Mental Disorders criteria for delirium. |
| Vidan et al. (2009)  Spain | **Method**  Prospective controlled clinical trial  (Quasi-experimental designs)  **Objective**  To analyze the effectiveness of a multicomponent  intervention integrated into daily practice for the  prevention of in-hospital delirium in elderly patients.  **Sample**  542 patients were included (170 patients in the GI [Geriatric Unit] group and 372 in the UC [Usual Care] group)  **Setting**  University hospital | Briefly mentioned that education was targeted to staff to change the approach of patient care, but not discussed in detail.  Educational session explaining the characteristics of delirium, recognition of delirium and risk factors were given by senior geriatrician and specialist geriatric nurse.  Poster with environmental and general prevention measures such as avoiding noise, stimulating mobilization and hydration, advice about speaking to the patient were kept in nurses’ station.  Nurses used cards with recommendations included in the first page of the treatment book. | **Level of intervention-** medium scale, 1 hospital, 1 unit, model  **Care process changes**  Intervention included staff education and specific targeted delirium risk factors – hydration and nutrition, orientation, sensory perception (glasses and hearing aids), sleep preservation, mobilization, and a drug list review.  **Environmental**  Clocks and calendars in every room.  **Administrative decisions**  Avoid physical restraints. Used only with medical authorization and signature of physician.  **Facilitation**  Geriatrician and Specialist Geriatric Nurse | **Functional outcomes**  Functional decline calculated as the proportion of participants whose performance on activities of daily living (ADLs) was worse at discharge than at baseline. ADLs assessed by Katz index of activities of daily living. The ability to walk was defined according to the Functional Ambulation Classification.  **Cognitive outcomes**  Cognitive function assessed by Spanish version of the Folstein Mini-Mental State Examination (MMSE). Delirium assessed by Confusion Assessment Method. |
| von Renteln-Kruse & Krause (2007).  Germany. | **Method**  Prospective cohort with historical control study.  (Quasi-experimental designs)  **Objective**  To evaluate the  effect of an interdisciplinary team approach on reducing the  number of falls in geriatric hospital inpatients by comparing  long periods before and after introduction of the intervention.  **Sample**  Before intervention: 4,272 patients and After intervention: 2,982 patients.  **Setting**  Geriatric clinic of an academic teaching hospital | Nurses, therapeutic staff, and physicians were given an hour-long presentation fall prevention measures. Participants were trained in supporting patient transfer, fall-risk assessment tool and the checklists to be used on the wards.  Patients at risk and their relatives/caregivers were provided individual education about fall risk and preventive measures, including behavioral changes.  Four half-day training seminars and refresher courses on patient handling were held per year.  Other routinely available measures were: training in ADLs, balance and gait, transfer, safe use of devices (occupational therapists, nurses). | **Level of intervention-** small scale, 1 hospital,  1 unit, single focus  **Care process change**  Fall prevention included  assessment for fall-risk within 48 hours of admission. If a risk, a visible ‘risk alert’ sign was placed above the patient’s bed.  5-page flyer for patients and families about typical risks and in hospital preventative measures (footwear, eyeglasses, hearing aids, hip protectors and mobility devices) instructions on getting out of bed, fluid intake and communications.  Patients in need of frequent toileting were provided.  Patients were encouraged to get out of bed as early as possible and to sit on normal chairs when eating meals.  additional assistance or supervision.  **Environmental**  A visible ‘risk alert’ sign was placed above the patient’s bed if they were identified at risk on assessment. Mobility devices were provided immediately upon admission if it was determined necessary. Commodes were provided | **Functional outcome**s  Activities of daily living were assessed using the Barthel Index.  Fall risk was assessed using the St. Thomas’s Risk Assessment Tool in Falling Elderly Inpatients (STRATIFY). |
| Wand et al. (2014).  Australia | **Method**  A before and after study  (Quasi-experimental designs)  **Objective**  To evaluate the effectiveness of a multifaceted educational program in preventing delirium in hospitalized older patients and improving staff practice, knowledge and confidence.  **Sample**  255 patients of age 65 years and older (126: pre-intervention and 129: post-intervention)  77 staff (45 prior to the educational intervention and 32 after intervention).  35 were nurses and 39 doctors.  **Setting**  22-bed general medical ward of a district hospital | One-hour education sessions were organized as part of the routine education program for Nursing and medical staff. Nurse educator conducted education sessions for nurses and psychogeriatrician and geriatrician conducted sessions for medical staff.  Educational content included general information on delirium (definition, risk factors for developing delirium, symptoms, epidemiology, differential diagnosis and outcomes), prevention (detailed discussion of how to address potentially modifiable risk factors), and management of patients with delirium (early detection, legal framework of care, non-pharmacological and pharmacological management).  Weekly tutorials were provided on the study ward and one-page summary of the key points of each education session. Posters were displayed in prominent areas of the study ward reinforcing these key points. | **Level of intervention-** medium scale, 1 hospital,  1 unit, model  **Care process changes**  Interventions included addressing bladder and bowel function (constipation, urinary retention), hydration, pain, nutrition, sensory aides and communication, remove attachments (catheters, IVs, and catheters), oxygenation, sleep, medication review, management of drug dependence.  **Environmental**  Ward environment was optimized to make it elder friendly. Examples include large-face clocks in patient rooms and large picture-based signs indicating toilets.  **Administrative decisions**  Avoid use of physical restraints.  **Facilitation**  Local champions for delirium prevention were identified as the delirium resource staff, a Nurse Educator and senior geriatrician (WT)  Weekly tutorials with these delirium resource staff. | **Functional outcomes**  Overall functioning measured by the Barthel activities of daily living index.  **Cognitive outcomes**  Cognitive impairment assessed using Mini-Mental Status examination.  Dementia assessed using the Blessed dementia scale and clock-draw test.  Delirium assessed by confusion assessment method.  Barthel’s score from admission to discharge in the post intervention group. |
| Wang et al. (2020).  China | **Method**  2-arm parallel-group, single-blind, cluster randomized clinical trial  (RCT)  **Objective**  To investigate the effectiveness of the Tailored, Family-Involved Hospital Elder  Life Program (t-HELP) for preventing postoperative delirium (POD) and functional decline in older patients after a  noncardiac surgical procedure.  **Sample**  281 patients (Intervention/t-help group: 152 and usual care group/control group: 129)  **Setting**  24 nursing units across 6 surgical floors of hospital | Project director provided intensive training, including theoretical knowledge and assessment skills for delirium to all the assessors. Training included a review of the t-HELP procedure outlined in a short booklet and video.  Medical postgraduates, majoring in geriatrics or geriatric nursing were trained as coordinators, who took charge of delivering the intervention plan to nurses, surgical doctors and other team staff. | **Level of intervention-** medium scale, 1 hospital, 6-unit model  **Care process changes**  The interdisciplinary t-HELP (tailored for family involvement instead of volunteers from the hospital elder life program adapted from the HELP program) Intervention included 3 universal protocols and 8 targeted protocols. Universal protocols included orientation, therapeutic activities, and early mobilization. Targeted protocols were implemented according to the presence of delirium-related risk factors and included pain management, sleep enhancement, nutritional assessment, fluid repletion/constipation management, etc. Control patients in a usual care unit received the usual treatment and care, without individualized assessment or interdisciplinary intervention.  Nurses screened for delirium risk factors. Geriatric specialists develop individualized intervention plan based on delirium risk factors. Intervention daily after surgery. Patients provided with information booklets with information about interdisciplinary interventions, pain alleviation, sleep improvement, and relaxation skills.  **Facilitation**  Coordinators assisted nurses organizing the interdisciplinary consultation twice a week to discuss special cases | **Functional outcomes**  Changes in physical function before and after the surgical procedures measured using the Barthel index and instrumental activities of daily living (IADLs).  **Cognitive outcomes**  Postoperative delirium assessed using CAM (Confusion Assessment Method)  Incidence of severe delirium during hospitalization was measured by the Chinese version of MDAS (Memorial Delirium Assessment Scale)  Change in cognitive function was assessed by the Chinese version of the SPMSQ (Short Portable Mental Status Questionnaire). |
| Wanich et al. (1992)  USA | **Method**  Quasi-experimental design  **Objective**  To examine the effectiveness of a nursing intervention for elderly  hospitalized patients as measured by functional outcomes  **Sample**  235 hospitalized patients (135 in intervention group and 100 in control group) aged 70 years and older, admitted between Sunday noon and Friday noon.  **Setting**  700-bed urban, teaching hospital affiliated with schools of nursing and medicine. | Two geriatric clinical specialists gave an in-service program to nursing staff that included mental and functional status assessments, gerontological nursing management of deficits in sensory-perceptual function, mobility and environmental modifications. Educational sessions were repeated once during the course of the study. | **Level of intervention-** medium scale, 1 hospital, 1 unit, model  **Care process changes**  Nurse specialists provided orientation cues to patients (e.g. day of the week, current events, a discussion of their condition and information about upcoming diagnostic or therapeutic measures).  Families and nursing staff were taught to communicate clearly and slowly with patients and use repetition and orientation cues.  Patients mobilized by nursing staff out of bed each day, ambulated, taken out of their rooms and consulted physical and occupational therapy.  Families were asked to visit frequently or call daily; bring in photos and personal mementos from home to assist in orientation and personalizing the environment. Patients' medications reviewed daily. Medications suspected as contributing to delirium evaluated critically on an individual basis, use discouraged. Families asked to make glasses and hearing aids available and nursing staff encouraged to use them. Patients and families asked about preferences (favorite television programs, news channels and radio stations) to assist with stimulation and orientation.  **Environmental**  Environmental modifications were done to meet the needs of hearing or vision impaired patients (details not given). Updated calendars were placed in every room. Lighting was used to decrease sensory deprivation. Night lights were turned on at dusk and left on until morning.  **Facilitation**  Nurse specialists held frequent informal discussions with the  staff.  **Discharge planning/transition care**  A Geriatric Clinical Nurse Specialist (GCS) reviewed patient’s functional and cognitive status weekly with the discharge planning team, including primary nurse, a social worker, a discharge planning nurse, a physical and occupational therapist and a nutritionist. | **Functional outcomes**  Functional status assessed by Katz Index of Activities of Daily Living.  **Cognitive outcomes**  Cognitive deficits assessed by Mini-Mental State Examination.  Delirium diagnosed by a study psychiatrist based on DSM-III (Diagnostic & Statistical Manual, third edition) criteria. |
| Zisberg et al. (2018).  Israel | **Method**  Prospective cohort study  (Quasi-experimental designs)  **Objective**  To demonstrate the process of adapting a human factors framework, the Systems Engineering  Initiative for Patient Safety (SEIPS 2.0)], as a guided model to articulate a site-specific, culturally based intervention to improve in-hospital mobility in older adults.  **Sample**  203 older adults hospitalized in two internal medicine units at an academic medical center.  116 medical staff, including nurses, nurse’s aides (NAs), physical therapists (PTs), and medical doctors (MDs)  **Setting**  Academic medical center | Staff were provided online tutorials and face to face training by PTs to enhance their knowledge. | **Level of intervention-** small scale, 1 hospital,  2 units, single focus  **Care process changes**  The Systems Engineering Initiative for Patient Safety (SEIPS) 2.0 model, development of WALKFFOR model.  Interdisciplinary protocols for mobility  Bilingual patient/family resources (brochures, posters) on maintaining mobility.  **Environmental**  Walking trails,  **Administrative decisions**  Extra walkers purchased  Electronic reports on mobility, mandatory reporting on mobility  **Facilitation**  Head nurse led team meetings to discuss interventions, workflow.  Daily review of EMRs, weekly discussion of protocol implementation. | **Functional outcomes**  In-hospital mobility (900 steps and above considered as mobility level). |
