# Supplementary material for: Interventions supporting the translation of gerontological evidence into practice to optimize functional outcomes for hospitalized older adults: A scoping review
Source: PLoS One. 2025 Jun 16;20(6):e0324953. doi: 10.1371/journal.pone.0324953 (PMC12169582; doi:10.1371/journal.pone.0324953)
Supplement: S1 Fig — (DOCX) [file pone.0324953.s005.docx]

**S-1 Fgure.**

*Preferred Reporting Items for Systematic Reviews and Meta-Analyses (PRISMA) Flow Diagram*

References from other sources **(n = 0)**

Citation searching (n =0 )

Grey literature (n =0 )

**Identification**

Studies screened **(n = 4781)**

Duplicates removed **(n = 3921)**

Studies excluded as irrelevant **(n = 4482)**

Studies sought for retrieval **(n = 299)**

Studies not retrieved **(n = 0)**

Studies assessed for eligibility **(n = 299)**

Studies excluded **(n = 262)**

Wrong publication (n=43)

Wrong study design (n=43)

Wrong setting (n=11)

Wrong population (n=7)

Wrong outcomes (n=18)

No education component (n=113)

Duplicate (n = 6)

Unable to access full text (n=21)

Studies from databases/registers **(n = 8702)**

**Screening**

Studies included **(n = 37)**

**Included**

Identified from hand search of reference lists.

**(n = 1)**

Total studies included in review **(n = 38)**
