# Supplementary material for: Interventions supporting the translation of gerontological evidence into practice to optimize functional outcomes for hospitalized older adults: A scoping review
Source: PLoS One. 2025 Jun 16;20(6):e0324953. doi: 10.1371/journal.pone.0324953 (PMC12169582; doi:10.1371/journal.pone.0324953)
Supplement: Attachment — Submitted filename: PONE-D-24-35120 reviewed SD122024 (1).docx [file pone.0324953.s006.docx]

PLOS ONE

Interventions Supporting Implementation of Gerontological Education for Healthcare

Professionals to Improve Functional Outcomes for Hospitalized Older Adults: A

Scoping Review

--Manuscript Draft--

| Manuscript Number: | PONE-D-24-35120 |
| --- | --- |
| Article Type: | Research Article |
| Full Title: | Interventions Supporting Implementation of Gerontological Education for Healthcare Professionals to Improve Functional Outcomes for Hospitalized Older Adults: A Scoping Review |
| Short Title: | Interventions Supporting Implementation of Gerontological Education: A Scoping Review |
| Corresponding Author: | Kathleen Frances Hunter, PhD  University of Alberta  Edmonton, Alberta CANADA |
| Keywords: | older adults; hospitals; Education; function |
| Abstract: | Background and Objectives. Hospitalized older adults are at risk for both physical and cognitive functional decline. To support the implementation of interventions optimizing their function, both healthcare professional knowledge and other factors that may impact practice change should be addressed. The objective of this study was to examine interventions that have been used to support the implementation of gerontological knowledge by hospital-based healthcare professionals and/or  interprofessional teams to improve older adults’ functioning.  Materials and Methods. This scoping review used the method and steps recommended by Peters et al. Target studies examined initiatives or care delivery models to improve functional outcomes for adults aged 65+ in hospital settings. Included studies described an educational intervention for healthcare professionals as part of an initiative to promote practice change and assessed functional outcomes (physical and/or cognitive).  Results. Thirty-eight studies were deemed eligible. Numerous interventions to support implementation of knowledge to improve functional outcomes were identified. The interventions included care processes, environment, administrative support, facilitation/leadership, and discharge/transition planning. Studies employed a range of measures to assess functional outcomes in hospitalized older adults.  Discussion. Older studies tended to focus on nurse-driven interventions, and newer ones on interprofessional interventions. Gaps in the literature are highlighted in terms of theory, research designs, intervention descriptions, country representation, policy, and environmental changes. Future systematic reviews on interventions to enhance older adults’ functioning should examine intervention effectiveness and compare single- and multi-intervention programs. Scholars should then develop strategies based on implementation science theory to ensure successful implementation of effective interventions, while taking into consideration the realities of hospital units, such as short staffing, resource constraints, and overcrowding. |
| Order of Authors: | Kathleen Frances Hunter, PhD |
|  | Jeffrey I. Butler |
|  | Shovana Shrestha |
|  | Adrian Wagg |
|  | Nick Millar |
|  | Frances Carr |
|  | Sherry Dahlke |
|  | C. Allyson Jones |
| Additional Information: |  |

| Question | Response |
| --- | --- |
| Financial Disclosure  Enter a financial disclosure statement that describes the sources of funding for the work included in this submission. Review the [submission guidelines](http://journals.plos.org/plosone/s/submission-guidelines#loc-financial-disclosure-statement) for detailed requirements. View published research articles from [PLOS ONE](https://journals.plos.org/plosone/) for specific examples.  This statement is required for submission and will appear in the published article if the submission is accepted. Please make sure it is accurate. | Yes |
| Funded studies Enter a statement with the following details: - Initials of the authors who received each award - Grant numbers awarded to each author - The full name of each funder - URL of each funder website - Did the sponsors or funders play any role in the study design, data collection and analysis, decision to publish, or preparation of the manuscript? |  |
| Did you receive funding for this work? |  |
| Please add funding details.   as follow-up to "Financial Disclosure  Enter a financial disclosure statement that describes the sources of funding for the work included in this submission. Review the [submission guidelines](http://journals.plos.org/plosone/s/submission-guidelines#loc-financial-disclosure-statement) for detailed requirements. View published research articles from [PLOS ONE](https://journals.plos.org/plosone/) for specific examples.  This statement is required for submission and will appear in the published article if the submission is accepted. Please make sure it is accurate. | KFH was awarded a Knowledge Synthesis Grant from the Seniors Health Strategic  Clinical Network™ (SCN), Alberta Health Services (no grant # assigned). Alberta Health Services did not play any role in the the study design, data collection and analysis, decision to publish, or preparation of the manuscript. https://www.albertahealthservices.ca/research/Page14021.aspx |

| Funded studies Enter a statement with the following details: - Initials of the authors who received each award - Grant numbers awarded to each author - The full name of each funder - URL of each funder website - Did the sponsors or funders play any role in the study design, data collection and analysis, decision to publish, or preparation of the manuscript? |  |
| --- | --- |
| Did you receive funding for this work?" |  |
| Please select the country of your main research funder (please select carefully as in some cases this is used in fee calculation).   as follow-up to "Financial Disclosure  Enter a financial disclosure statement that describes the sources of funding for the work included in this submission. Review the [submission guidelines](http://journals.plos.org/plosone/s/submission-guidelines#loc-financial-disclosure-statement) for detailed requirements. View published research articles from [PLOS ONE](https://journals.plos.org/plosone/) for specific examples.  This statement is required for submission and will appear in the published article if the submission is accepted. Please make sure it is accurate. | CANADA - CA |
| Funded studies Enter a statement with the following details: - Initials of the authors who received each award - Grant numbers awarded to each author - The full name of each funder - URL of each funder website - Did the sponsors or funders play any role in the study design, data collection and analysis, decision to publish, or preparation of the manuscript? |  |
| Did you receive funding for this work?" |  |
| Competing Interests | The authors have declared that no competing interests exist. |

| Use the instructions below to enter a competing interest statement for this submission. On behalf of all authors, disclose any [competing interests](http://journals.plos.org/plosone/s/competing-interests) that could be perceived to bias this work—acknowledging all financial support and any other relevant financial or nonfinancial competing interests.  This statement is required for submission and will appear in the published article if the submission is accepted. Please make sure it is accurate and that any funding sources listed in your Funding Information later in the submission form are also declared in your Financial Disclosure statement.  View published research articles from [PLOS ONE](http://journals.plos.org/plosone/) for specific examples. |  |
| --- | --- |
| NO authors have competing interests Enter: The authors have declared that no competing interests exist. Authors with competing interests Enter competing interest details beginning with this statement: I have read the journal's policy and the authors of this manuscript have the following competing interests: [insert competing interests here] |  |
| * typeset |  |
| Ethics Statement  Enter an ethics statement for this submission. This statement is required if the study involved:   - Human participants - Human specimens or tissue - Vertebrate animals or cephalopods - Vertebrate embryos or tissues | N/A. |

| • Field research  Write "N/A" if the submission does not require an ethics statement.  General guidance is provided below. Consult the [submission guidelines](http://journals.plos.org/plosone/s/submission-guidelines#loc-guidelines-for-specific-study-types) for detailed instructions. Make sure that all information entered here is included in the Methods section of the manuscript. |  |
| --- | --- |

Format for specific study types

Human Subject Research (involving human

participants and/or tissue)

Give the name of the institutional review

board or ethics committee that approved the

study

•

Include the approval number and/or a

statement indicating approval of this

research

•

Indicate the form of consent obtained

(

written/oral) or the reason that consent was

not obtained (e.g. the data were analyzed

anonymously)

•

Animal Research (involving vertebrate

animals, embryos or tissues)

Provide the name of the Institutional Animal

Care and Use Committee (IACUC) or other

relevant ethics board that reviewed the

study protocol, and indicate whether they

approved this research or granted a formal

waiver of ethical approval

•

Include an approval number if one was

obtained

•

If the study involved

non-human primates

,

add

additional details

about animal welfare

and steps taken to ameliorate suffering

•

If anesthesia, euthanasia, or any kind of

animal sacrifice is part of the study, include

briefly which substances and/or methods

were applied

•

Field Research

Include the following details if this study

involves the collection of plant, animal, or

other materials from a natural setting:

Field permit number

•

Name of the institution or relevant body that

granted permission

•

Data Availability

Authors are required to make all data

underlying the findings described fully

available, without restriction, and from the

time of publication. PLOS allows rare

exceptions to address legal and ethical

concerns. See the

[PLOS Data Polic](http://journals.plos.org/plosone/s/data-availability)

[y](http://journals.plos.org/plosone/s/data-availability)

and

[FA](http://journals.plos.org/plosone/s/data-availability#loc-faqs-for-data-policy)

[Q](http://journals.plos.org/plosone/s/data-availability#loc-faqs-for-data-policy)

for detailed information.

Yes - all data are fully available without restriction

| A Data Availability Statement describing where the data can be found is required at submission. Your answers to this question constitute the Data Availability Statement and will be published in the article, if accepted. |  |
| --- | --- |
| Important: Stating ‘data available on request from the author’ is not sufficient. If your data are only available upon request, select ‘No’ for the first question and explain your exceptional situation in the text box. |  |
| Do the authors confirm that all data underlying the findings described in their manuscript are fully available without restriction? |  |
| Describe where the data may be found in full sentences. If you are copying our sample text, replace any instances of XXX with the appropriate details.   - If the data are held or will be held in a public repository, include URLs, accession numbers or DOIs. If this information will only be available after acceptance, indicate this by ticking the box below. For example: All XXX files   are available from the XXX database (accession number(s) XXX, XXX.).   - If the data are all contained within the manuscript and/or Supporting Information files, enter the following: All relevant data are within the manuscript and its Supporting Information files. - If neither of these applies but you are able to provide details of access elsewhere, with or without limitations, please do so. For example:   Data cannot be shared publicly because of [XXX]. Data are available from the XXX Institutional Data Access / Ethics Committee (contact via XXX) for researchers who meet the criteria for access to confidential data.  The data underlying the results presented in the study are available from (include the name of the third party | All relevant data are within the manuscript and its Supporting Information files. |

| and contact information or URL).  • This text is appropriate if the data are owned by a third party and authors do not have permission to share the data.  * typeset |  |
| --- | --- |
| Additional data availability information: |  |

Manuscript	[Click here to access/download;Manuscript;BADL_Scoping](https://www2.cloud.editorialmanager.com/pone/download.aspx?id=37339732&guid=e337d037-9fbc-476e-b0ea-6aeadbd3a3a9&scheme=1)

[review_manuscript 5_ FINAL_23_08_2024.docx](https://www2.cloud.editorialmanager.com/pone/download.aspx?id=37339732&guid=e337d037-9fbc-476e-b0ea-6aeadbd3a3a9&scheme=1)

1

2 **Interventions Supporting Implementation of Gerontological Education for Healthcare**
3 **Professionals to Improve Functional Outcomes for Hospitalized Older Adults: A Scoping**

# 4 Review

5

6 *Kathleen F. Hunter^1^, Jeffrey I. Butler^1^, Shovana Shrestha^1^, C. Allyson Jones^2^, Adrian Wagg^3^,
7 Nick Millar^1^, Frances Carr^3^, Sherry Dahlke^1^

8

9 ^1^Faculty of Nursing, University of Alberta, Edmonton, Alberta, Canada
10 ^2^Department of Physical Therapy, University of Alberta, Edmonton, Alberta, Canada
11^3^Division of Geriatric Medicine, Department of Medicine, University of Alberta, Canada

12

13

14

15

16 * Corresponding author
17 Email: kathleen.hunter@ualberta.ca (KFH)

18

19

20

21

22

23

24

25

# Abstract

**Background and Objectives.** Hospitalized older adults are at risk for both physical and cognitive functional decline. To support the implementation of interventions optimizing their function, both healthcare professional knowledge and other factors that may impact practice change should be addressed. The objective of this study was to examine the characteristics/types of interventions that have been used to support the implementation of gerontological knowledge by hospital-based healthcare professionals and/or interprofessional teams to improve older adults’ functioning. **Materials and Methods.** This scoping review used the method and steps recommended by Peters et al. ~~Target studies examined initiatives or care delivery models to improve functional outcomes for adults aged 65+ in hospital settings.~~ Included studies described an educational intervention for healthcare professionals as part of a wider initiative to promote practice change and assessed functional outcomes (physical and/or cognitive).

**Results.** Thirty-eight studies were deemed eligible. Numerous interventions to support implementation of knowledge to improve functional outcomes were identified. The interventions included in addition to educational strategies, care processes, environmental changes, administrative support, facilitation/leadership roles, and discharge/transition planning. Studies employed a range of measures to assess functional outcomes in hospitalized older adults.

**Discussion.** Older studies tended to focus on nurse-driven interventions, and newer ones on interprofessional interventions. Gaps in the literature are highlighted in terms of theory, research designs, intervention descriptions, country representation, policy, and environmental changes. Future systematic reviews on interventions to enhance older adults’ functioning should examine intervention effectiveness and compare single- and multi-intervention programs. Scholars should then develop strategies based on implementation science theory to ensure successful

49 implementation of effective interventions, while taking into consideration the realities of hospital
50 units, such as short staffing, resource constraints, and overcrowding.
51 *Keywords:* older adults, hospitals, education, function

52

53

# Introduction

Older adults in hospital are at risk for both physical and cognitive decline.^1-3^ Many older adults have highly complex care needs and entering the hospital system, which prioritizes acute treatment and system efficiency,^4^ can result in functional losses and poor outcomes.^5, 6^ Factors including limited mobility and suboptimal continence care are associated with functional decline in hospital and can contribute to further decline post-discharge.^6, 7^ Consequently, optimizing functioning for this patient population should be a health system priority. To address this concern, interventions impacting practice change should be addressed along with healthcare professionals’ knowledge.^8^ Informed by ecological concepts from implementation science,^9^ this scoping review sought to examine interventions to support implementation of knowledge from gerontological education for hospital-based healthcare professionals and/or interprofessional teams to improve older adults’ functioning.

To maintain function and limit preventable hospital-acquired functional loss, there is a need to shift the focus of care for hospitalized older people to the preservation and restoration of function.^10, 11^ For example, evidence suggests that engaging hospitalized older persons in physical activity^12^ and basic activities of daily living (e.g., toileting, dressing)^13^ may prevent both physical and cognitive decline. The implementation of function-oriented approaches, however, has been hampered by inadequate gerontological knowledge on the part of healthcare professionals.^14-17^ Yet, education alone does not change practice and not all gerontological education initiatives will spur a transformation in older adults’ care^18^; a wide variety of factors influencing knowledge uptake (e.g., dedicated champions, strong communication, effective teamwork, and appropriate resources) must be accounted for.^19, 20^ Consequently, we conceptualize healthcare professional education as necessary to catalyze a shift to more functionoriented approaches, but as insufficient in isolation. It is thus essential to develop a broader understanding of other interventions that have been implemented in acute hospital settings to support the application of new knowledge to optimize older adults’ functioning.

Previous studies have underscored that, in acute care settings, numerous factors shape healthcare professionals’ perceptions of the feasibility of implementing new approaches to care.^21^ However, it remains unknown what interventions have been employed, in addition to healthcare professional education, to promote function-oriented care for acutely hospitalized older adults.

# Objectives

Our aim was to determine what interventions for hospital-based healthcare professionals and/or interprofessional teams, in addition to gerontological education, have been implemented to optimize older adults’ functional outcomes in hospital settings. Accordingly, we mapped the evidence on educational interventions for healthcare professionals that have been implemented as part of an initiative to drive practice change.

# Materials and Methods

We used the guidelines for scoping reviews recommended by Peters et al.^22^ Our project sought to understand how healthcare professionals’ gerontological knowledge can be improved and applied, one of the research priorities identified in a provincial priority-setting partnership.^23^ As we could not locate any existing review in the hospital setting, we determined that a broader scoping review to map the breadth of the available evidence (as opposed to a more focused systematic review) was most appropriate.^24, 25^ According to Munn et al., (2022), scoping reviews are “a type of evidence synthesis that aim to systematically identify and map the breadth of evidence available on a particular topic, field, concept, or issue, often irrespective of source (i.e., primary research, reviews, non-empirical evidence) within or across particular contexts” (page 1).^25^ Using this approach, we formulated the research question; identified relevant studies; selected studies; charted/extracted data; and collated, summarized, and reported results. The

PRISMA-ScR (Preferred Reporting Items for Systematic Reviews and Meta-Analyses:

Extension for Scoping Reviews) guided our reporting (supplementary table 1).^22, 26^

# Inclusion/Exclusion Criteria

We included studies of any primary research design, with no restrictions on language/year of publication. Studies focused on initiatives or care delivery models to improve functional outcomes for adults aged 65+ in acute care hospital settings. Eligible studies: described educational interventions for healthcare professionals as a part of an initiative to promote practice change (e.g., care process change, environmental modifications), assessed functional outcomes in older adults (physical or cognitive), and could address single or multiple interventions. We excluded reviews and editorials, non-hospital studies, studies focused on adults less than 65 years old, and studies that did not describe an educational intervention component (see supplementary table 3).

# Search Strategy

An academic health sciences librarian (XX) developed the search strategy and conducted the search for relevant published studies. Searches were conducted in 2021 and updated in 2023.

Search terms included a combination of natural language vocabulary and controlled terms (subject headings), where available. Natural language terms were derived from two main concepts: 1) older hospitalized patients, and 2) Basic Activities of Daily Living (BADL) interventions, including interventions addressing specific elements such as mobility, ambulation, and personal hygiene. Search terms for BADL also included language for self-care, functional status, and functional decline (see Appendix A for search strategies for MEDLINE). The search focused on physical function and BADL, but both physical and cognitive outcomes were abstracted from any included studies. We conducted the search in six electronic databases: MEDLINE via OVID (1946 - Present); EMBASE via OVID (1974 - Present); Cumulative Index for Nursing and Allied Health Literature (CINAHL) via EBSCOhost (1946 - Present); Scopus via Elsevier (1972 - Present); Cochrane Library via Wiley (1992 - Present); TRIP Pro Medical Database (1997 - Present). We additionally conducted hand searches of the reference lists of included studies.

# Study Selection

Identified studies were uploaded to Covidence to support knowledge synthesis. After removal of duplicates, research team member pairs (XX and XX; XX and XX) independently screened the abstracts. The full text versions of potentially relevant studies were reviewed as pairs and any discrepancies were resolved through discussion and involvement of a third reviewer (XX), if consensus could not be achieved through discussion. Interrater agreement was not evaluated but regular consensus meetings were held for abstract screening.

# Data Extraction

Research assistants (XX and XX) conducted data extraction, which was verified by the principal investigator (XX) and research coordinator (XX). We extracted the following elements: (a) study characteristics: author, year, country; (b) study objectives, design, setting, sample; (c) education component(s), contextual component(s) (e.g., level of intervention, care/team process, facilitation) and functional outcomes (see Table 1).

# Data Analysis

We employed an inductive approach that facilitated identification of unanticipated patterns in the data, as well as an ecological lens that allowed us to differentiate between micro, meso, and macro levels of healthcare practice change.^9, 27^ A descriptive numerical summary of the included studies (overall number of studies included, types of study designs, year of publications, countries where studies were conducted, and study settings) and narrative summary of how review findings address the study aim and research question were developed,^22^ and key findings and gaps in the research evidence were identified. We developed preliminary categories, which were iteratively refined as analysis progressed, to provide a descriptive summary. We did not re-frame the study findings beyond what was reported in the original studies. Given that this was a scoping review (which should not address more targeted questions of intervention feasibility or effectiveness),^28^ we used our descriptive summary to map the educational interventions/practice change initiatives, their purposes, as well as the outcomes examined and how they were measured.

# Results

Database searches located 8,702 studies, 299 of which were included in full-text screening – 38 were ultimately deemed eligible (see PRISMA Flow Diagram in supplementary material).^26^ Included studies: were conducted between 1993 and 2023; 29 were non-randomized studies with a control;^29-57^ five were randomized controlled trials (RCTs)^58-62^; three were mixed-

method studies,^63-65^ and one was a quantitative descriptive study.^66^ Most studies were conducted in the United States (n=10), Australia (n=6), Canada (n=4) and Israel (n=3), two in Italy,

Netherlands, UK, Germany, and Spain, and one in Brazil, Belgium, Portugal, Thailand, and China. The sample size of the included studies ranged from 19 to 12,490 older adults (see Table

1 for a description of the included studies).

# Geriatric/Gerontological Education Initiatives

More than half the studies (n=23) reported a single educational initiative;^29, 42, 43^ ^33, 58, 56,^ 44, 34, 35, 66, 64, 63, 48, 49, 36, 57, 39, 60, 51, 53, 41, 62, 54, while the remaining studies reported two or more

educational initiatives.30, 31, 32, 46, 35, 46, 50, 38, 59, 37, 61, 40, 52, 55, 65

The educational initiatives were described as training (n=22 studies), education session (n=12), lecture/tutorial (n=5), in-service (n=4), poster (n=4), education module (n=3), as well as workshop, on the job coaching/training, presentation, pocket cards, resources, and material (n=2), and seminar, booklet, videos, handout, quiz, case vignette (n=1) (see Table 2 for frequency of educational strategies reported). Half the studies targeted a single clinical outcome

(n=19),43, 33, 58, 56, 44, 45, 66, 64, 63, 48, 49, 37, 51, 40, 52, 55, 62, 54, 65 and half (n=19) addressed multiple

clinical outcomes.29-32, 34, 42, 34, 41, 46, 35, 47, 36, 50, 57, 38, 39, 53, 59-61, 53 Most single-outcome education focused on mobility (n=11), followed by delirium (n=7) and cognitive deficits (n=1). Studies focusing on multiple clinical outcomes examined diverse topics such as: dementia, communication with patients, falls, and incontinence. Mobility and delirium were common clinical outcomes in studies targeting multiple outcomes (see Table 1). Education targeted nurses

(n=18)42, 43, 33, 58, 44, 46, 35, 48, 63, 50, 57, 38, 37, 61, 41, 40, 54, 65 and various members of the interprofessional

team (n=20).29-32, 56, 34, 45, 47, 66, 64, 49, 36, 39, 59, 60, 51-53, 55, 62

# Interventions to Support Implementation of Gerontological Knowledge

We developed the following categories to characterize the interventions that have been implemented to support the implementation of gerontological knowledge and promote practice change: level of intervention, care process changes, environmental changes, managerial support, facilitation/leadership roles, and discharge/transition planning. See Table 3. Of note, 16 studies reported on interprofessional interventions, with 16 relying on nurses to implement the intervention.

**Level of Intervention.** Interventions were categorized into three levels based on scale, with small-scale defined as a single care unit focused on one or two geriatric concerns, medium scale defined as one or more care units addressing multiple geriatric concerns, and large-scale defined as multiple hospitals addressing multiple geriatric concerns. There were four large-scale interventions, one included four hospitals working with regional health organizations in a formalized collaboration.^34^ The other three involved scale up/spread of two previously tested care models: the hospital elder life program (HELP) to 13 hospitals,^66^ and the EAT, WALK, ENGAGE program to multiple units in four hospitals.^59, 60^ Sixteen were medium-scale interventions that had multifocal geriatric protocols in one hospital and 12 were small-scale, focused on one intervention on one unit.

Medium-scale interventions focused on a variety of interventions that included combinations directed at cognition, pain, continence, mobility, nutrition/hydration, sleep and pressure ulcers, physical function/falls, family involvement, medication reviews/protocols, geriatric/frailty

screening, and volunteers.29-32, 40-43, 46, 35, 47-50, 57, 38, 39, 61, 53-55, 62 Of these, eleven involved nurses

implementing interventions,29, 42, 40-43, 46, 48, 50, 57, 61, 54 and the rest were interprofessional.

Large-scale studies focused on similar interventions to the medium-scale studies but were

implemented in multiple hospitals.^34, 66, 59, 60^ All involved an interprofessional approach.

**Care Process Changes.** Care process changes ranged from a single focused intervention to models of care that addressed multiple geriatric issues. Small-scale studies focused on

mobility,33, 58, 56, 64, 63, 36, 51, 65 cognition,45, 37 falls,52 or continence and mobility.44, 63 Of these, five

focused on nurses implementing interventions,^33, 58, 44, 63, 37^ while the remainder involved an interprofessional approach.

**Environmental Changes.** Environmental changes included walking trails (Boltz et al.,

2014,42, 63, 38, 39, 59-61, 65 orientation strategies (e.g. clocks),42, 35, 46, 47, 63, 48, 39, 59-61, 40, 54, 55,

modifications/decluttering,43, 33, 56, 35, 47, 66, 57, 61, 53 and sensory and mobility aids.60, 52, 54 One study examined the impact of adjusted seating and bed heights.^42^

**Administration/Management.** Administrative support included provision of

equipment,33, 56, 35, 66, 64, 63, 59, 51, 65 restraint policies,35, 66, 40, 55 electronic record modifications,33, 65 and additional staffing.^44, 59, 60^ Only one study involved regional inter-sectoral collaboration.^34^

**Facilitation/Leadership.** Facilitation/leadership roles in practice change included

advanced practice nurses,31, 44-47, 35, 66, 50, 57, 53, 41, 40, 54 geriatric resource nurse/staff,42, 32, 43, 58, 34, 45, 46, 48-50, 37, 38, 53, 55 and nurse supervisor/case managers.30, 42, 44, 45, 47, 57, 65 Additional personnel

included geriatricians,31, 43, 45-47, 35, 66, 49, 50, 40, 55 interprofessional resource persons,35, 47, 66, 64, 63, 53

and interprofessional geriatric teams.35, 59, 60, 51, 53, 41, 62

**Discharge/Transition Planning.** Discharge/transition planning included

interprofessional family meetings,^31, 32, 39^ links to discharge settings^30, 34, 47^ and minimum data set information.^34, 47^ The most common discharge/transition process involved patient follow-up.^31, 42,^

47, 66, 36, 37

# Outcomes Measured

Most studies reported on change in physical function (baseline to discharge).^30, 43, 33, 58, 56,^

44, 46, 64, 63, 49, 57, 37, 51, 61, 41, 52, 54, 65 Only a few studies used self-report33, 61 or caregiver report37 of

pre-hospital function as baseline; the remainder were performance-based assessments by

healthcare professionals. Six studies reported on cognitive function, primarily delirium,^32, 45, 48, 36,^ 50, 53 and 12 reported on both physical and cognitive outcomes.29, 31, 42, 35, 47, 66, 38, 39, 60, 40, 55, 62 All studies included nurse or interprofessional multifocal protocols. Two studies reported on a composite score: one an adverse outcome score measuring physical function and high healthcare demand post-hospital,^34^ and the other a composite of hospitalized outcomes such as delirium and pressure injury.^59^

# Discussion

In addition to education, a variety of interventions supported implementation of knowledge from the educational initiatives to improve functional outcomes for hospitalized older adults. We used a tripartite conceptualization to describe the scale of the interventions. Half the studies (18/38) concentrated on nurses for education and the delivery of the intervention. This could be because nurses make up almost half of the global health workforce^67^ and are the healthcare professionals that interact the most with patients in hospitals.^68^ In recent years, there has been increasing recognition of the importance of interprofessional collaboration in complex care.^10^ The trend from uni-professional interventions to multi-professional interventions was

observed in our findings; studies published in 2013 or earlier^43, 46, 35, 38, 57, 54^ tended to focus exclusively on nurse-driven interventions, whereas studies published in the last decade (e.g.,^29-31,^ 56, 34, 49, 39, 59, 51, 32, 62, 66) typically incorporated an interprofessional approach.

The studies reported that interventions to enhance knowledge uptake, in addition to gerontological education, had a measurable effect on functional outcomes, but were overwhelmingly physical and/or cognitive, with very few focusing on cognitive function exclusively. Cognitive function is now receiving more scholarly attention^68^ due to increasing recognition that cognitive and physical function are interconnected.^69^ However, our findings echo those of a recent systematic review^3^ which found that cognition is largely ignored; of 94 included studies, 93 focused exclusively on physical deconditioning.

Our review also highlights gaps in the existing research in terms of theory, research designs, intervention descriptions, country representation, policy, and environmental changes.

Included studies typically reported on time-limited initiatives and did not address sustainability. The WALK-FOR program is an exception as it was developed and tested on two units^33, 65,^ and for sustainability in a third study.^56^ Another model, EAT WALK ENGAGE, was tested for effectiveness on a single unit and then expanded to eight units in four hospitals,^59, 60^ but has not been examined for sustainability. Notably, the HELP model^46^ spread to other hospital sites and was assessed for sustainability.^66^ Moreover, elements of the HELP model were incorporated into other studies.^62^ Future reviews should examine the sustainability of these models/programs.

Another gap is the absence of theoretical frameworks – only three studies explicitly used

a structured framework to guide development/implementation of their intervention model,^59, 60, 65^ likely due to studies pre-dating recent advances in implementation science. Moreover, many included studies seemed to view change as a linear process, although current theoretical work emphasizes complexity and construes change as non-linear.^71, 27^ As such, some included studies failed to account for contextual elements previously identified as barriers to older adults’ care (e.g., short staffing, lack of resources, a medical focus, inter-professional team inaccessibility

during evenings and weekends).^10,^ ^72-76^ We suggest that future work in this area be guided by implementation theory.

Other gaps identified include a lack of rigorous designs, descriptions of interventions, education components, and representation. Only five of the 38 studies used a randomized controlled design. While we are conscious that randomized controlled trials (RCTs) – typically viewed as the gold standard in clinical research – are not always the most appropriate method for every research question and can be undermined by high costs, ethical considerations, and insufficient generalizability to broader, more diverse populations, we believe that more rigorous studies on the topic are needed. Such studies should include RCTs as well as qualitative and mixed methods approaches to understand the complexity of practice change. Descriptions of interventions were inconsistently presented, making it difficult to understand the education and other interventions provided to support implementation. A large number of studies were excluded during the review process because the education element was not described.

Included studies generally did not concentrate on policy and environmental changes, despite evidence that the built environment can contribute to improved functioning in older adults.^77^ Most of the included environmental changes were minor/superficial (e.g., clocks, decluttering). No studies described a major renovation to the built environment, possibly because the cost is prohibitive. There was little evidence of administrative changes to support

interventions; a few studies describing restraint policies or practices.^35, 66, 40, 55^ Most administrative support was minor except for Heim et al.,^34^ in which a system change was undertaken.

# Strengths and Limitations

This study adds to the literature on care for hospitalized older adults by synthesizing the evidence on education that supported intervention implementation, scaling/spread, and sustainability, and by providing a close examination of other interventions supporting the implementation of gerontological knowledge, which are typically overlooked in reviews. We also grounded our study in a robust theoretical conceptualization. We demonstrated that prior initiatives are mostly local in scope and piece-meal, with very few initiatives cutting across a healthcare system. We searched the literature in other languages, thereby accessing valuable studies that would have been excluded in an English-only review. However, we did not search the grey literature, dissertations/theses, or Clinical Practice Guidelines. Additionally, it is possible that education may have occurred in some studies that was not reported on (and therefore mistakenly excluded when assessing eligibility). We also focused our search on physical function and did not include details of cognitive interventions or other environmental interventions in our search terms (though we did extract any cognitive outcomes reported).

Future reviews should explicitly include cognitive function. Additionally, we adhered to the World Health Organization’s^78^ definition of older adults as aged 65+, admittedly an arbitrary cutoff point, and we thus excluded studies with younger definitions of older adult that may have otherwise been eligible. Finally, as with all scoping reviews, there is an unknown risk of publication bias due to the tendency of researchers to publish only positive findings. Finally, most studies were conducted in high-income countries and may not be generalizable to low- and middle-income countries.

# Directions for Future Research and Policy

We conceive of this scoping review, which mapped the breadth of the existing evidence, as a precursor to a future systematic review.^28^ Future systematic reviews should build on our work by examining which of the interventions we identified are most effective in optimizing the functioning of hospitalized older adults, and by comparing single interventions with multiintervention programs. Scholars should then develop strategies based on implementation science theory to ensure successful implementation of the most effective interventions while taking into consideration the realities of hospital units, such as short staffing, resource constraints, and overcrowding. Researchers reporting on similar interventions should describe the education component of interventions to enhance transparency and facilitate replication. Further work needs to be undertaken to integrate care of older persons across healthcare systems, and policylevel changes are required to facilitate the provisioning of function-oriented care.

# Funding

This work was supported by a Knowledge Synthesis Grant from the Seniors Health

Strategic Clinical Network™ (SCN), Alberta Health Services.

**Conflict of Interest**

We have no conflict of interest to declare.

# Acknowledgements

All data and materials are included in the main manuscript and supplementary materials. This research was pre-registered with the Open Science Framework, registration #: gr7nt; [https://doi.org/10.17605/OSF.IO/GR7NT.](https://doi.org/10.17605/OSF.IO/GR7NT)

References

1. Loyd C, Markland AD, Zhang Y, Fowler M, Harper S, Wright NC, Carter CS, Buford TW, Smith CH, Kennedy R, Brown CJ. Prevalence of hospital-associated disability in older adults: a meta-analysis. Journal of the American Medical Directors Association. 2020 Apr 1;21(4):455-61
2. Boucher EL, Gan JM, Rothwell PM, Shepperd S, Pendlebury ST. Prevalence and outcomes of frailty in unplanned hospital admissions: a systematic review and metaanalysis of hospital-wide and general (internal) medicine cohorts. EClinicalMedicine. 2023 May 1;59.
3. Chen Y, Almirall‐Sánchez A, Mockler D, Adrion E, Domínguez‐Vivero C, RomeroOrtuño R. Hospital‐associated deconditioning: Not only physical, but also cognitive. International Journal of Geriatric Psychiatry [Internet]. 2022 Feb 10;37(3). Available from: https://www.ncbi.nlm.nih.gov/pmc/articles/PMC9303382/
4. Manning L, Islam MdS. A systematic review to identify the challenges to achieving effective patient flow in public hospitals. The International Journal of Health Planning and Management. 2023 Feb 28;38(3).
5. Montero-Errasquín B, Cruz-Jentoft AJ. Acute sarcopenia. Gerontology. 2023 May 30;69(5):519-25.
6. Zisberg A, Shadmi E, Gur-Yaish N, Tonkikh O, Sinoff G. Hospital-Associated Functional Decline: The Role of Hospitalization Processes Beyond Individual Risk Factors. Journal of the American Geriatrics Society. 2015 Jan;63(1):55–62. 7. Dharmarajan K, Han L, Gahbauer EA, Leo‐Summers LS, Gill TM. Disability and Recovery After Hospitalization for Medical Illness Among Community‐Living Older Persons: A Prospective Cohort Study. Journal of the American Geriatrics Society. 2020 Feb 21;68(3):486–95.
8. Damschroder LJ, Aron DC, Keith RE, Kirsh SR, Alexander JA, Lowery JC. Fostering implementation of health services research findings into practice: a consolidated framework for advancing implementation science. Implementation Science. 2009 Aug 7;4(1).
9. Nilsen P, Bernhardsson S. Context matters in implementation science: a scoping review of determinant frameworks that describe contextual determinants for implementation outcomes. BMC Health Services Research. 2019 Mar 25;19(1).
10. Dahlke S, Hunter KF, Negrin K, Reshef Kalogirou M, Fox M, Wagg A. The educational needs of nursing staff when working with hospitalised older people. Journal of Clinical Nursing. 2018 Aug 28;28(1-2):221–34.
11. Swinnerton E, Price A. Recognising, reducing and preventing deconditioning in hospitalised older people. Nursing Older People. 2023 Feb 8;35(2).
12. Baldwin CE, Phillips AC, Edney SM, Lewis LK. Recommendations for older adults’ physical activity and sedentary behaviour during hospitalisation for an acute medical illness: an international Delphi study. International Journal of Behavioral Nutrition and Physical Activity. 2020 May 25;17(1).
13. Taylor NF, Harding KE, Dennett AM, Febrey S, Warmoth K, Hall AJ, et al. Behaviour change interventions to increase physical activity in hospitalised patients: a systematic review, meta-analysis and meta-regression. Age and Ageing. 2021 Jul 24;51(1). 14. Beltran SJ, Miller VJ. COVID-19 and Older Adults: The Time for GerontologyCurriculum across Social Work Programs is Now! Journal of Gerontological Social Work. 2020 Jul 7;1–4.
15. Dahlke S, Kalogirou MR, Swoboda NL. Registered nurses’ reflections on their educational preparation to work with older people. International Journal of Older People Nursing [Internet]. 2021 Mar 1;16(2):e12363. Available from: https://www.ncbi.nlm.nih.gov/pmc/articles/PMC7988531/#opn12363-bib-0040
16. Kalánková D, Stolt M, Scott PA, Papastavrou E, Suhonen R. Unmet care needs of older people: A scoping review. Nursing Ethics. 2020 Oct 1;28(2):096973302094811.
17. Tavares J, de Lurdes Almeida M, Duarte SFC, Apóstolo J. Older adult care in nursing education: How have curricula been developed? Nurse Education in Practice. 2021 Jan;50:102947.
18. Rangachari P, Rissing P, Rethemeyer K. Awareness of evidence-based practices alone does not translate to implementation: insights from implementation research. Quality management in health care [Internet]. 2013 [cited 2019 Oct 13];22(2):117–25. Available from: https://www.ncbi.nlm.nih.gov/pubmed/23542366
19. Kavanagh A, O’Brien L, Maloney S, Osadnik C. Barriers and facilitators to adopting functional maintenance initiatives for acutely hospitalised older adults. Disability and Rehabilitation. 2019 May 13;42(26):3808–15.
20. Soong C, Shojania KG. Education as a low-value improvement intervention: often necessary but rarely sufficient. BMJ Quality & Safety. 2019 Dec 16;29(5):353–7.
21. Hunter KF, Dahlke S, Negrin K, Kalogirou MR, Fox M, Antonio N, et al. The feasibility of implementing education on older person care to practice on medical units: Nurses’ perceptions and the influence of practice context. International Journal of Older People Nursing. 2019 Aug 22;14(4).
22. Peters MDJ, Marnie C, Tricco AC, Pollock D, Munn Z, Alexander L, et al. Updated Methodological Guidance for the Conduct of Scoping Reviews. JBI Evidence Synthesis. 2020 Sep 22;18(10):2119–26.
23. Hanson HM, Cowan K, Wagg A. Identifying what matters most for the health of older adults in Alberta: results from a James Lind Alliance Research Priority Setting Partnership. CMAJ Open. 2021 Apr;9(2):E522–8.
24. Campbell F, Tricco AC, Munn Z, Pollock D, Saran A, Sutton A, et al. Mapping reviews, scoping reviews, and evidence and gap maps (EGMs): the same but different— the “Big Picture” review family. Systematic Reviews. 2023 Mar 15;12(1).
25. Munn Z, Pollock D, Khalil H, Alexander L, McInerney P, Godfrey CM, et al. What are scoping reviews? Providing a formal definition of scoping reviews as a type of evidence synthesis. JBI Evidence Synthesis. 2022 Mar 4;20(4).
26. Tricco AC, Lillie E, Zarin W, O’Brien KK, Colquhoun H, Levac D, et al. PRISMA Extension for Scoping Reviews (PRISMA-ScR): Checklist and Explanation. Annals of Internal Medicine. 2018 Sep 4;169(7):467–73.
27. May CR, Johnson M, Finch T. Implementation, context and complexity. Implementation Science [Internet]. 2016 Oct 19 [cited 2024 Jul 16];11(1). Available from: https://www.ncbi.nlm.nih.gov/pmc/articles/PMC5069794/
28. Pollock et al., 2023. Pollock D, Peters MDJ, Khalil H et al. Recommendations for the extraction, analysis, and presentation of results in scoping reviews. JBI Evid Synth. 2023 Mar 1;21(3):520-32.
29. Allegri N, Rosi A, Del Signore F, Cremascoli R, Cappa S, Tassorelli C, et al. Dementiafriendly intervention for hospitalized older adults with cognitive impairments: results of the Italian Dementia-Friendly Hospital Trial (IDENTITÀ). Aging & Mental Health. 2021 Mar 24;1–9.
30. Apolinario D, Yagamuti STF, Dutra AF, de Sousa Lara EM, de Cassia Pires Coli R, & Weber B. Safe hospital for the elderly program: An observational study on the impact in reducing functional decline. *Cad. Saúde Pública*. 2022;*38*(2), 1–16
31. Bakker FC, Persoon A, Sebastian J.H. Bredie, Jolanda van Haren-Willems, Leferink VJ, Luc Noyez, et al. The CareWell in Hospital program to improve the quality of care for frail elderly inpatients: results of a before–after study with focus on surgical patients. 2014 Nov 1;208(5):735–46.
32. Bryant EA, Tulebaev S, Castillo-Angeles M, Moberg E, Senglaub SS, O’Mara L, et al. Frailty Identification and Care Pathway: An Interdisciplinary Approach to Care for Older Trauma Patients. Journal of the American College of Surgeons. 2019 Jun;228(6):852859.e1.
33. Cohen Y, Zisberg A, Chayat Y, Gur-Yaish N, Gil E, Levin C, et al. Walking for Better Outcomes and Recovery: The Effect of WALK-FOR in Preventing Hospital-Associated Functional Decline Among Older Adults. Newman A, editor. The Journals of Gerontology: Series A. 2019 Mar 2;74(10):1664–70.
34. Heim N, Rolden H, van Fenema EM, Weverling - Rijnsburger AWE, Tuijl JP, Jue P, et al. The development, implementation and evaluation of a transitional care programme to improve outcomes of frail older patients after hospitalisation. Age and Ageing. 2016 Jun 13;45(5):642–50.
35. Inouye SK, Bogardus ST, Charpentier PA, Leo-Summers L, Acampora D, Holford TR, et al. A Multicomponent Intervention to Prevent Delirium in Hospitalized Older Patients. New England Journal of Medicine [Internet]. 1999 Mar 4 [cited 2020 Jan 7];340(9):669–

76. Available from: https://www.nejm.org/doi/full/10.1056/NEJM199903043400901

36. Martínez-Velilla N, Garrués-Irisarri M, Ibañez-Beroiz B, Gil-Cabanas J, Richarte-García A, Idoate-Saralegui F, de Paz PC, Cambra K. An exercise program with patient’s involvement and family support can modify the cognitive and affective trajectory of acutely hospitalized older medical patients: a pilot study. Aging Clinical and Experimental Research. 2016 Jun;28:483-90.
37. Naylor MD, Hirschman KB, Hanlon AL, Bowles KH, Bradway C, McCauley KM, et al. Comparison of evidence-based interventions on outcomes of hospitalized, cognitively impaired older adults. Journal of Comparative Effectiveness Research. 2014 May;3(3):245–57.
38. Mudge AM, Giebel AJ, Cutler AJ. Exercising Body and Mind: An Integrated Approach to Functional Independence in Hospitalized Older People. Journal of the American Geriatrics Society. 2008 Apr;56(4):630–5.
39. Mudge AM, McRae P, Donovan PJ, Reade MC. Multidisciplinary quality improvement programme for older patients admitted to a vascular surgery ward ^*^. Internal Medicine Journal. 2020 Jun;50(6):741–8.
40. VidÃ¡n MT, SÃ¡nchez E, Alonso M, Montero B, Ortiz J, Serra JA. An Intervention Integrated into Daily Clinical Practice Reduces the Incidence of Delirium During Hospitalization in Elderly Patients. Journal of the American Geriatrics Society [Internet].

2009 Nov [cited 2019 Sep 16];57(11):2029–36. Available from: https://onlinelibrary.wiley.com/doi/10.1111/j.1532-5415.2009.02485.x

41. Suwanpasu S, Pongpaew W, Praparpak W. Geriatric resource nurse programme to maintain functional status of elderly patients hospitalised for community-acquired pneumonia. Asian Journal of Gerontology & Geriatrics. 2015 Dec;10(2):10.
42. Boltz M, Resnick B, Chippendale T, Galvin J. Testing a Family-Centered Intervention to Promote Functional and Cognitive Recovery in Hospitalized Older Adults. Journal of the

American Geriatrics Society. 2014 Dec; *62*(12):2398–2407

43. Chang E, Hancock K, Hickman L, Glasson J, Davidson P. Outcomes of acutely ill older hospitalized patients following implementation of tailored models of care: A repeated measures (pre- and post-intervention) design. International Journal of Nursing Studies. 2007 Sep;44(7):1079–92.
44. Hamilton L, Lyon PS. A Nursing-Driven Program to Preserve and Restore Functional Ability in Hospitalized Elderly Patients. JONA: The Journal of Nursing Administration. 1995 Apr;25(4):30–7.
45. Holt R, Young J, Heseltine D. Effectiveness of a multi-component intervention to reduce delirium incidence in elderly care wards. Age and Ageing. 2013 Aug 26;42(6):721–7. 46. Inouye SK, Wagner DR, Acampora D, Horwitz RI, Cooney LM, Tinetii ME. A

Controlled Trial of a Nursing-Centered Intervention in Hospitalized Elderly Medical

Patients: The Yale Geriatric Care Program. Journal of the American Geriatrics Society. 1993 Dec;41(12):1353–60.

47. Inouye SK, Bogardus ST, Baker DI, Leo-Summers L, & Cooney, L. M. The hospital elder life program: A model of care to prevent cognitive and functional decline in older hospitalized patients. Journal of the American Geriatrics Society. 2000;*48*(12):1697– 1706.
48. Kratz T, Heinrich M, Schlauß E, Diefenbacher A.Preventing postoperative delirium. Deutsches Ärzteblatt International, 2015;*112*:289–296.
49. Liu B, Moore JE, Almaawiy U, Chan WH, Khan S, Ewusie J, et al. Outcomes of Mobilisation of Vulnerable Elders in Ontario (MOVE ON): a multisite interrupted time series evaluation of an implementation intervention to increase patient mobilisation. Age and Ageing. 2017 Jul 15;47(1):112–9.
50. Milisen K, Foreman MD, Abraham IL, De Geest S, Godderis J, Vandermeulen E, et al. A Nurse-Led Interdisciplinary Intervention Program for Delirium in Elderly Hip-Fracture Patients. Journal of the American Geriatrics Society. 2001 May;49(5):523–32.
51. Peyrusqué E, Buckinx F, Bolduc A, Law C, Kergoat MJ, Aubertin-Leheudre M. Potential Efficacy of Pragmatic Exercise Program (Sprint) During Hospitalization in Older Adults on Health Care and Physical Performance: A Pilot Study. The Journal of nutrition, health & aging. 2020 Oct 12; *25*(1):126–133.
52. Von Renteln-Kruse W, Krause T. Incidence of In-Hospital Falls in Geriatric Patients

Before and After the Introduction of an Interdisciplinary Teamâ€ “ Based FallPrevention Intervention. Journal of the American Geriatrics Society. 2007 Dec;55(12):2068–74.

53. Rubin FH, Neal K, Fenlon K, Hassan S, Inouye SK. Sustainability and Scalability of the Hospital Elder Life Program at a Community Hospital. Journal of the American Geriatrics Society. 2011 Feb;59(2):359–65.
54. Wanich CK, Sullivan-Marx EM, Gottlieb GL, Johnson JC. Functional Status Outcomes of a Nursing Intervention in Hospitalized Elderly. Image: the Journal of Nursing Scholarship. 1992 Sep;24(3):201–8.
55. Wand APF, Thoo W, Sciuriaga H, Ting V, Baker J, Hunt GE. A multifaceted educational intervention to prevent delirium in older inpatients: A before and after study. International Journal of Nursing Studies [Internet]. 2014 Jul [cited 2019 Nov 21];51(7):974–82. Available from: https://www.sciencedirect.com/science/article/abs/pii/S0020748913003556
56. Gil E, Zisberg A, Efrat Shadmi, Nurit Gur-Yaish, Ksenya Shulyaev, Yehudith Chayat, et al. Still WALKing-FOR: 2-year sustainability of the “WALK FOR” intervention. Age and ageing. 2023 Jun 1;52(6).
57. Miller J, Campbell J, Moore K, Schofield A. Elder Care Supportive Interventions Protocol: Reducing Discomfort in Confused, Hospitalized Older Adults. Journal of Gerontological Nursing. 2004 Aug 1;30(8):10–8.
58. Gazineo D, Godino L, Decaro R, Calogero P, Pinto D, Chiari P, et al. Assisted Walking

Program on Walking Ability in In‐Hospital Geriatric Patients: A Randomized Trial. Journal of the American Geriatrics Society. 2020 Nov 12;69(3):637–43.

59. Mudge AM, McRae P, Banks M, Blackberry I, Barrimore S, Endacott J, et al. Effect of a Ward-Based Program on Hospital-Associated Complications and Length of Stay for Older Inpatients. JAMA Internal Medicine. 2022 Mar 1;182(3):274.
60. Mudge A, McRae P, Young A, Blackberry I, Lee‐Steere K, Barrimore SE, et al. Implementing a ward-based programme to improve care for older inpatients: process evaluation of the cluster randomised CHERISH trial. BMC Health Services Research. 2023 Jun 21;23(1).
61. Rodrigues C, Mendonça D, Martins MM. Effects of a nursing care program focused on basic self-care in older acute medical in-patients: a randomized controlled trial. Porto Biomedical Journal. 2020 Nov;5(6):e086.
62. Wang YY, Yue JR, Xie DM, Carter P, Li QL, Gartaganis SL, et al. Effect of the Tailored, Family-Involved Hospital Elder Life Program on Postoperative Delirium and Function in Older Adults. JAMA Internal Medicine. 2020 Jan 1;180(1):17.
63. King BJ, Steege LM, Winsor K, VanDenbergh S, Brown CJ. Getting Patients Walking: A Pilot Study of Mobilizing Older Adult Patients via a Nurse-Driven Intervention. Journal of the American Geriatrics Society. 2016 Aug 22;64(10):2088–94.
64. Juneau A, Bolduc A, Nguyen P, Leclerc BS, Rousseau J, Dubé F, et al. Feasibility of Implementing an Exercise Program in a Geriatric Assessment Unit: the SPRINT Program. Canadian Geriatrics Journal. 2018 Sep 14;21(3):284–9.
65. Zisberg A, Agmon M, Gur-Yaish N, Rand D, Hayat Y, Gil E. No one size fits all—the development of a theory-driven intervention to increase in-hospital mobility: the

“WALK-FOR” study. BMC Geriatrics. 2018 Apr 13;18(1).

66. Inouye SK, Baker DI, Fugal P, Bradley EH. Dissemination of the Hospital Elder Life Program: Implementation, Adaptation, and Successes. Journal of the American Geriatrics Society. 2006 Oct;54(10):1492–9.
67. World Health Organization. Nursing and Midwifery; 2022.

[https://www.who.int/news-room/fact-sheets/detail/nursing-and-midwifery.](https://www.who.int/news-room/fact-sheets/detail/nursing-and-midwifery)

68. Butler R, Monsalve M, Thomas GW, Herman T, Segre AM, Polgreen PM, et al. Estimating Time Physicians and Other Health Care Workers Spend with Patients in an Intensive Care Unit Using a Sensor Network. The American Journal of Medicine [Internet]. 2018 Aug;131(8):972.e9–15. Available from: https://www.ncbi.nlm.nih.gov/pubmed/29649458
69. Fox MT, Sidani S, Butler JI, Skinner MW, Macdonald M, Durocher E, et al. Optimizing hospital-to-home transitions for older persons in rural communities: a participatory, multimethod study protocol. Implementation Science Communications. 2021 Jul 22;2(1).
70. Yi Chia Wei, Heather C, Wen Yi Huang, Yao Liang Chen, Lin C, Chih Ken Chen, et al. White Matter Integrity Underlies the Physical-Cognitive Correlations in Subjective Cognitive Decline. Frontiers in Aging Neuroscience. 2021 Aug 2;13.
71. Lanham HJ, Leykum LK, Taylor BS, McCannon CJ, Lindberg C, Lester RT. How complexity science can inform scale-up and spread in health care: understanding the role of self-organization in variation across local contexts. Social science & medicine. 2013 Sep 1;93:194-202.
72. Dermody G, Kovach CR. Nurses’ Experience With and Perception of Barriers to Promoting Mobility in Hospitalized Older Adults: A Descriptive Study. Journal of Gerontological Nursing. 2017 May 30;43(11):22–9.
73. Fox MT, Butler JI. Nurses’ perspectives on how operational leaders influence functionfocused care for hospitalised older people. Journal of Nursing Management. 2016 Sep 16;24(8):1119–29.
74. Hower KI, Vennedey V, Hillen HA, Kuntz L, Stock S, Pfaff H, et al. Implementation of patient-centred care: which organisational determinants matter from decision maker’s perspective? Results from a qualitative interview study across various health and social care organisations. BMJ Open [Internet]. 2019 Apr;9(4):e027591. Available from: https://bmjopen.bmj.com/content/9/4/e027591
75. Sepulveda-Pacsi AL, Soderman M, Kertesz L. Nurses’ perceptions of their knowledge and barriers to ambulating hospitalized patients in acute settings. Applied Nursing Research. 2016 Nov;32:117–21.
76. Yous ML, Ploeg J, Kaasalainen S, Martin LS. Nurses’ Experiences in Caring for Older Adults with Responsive Behaviors of Dementia in Acute Care. SAGE Open Nursing [Internet]. 2019 Jan;5. Available from: https://journals.sagepub.com/doi/full/10.1177/2377960819834127
77. Parke B, Boltz M, Hunter KF, Chambers T, Wolf-Ostermann K, Adi MN, et al. A Scoping Literature Review of Dementia-Friendly Hospital Design. The Gerontologist. 2016 Nov 9;57(4):gnw128.
78. World Health Organization. By 2024, the 65-and-over age group will outnumber the youth group: new WHO report on healthy ageing; 2023. https://www.who.int/europe/news/item/11-10-2023-by-2024--the-65-and-over-agegroup-will-outnumber-the-youth-group--new-who-report-on-healthy-ageing

**S-1 Table:** Preferred Reporting Items for Systematic reviews and Meta-Analyses extension for Scoping Reviews (PRISMA-ScR) Checklist

610	**Table 1.**

# 611 *Table of Included Studies (N=38)*

**Author (year)	Method, study aim, sample	Education component	Interventions in addition to	Outcomes assessed**

## Country setting education and relevant

**findings**

| Allegri et al.  (2022).  Italy | **Method**  Pre-post with group matching analysis (Quasi-experimental designs)  **Objective**  To evaluate whether a short training focused on improving dementia care practices of the hospital staff was able to counteract functional loss and to decrease negative outcomes at discharge | A five-hour training course, including five one-hour teaching modules were delivered to hospital staff (doctors, nurses, physical therapists and healthcare assistants) working in the medical units involved in the study.  Module 1 - improving the detection of signs and symptoms of dementia and cognitive impairment, depression, BSPD, | **Level of intervention-** medium scale, unit based single hospital model of care.  **Care process change:** Italian  Dementia-Friendly Hospital trial (IDENTITA) was implemented. Focus on cognitive impairment including dementia, delirium, depressionBSPD, medication, pain and communication | **Functional outcomes** Functional status measured using modified Barthel index.  **Cognitive outcomes** Cognitive impairment measured using  MMSE. |
| --- | --- | --- | --- | --- |

among hospitalized older adults with cognitive impairment.

## Sample

68 hospitalized patients aged 65 years and over with cognitive impairment (intervention group: 34 and control group:34). **Setting** General Medicine, Surgery, and Orthopedics units

Apolinario et al.	**Method**

(2022).	Prospective cohort program

Brazil	evaluation (design not

reported).

(Quasi-experimental designs)

and specific needs of the patients. Module 2 - drug prescription. Module 3 - management of BPSD and delirium., Module 4 - detection of pain. Module 5 - communication and interaction with patients.

Education targeted to professionals to interpret the results of the frailty screening questionnaire and the AMPI

(Multidimensional Assessment of **Level of intervention-** medium scale,	**Functional outcomes** single hospital, model of care	Functional decline **Care process change:**	assessed by Barthel

Implementation of the Safe Hospital	index.

Program for the Elderly developed

**Objective** To describe the Safe Hospital for the Elderly Program and assess the initial results of its implementation in a philanthropic hospital in the city of Sao Paulo.

## Sample

865 frail hospitalized older adults (582 in the implementation period and 283 in the consolidation period). **Setting**

Philanthropic hospital

the Elderly), and acquire basic knowledge about eight complications in the hospital environment: (1) fall, (2) pressure injury, (3) bronchoaspiration, (4) delirium, (5) mechanical restraint, (6) malnutrition, (7) medication iatrogenesis, and (8) functional decline. Staff were also given resources such as booklet, video lessons, infographics, on-site small group training, and training workshops. by a multidisciplinary team. Frailty	Occurrence of one or screening used to select patients.	more falls during

Multidisciplinary assessment that	hospitalization.

included cognitive, delirium,

depressive symptom, ADLs, mobility, nutrition screening, risk of bronchial aspiration, medications at risk, communication and social support. Protocols on prevention of falls, pressure injury, bronchial aspiration, prevention, detection and management of delirium and rational use of mechanical restraints, mobility stimulation, screening of inappropriate medications, approach to the elderly, and nutritional risk.

Multidisciplinary rounds to discuss cases.

**Facilitation**

## Nurse case manager Discharge planning or care transition planning

Identification of factors related to long stay, map of de-hospitalization resources and development of a discharge planning program with emphasis on transitions of care.

Bakker et al.	**Method**	Physicians and nurses receive	**Level of intervention-** medium scale,	**Functional outcomes**

(2014)	Before-after study including	group education from the	single hospital, model of care	Physical functioning

Netherlands	mixed-methods process and an	geriatrics team on components of	**Care Process Change**	assessed using

effect evaluation.	the CareWell in Hospital program	The CareWell in Hospital (CWH)	Groningen Activity

(Quasi-experimental designs)	including a coaching at work.	program (based on the Hospital	Restriction Scale

**Objective**	Elder Life Program (HELP) Inouye et	(GARS)

To evaluate the implementation of the CareWell in Hospital program, which is an innovative intervention designed to prevent complications and stimulate early rehabilitation among frail elderly inpatients.

**Sample** 191 pre-intervention and 195 post-intervention

70 years and older

**Setting** 2 surgical wards and 1 internal medicine

al 2000) was developed and pilot tested.

Development of a care pathway for screening and management of

patients at risk for geriatric syndromes.

Intervention included:

Screening, medication review, Carewell plan (recommendations about care and well-being) in: somatic problems, physical functioning, social environment, psychosocial functioning, and communication), follow-up, geriatric assessments, multidisciplinary meetings, therapeutic activities.

Team of trained volunteers.

## Cognitive outcomes

Delirium assessed by Confusion

Assessment Method Cognitive functioning assessed by Dutch version of the MiniMental State

Examination (MMSE).

|  |  |  | **Facilitation**  Coaching from the geriatric team (geriatric nurse specialist and geriatrician). |  |
| --- | --- | --- | --- | --- |
| Boltz et al. (2014)  United States | **Method**  Comparative repeated measures design with intervention and control arm.  (Quasi-experimental designs)  **Objective**  To test the feasibility of Family-centered Functionfocused Care (Fam-FFC) and to examine the impact of FamFFCon functional outcomes of hospitalized older medical patients. | 1^st^ month nursing staff receive education session on prevention and management of delirium and functional decline; assessment of mood, cognition, and function; incorporation of function-focused care into care routines; ways to communicate, partnering, and motivating patients and family; and discharge planning. On the 2^nd^, 3^rd^, and 4^th^ month, educational reminders where distributed to the intervention | **Level of intervention-** medium scale, single hospital, model of care **Care Process Change**  The implementation of Family centered function focused care (Fam-FFC) which includes environmental modification, education of family members, and involvement of patient and family in their plan of care from admission to after discharge.  **Environmental Changes** | **Functional outcomes** ADL performance assessed by Barthel index (walking item to measure walking performance) Gait and balance assessed by Tinetti Scale.  **Cognitive outcomes**  Delirium assessed by  Delirium Severity  Scale. |

**Sample**	group through staff mailboxes

97 dyads (partnership between	and educational boards.

older patient and a caregiver) **Setting** Three medical units of a community teaching hospital Assessment and modifications on	the environmental layout of the units such as lighting, seating, bed height, toilet height, communication boards for staff and patients, and accessibility of sensory and mobility aids.

**Administrative decisions** Patient and/or family members are included in shift reports. **Facilitation**

Family-centered resource nurse (FCRN) (RN with experience in gerontology and certified in gerontology) 10 hours a week. Nursing supervisor as unit champion for the study.

|  |  |  | **Discharge planning/transition care**  Follow up by FCRN |  |
| --- | --- | --- | --- | --- |
| Bryant et al.  (2019).  USA | **Method**  Retrospective cohort study (Quasi-experimental designs, comparison of pre-post intervention groups) **Objective**  To determine if an interdisciplinary care pathway for frail trauma patients improved in-hospital mortality, complications, and  30-day readmissions.  **Sample**  269 patients frail patients aged  ≥65 years admitted to the | Residents and physician assistants received an educational presentation and a pocket card for easy reference. Educational efforts for all the nurses, ancillary staff, residents, and attending staff who frequently care for older patients in trauma units and in the ICU. Mandatory educational modules were also developed. | **Level of intervention-** medium scale, single hospital, model of care  **Care process changes**  A standardized, evidence-based interdisciplinary pathway of care was developed and tested to reduce delirium and complications in frail geriatric trauma patients. The domains of the pathway included: on the pathway for delirium prevention, mobility, medication management, prognostic assessment, reducing other complications, social needs/goal setting and injury prevention. | **Cognitive outcomes**  Delirium assessed by  Confusion  Assessment Method. |

trauma service (Preintervention: 125 and postintervention: 144) **Setting**

Trauma center

Patients 65 and older screened for frailty in ER. Standardized order set for patients (orders included early mobilization, nutrition, delirium prevention, identifying proxy, advanced directives and geriatric physio, nutrition and SW consults. All patients receive fall prevention education prior to discharge. Interdisciplinary bi-weekly rounds with trauma geriatrician.

**Administrative decisions**

**Facilitation**

Nurse champions.

## Discharge planning/transition care

|  |  |  | Interdisciplinary family meeting for hospital length of stay of more than  5 days. |  |
| --- | --- | --- | --- | --- |
| Chang et al. (2007)  Australia | **Method**  Part of multi-phase design. This study is comparative repeated measures design (Quasi-experimental design, comparison of pre-post intervention groups)  Note:  Part of a larger Mixed Method – Participatory Action Research including Chang (2003)  **Objective**  To compare the efficacy of a | The education was delivered to nurses through in service type meetings over a span of 3-week period. The different models of care specific to older adults in acute care were discussed. The key reference group provided feedback to other nurses, which facilitated ongoing learning among them. | **Level of intervention-** medium scale, single hospital, model of care **Care Process Changes** Nurses participated in action research phase to develop model of care for their unit Guided by Orem’s self-care and the NICHE medication protocol concepts. Interdisciplinary team on aged care ward included geriatrician. Communication among nurses about patients’ functional status and knowledge medication.  **Environmental Changes** | **Functional outcomes**  ADL assessed by  Barthel index. |

model of care for older adults

|  | when implemented to (a) an aged care specific ward and (b) a standard medical ward admitting older adults.  **Sample**  56 older adult patients from the aged care ward group and 60 older adult patients from the standard medical ward group  **Setting**  Acute Medicine Units |  | Physical environmental modifications were implemented to address the need of older adults such as railings for fall prevention and patients assigned closer to nursing stations.  **Facilitation**  Each ward created a key reference group of 3 to 5 nurses, who collaborated with the research team to tailor the model to the context of their ward as well as facilitate sharing of knowledge between nursing staff. |  |
| --- | --- | --- | --- | --- |
| Cohen et al.  (2019).  Israel | **Method**  A quasi-experimental pre-post two group comparative design. | Staff (nurses and nurse aides) underwent theoretical and | **Level of intervention-** small scale, single hospital, single focus  **Care process changes:** | **Functional outcomes** Individual’s subjective |

## Objective

To examine the effect of

WALK-FOR on the prevention of hospital-associated functional decline (HAFD) at discharge and at 1-month follow-up from acute hospitalization in internal *medical units.*

## Sample

Intervention group: 188

Control group: 189

## Setting

Two hospital internal medical units, each unit consisting 46 bed-in a medical center practical training to assess mobility and to safely mobilize patients.

Implementation of the WALK -FOR mobility program Unit-tailored mobility programs were structured for unit staff, patients, and their caregivers, which were distributed through in-personal communication, video clips, brochures, and posters. Nurses assessed mobility and provided mobility recommendations at admission.

**Environmental** Environmental modifications included removing physical obstacles from corridors, marking walking trails, and purchasing extra walkers. **Administrative decisions**

assessment of their independence level in performing basic activities of daily living (BADL) operationalized as decrease of 5 points or more in BADL score, assessed by

Modified Barthel Index.

Instrumental

Activities of Daily Living (IADL) assessed using

Lawton’s IADL eight

|  |  |  | Electronic medical records were modified to include the patient's mobility ability as well reports on daily walking distance. | item scale for premorbid status. Community mobility assessed using an activity summary index (subscale of Yale Physical Activity  Survey (YPAS). |
| --- | --- | --- | --- | --- |
| Gazineo et al.  (2021)  Italy | **Method**  Randomized Controlled Trial  **Objective**  To evaluate if an individualized assisted walking program (IAWP) for hospitalized older patients could improve walking ability compared with usual | One nurse received training in the walking protocol and patient and family education (not the full team). Very limited description of training. | **Level of intervention-** small scale, single hospital, single unit, single focus  **Care process changes: Mobility** Individualized assisted walking program (IAWP) was delivered to an intervention group by a trained nurse. The IAWP program included early assessment within 24 hours of | **Functional outcomes** Change in walking ability (hospital admission to discharge) assessed with Braden Activity subscale (BAS). |

geriatric care and rehabilitation.

## Sample

Of the total 387 included patients (193 were randomly assigned to the intervention group and 194 to the usual/control group. **Setting** 32-bed geriatric unit of a university hospital in Bologna,

Italy.

admission, moving from the supine to sitting position and from sitting to standing position, supervised ambulation and education about the importance of daily ambulation for patients and their caregivers. Intervention group patients were helped to move from the supine to sitting position with their legs hanging over the side of the bed, from sitting to standing and an active phase of walking with assistance. Intervention was carried out in daily basis (except weekends) for 20-30 minutes and continued from the first day of admission until the day before discharge.

|  |  |  | **Facilitation**  A daily briefing session was held between the trained nurse and the geriatrician in charge prior to intervention to assess the feasibility for each patient. |  |
| --- | --- | --- | --- | --- |
| Gil et al. (2023).  Israel | **Method**  Quasi-experimental threegroup comparative design (pre-implementation usual care, immediate intervention and 2 year post implementation groups) .  **Objective**  To explore whether the WALKFOR (walking for better outcomes and recovery) | Unit staff, including nurses, nurses’ aides and physical therapists received theoretical and practical training on the  WALK-FOR intervention program with the aim to raise knowledge and awareness of mobility during hospitalization.  Note: Education component reported in original report Cohen et al. (2019) (cited by authors) | **Level of intervention-** small scale, single hospital, single unit, single focus  **Care process changes**  The WALK-FOR intervention program was designed to promote in-hospital mobility of patients.  Patients were asked to walk on the marked trail along the corridor at least twice daily in order to complete at least 900 | **Functional outcomes** Mobility during hospitalization was defined as the average daily number of steps (monitored by ankleworn accelerometers) Selfreported range and frequency of their |

|  | intervention’s effect on the daily step count of acutely ill older adults is sustainable 2 years after its implementation.  **Sample**  (pre-implementation or control group: 150; immediate post-implementation: 144 and  2-year post implementation:  72)  **Setting**  46-bed internal medicine units in a medical center in Northern  Israel. |  | steps. Patients wore accelerometers (Actical) on the ankle which were used to monitor daily step counts up to 3 days.  **Environmental**  Physical obstacles were removed from corridors, walking trails were marked.  **Administrative decisions**  Extra-walkers were purchased. | mobility inside and outside of their rooms. Yale Physical Activity Survey activity summary index to report  Community mobility. Also measured ADL by Modified Barthel index, IADL by  Lawton IADL scale. |
| --- | --- | --- | --- | --- |
| Hamilton & Lyon  (1995).  Canada | **Method**  A quasi-experimental, onegroup pretest/post-test design | Geriatric module included three resource manuals: (1) Urinary continence in the elderly: A nurse | **Level of intervention-** small scale, single hospital, single unit, 2 foci  **Care process changes** | **Functional outcomes** Functional status assessed using |

## Objective

To evaluate effectiveness of a 6 bed module for geriatric assessment and rehabilitation patients’ functional status.

## Sample

74 patients aged 65 years and older with medical stability (did not require bed rest) and functional impairment in one or more areas of ADL.

Those with contractures (affecting mobility) and severe cognitive impairment were excluded. **Setting**

program, (2) Improving functional mobility in the elderly: A nursing program, and (3) The geriatric module manual (included policies and procedures). Staff who rotated in the geriatric module for 8-week periods were oriented to the module and the study. Staff received education on urinary incontinence and mobility programs from clinical nurse specialists (service purchased from another hospital for 18 month development and support

period) .

Mobility and urinary incontinence program delivered by nurses. **Administrative decisions** Enhanced nursing resources during day shift in 6 bed module (1.5 FTE added); medical unit staff covered evenings and nights. **Facilitation**

Clinical nurse specialist available on site 1 day per week. Nurse manager implemented modular nursing and provided consistent administrative support.

Barthel index of activities of daily living at admission to module from medical unit, every two weeks and discharge.

**Cognitive outcomes** Cognitive function assessed by MMSE.

|  | 6 bed geriatric assessment and rehabilitation module within a 70 bed medical unit in a 258 bed community hospital |  |  |  |
| --- | --- | --- | --- | --- |
| Heim et al. (2016)  Netherlands | **Method**  Pre-program vs post program implementation  (Quasi-experimental designs)  **Objective**  Report on development, implementation and evaluation of a regional transitional care programme aimed at improving the recovery rate of frail hospitalized older patients (70+). | Educational component for HCPS  - two aspects:  Nursing/medical staff were educated on frailty in older patients.  The trained nurses collaborated on the units to educate staff to facilitate rehabilitation. They organized clinical lessons, but mostly trained on the job. Staff of the rehabilitation unit was educated to be better able to care for early discharged patients | **Level of intervention-** large scale, healthcare system, model of care  **Care Process Change -** A screening tool (ADL, undernutrition, falls, delirium) and triage instrument for frailty and related protocols were developed, developed for the hospital component. In hospital Geriatric consultation teams to follow-up on screening results. Care protocols for nurses (details not provided).  Weekly multidisciplinary meetings | **Functional outcomes** ADL functioning assessed using Katz index on independence.    Nutritional status assessed using short nutritional assessment questionnaire  (SNAQ) |

| **Sample**  Data on functional decline were complete for 721 (frail) and 827 (non-frail) patients who filled out and returned the self-administered questionnaire after 3 months of follow-up.  **Setting**  Four hospitals working with regional health care organizations (nursing home, rehabilitation centre) | to enable patients to be transferred to the well suiting unit sooner. | focused on improving/preserving  ADL functioning.  **Environmental**  No hospital environment changes. Nursing home unit (discharge destination option) refurbished to meet needs of patients with cognitive disorders and a rehabilitation need.  **Administrative decisions** Regional interorganizational program development, implementation, evaluation Multi party commitment to the transitional care program.  **Facilitation** | Composite endpoint of adverse outcome (ADL function and/or high health care demand 3 months  after hospital admission)  **Cognitive outcomes** Risk of delirium assessed using researcher developed questions. |
| --- | --- | --- | --- |

Every unit appointed a nurse responsible to implement the protocols in his/her unit. Each organization appointed staff member responsible for the internal implementation of a digital transfer system. **Discharge planning or care transition planning** Triage instrument for best discharge destination and follow up care. Links to rehabilitation unit outpatient unit in GP Office, minimum data set for transfers developed (digital transfer, shared expertise between hospital, nursing home, rehabilitation centre

| Holt et al. (2013).  UK | **Method**  Before and after study  (Quasi-experimental designs)  **Objective**  To examine the effect of a multi-component, delirium prevention intervention on rates of incident delirium for patients admitted to specialist elderly care wards.  **Sample**  436 patients (Before group:  249 and After group: 187)  **Setting**  Three specialist elderly care wards (each 80-bed) in a | The research team provided education and practice change materials to the ward staff (staff nurses, healthcare assistants). Educational - 30 min interactive lecture with a handout, a delirium quiz, a poster, reference material and case vignettes.  Practice change materials - delirium risk factor modification care plan, a delirium assessment protocol for ward doctors and an escalation flowchart for suspected delirium for nurses. | **Level of intervention-** small scale, single hospital, 3 units, single focus  **Care process changes**  Delirium prevention/ intervention on specialized units consisted of standardized education and delirium, risk factor modification protocol materials. Practice change included delirium risk factor modification care plan placed at end of patient bed, required signed actions 3 times a day. Delirium assessment protocol for ward physicians and escalation flow chart of suspected delirium for nurses.  **Facilitation** | **Functional outcomes** Function at discharge assessed using Barthel index score.  **Cognitive outcomes** Delirium was assessed by daily assessments and  CAM (Confusion Assessment Method) and DRS-R-98  (Delirium Rating Scale-Revised-98) instruments. |
| --- | --- | --- | --- | --- |

general hospital

|  |  |  | Specialist nurse, consultant geriatrician and nurse manager. Volunteer nurse from each ward group acted a “link” |  |
| --- | --- | --- | --- | --- |
| Inouye et al. (1993b). A controlled trial of a nursing-centered intervention in hospitalized elderly medical patients: The Yale  Geriatric Care  Program.  USA | **Method**  Prospective cohort study with stratified and matched cohort analyses.  (Quasi-experimental designs)  **Objective**  To test the effectiveness of a nursing-centered intervention to prevent functional decline among hospitalized elderly medical patients. *(Yale*  *Geriatric Care Program)*  **Sample** | Geriatric resource nurses were provided special training and education in geriatric nursing and were key intervention figures. In addition, unit-based geriatric lecture series were provided for all nurses.  NOTE: Education component described in model protocol/description Inouye et al. (1993a) (published in same journal issue) | **Level of intervention-** medium scale, single hospital, units, model of care.  **Care process changes**  The Yale Geriatric Care Program designed to develop and integrate geriatric nursing expertise as a part of standard nursing care on each intervention unit.  Nurse/physician Intervention included identification of frail older patients with subsequent daily surveillance, twice-weekly rounds of the geriatric care team (nurse | **Functional outcomes** Pressure ulcer assessed using standardized skin check. Patients selfreported their ability to perform ADLs, information on incontinence and  social activity level.  **Cognitive outcomes** |

| 216 patients aged ≥70 years (intervention group: 85 and control group: 131)  **Setting**  Five general medicine wards of a 800 -bed urban teaching hospital (Yale-New Haven Hospital). Two intervention units (one was “nurse and physician intervention, the other was “nurse only intervention” plus three control units). | focused interventions), special education and support for the geriatric resource nurses, and a unitbased geriatric lecture series for all nurses.  Nurse-only intervention’ which was the same as above, except that the geriatric physicians did not participate in rounds.  Clinical protocols to address six targeted conditions (Geriatric Vital Signs)- Skin impairment, undernutrition, incontinence, confusion (delirium/dementia), falls or functional impairment, sleep disturbance. **Facilitation** | Cognitive impairment was assessed by MMSE  Delirium by  Confusion  Assessment Method. |
| --- | --- | --- |

|  |  |  | Geriatric resource nurses (GRN), a masters-prepared gerontological nurse specialist (GNS), and two geriatric physicians. Geriatric Care Team (Geriatric resource nurses, gerontological nurse specialist, geriatric physician, primary nurses of involved patients) twice weekly rounds.  **Discharge Planning** - included in nursing education details not provided |  |
| --- | --- | --- | --- | --- |
| Inouye et al.  (1999).  USA | **Method**  Controlled clinical trial  (Quasi-experimental designs) | Research nurses and experienced clinical researchers received intensive training and followed standard procedures outlined in a | **Level of intervention-** medium scale, single hospital, 1 unit, model of care.  **Care process change** | **Functional outcome**s  Activities of Daily  Living assessed using  Katz’s index of |

| **Objective**  To evaluate the effectiveness of multicomponent strategy for the prevention of delirium  *(Elder Life Program)*  **Sample**  N= 852 patients assessed as at risk for delirium, matched as 426 pairs of patients receiving the study intervention and usual care.    **Setting**  800-bed urban teaching hospital with 200 medical beds. Study took place on one | detailed training and coding manual on delirium risk factorscognitive impairment, sleep deprivation, immobility, visual impairment, hearing impairment and dehydration. | Elder Life Program was implemented by a trained interdisciplinary team and volunteers.  Intervention protocol focused on 6 target risk factors for delirium (cognitive impairment, sleep deprivation, immobility, visual impairment, hearing impairment, dehydration) and included orientation protocol, therapeuticactivities protocol, nonpharmacologic sleep protocol (warm drink, relaxation music and back massage at bedtime), early mobilization protocol, vision protocol, hearing protocol, dehydration (including early | activities of daily living.  **Cognitive outcomes** Delirium assessed using Confusion  Assessment Method. |
| --- | --- | --- | --- |

intervention medical unit and two usual care medical units recognition), protocol to improve cognition, sleep, mobility, vision, hearing, and fluid intake respectively. Cognitive simulation activities included discussion of events, structured reminiscence, or word games.

**Environmental** Board with names of care-team members and daily schedule kept as orientation protocol. Noise reduction strategies: Maintaining quiet hallways, silent pill crushers, vibrating beepers, rescheduling medications and procedures. Adaptive equipment (large print books, fluorescent tape on call bell)

|  |  |  | **Administrative decisions** Minimizing use of immobilizing equipment (e.g., physical restraints), encouraging ambulation. Accessible portable amplifying devices, special communication techniques.  **Facilitation** Team included of geriatric nurse-specialists, two specially trained Elder Life  Specialists, a certified therapeutic recreation specialist, a physical therapy consultant, and a geriatrician. |  |
| --- | --- | --- | --- | --- |
| Inouye et al.  (2000)  United States | **Method**  Cohort (not reported, but seems like cohort). | Education program delivered by Elder Life nurse specialist, geriatrician and nurse | **Level of intervention-** medium scale, single hospital, 3 units, model of care. | **Functional outcomes**  Functional decline  (tool not given) |

(Quasi-experimental designs)

## Objective

To describe the *Hospital Elder Life Program (HELP),* a model of care designed to prevent functional and cognitive decline of older persons during hospitalization. Specifically, describe: (1) the roles and responsibilities of key personnel; (2) the setting and administrative structure· (3) the process of care -including interventions, screening; and enrollment procedures, the intervention process

practitioners to nurses, physicians and volunteers about Elder Life issues- included orientation protocol, therapeutic activities, early mobilization, vision/hearing, oral volume repletion, feeding assistance, sleep enhancement, geriatric nursing assessment and interventions. Formal didactic sessions, small group training, and resource materials.

## Care process changes Cognitive outcomes

Implementation of the Hospital Elder	Delirium (tool not

Life Program. All patients aged ~70	given). years on specified units screened on	admission for 6 risk factors (cognitive impairment, sleep deprivation, immobility, dehydration, vision or hearing impairment). Targeted interventions for these risk factors (daily visitor/orientation, therapeutic activities (cognitive stimulation), early mobilization (ambulation or active range of motion 3 times daily), vision protocol, hearing protocol, oral volume repletion, feeding assistance, sleep protocol (e.g

and adherence rates, and quality assurance measures; (4) the outcomes and benefits of the program; (5) program costs and sources of funding; and (6) issues pertinent to dissemination to other hospitals.

## Sample

1507 patients aged 70+.

**Setting** Three general medicine nursing units at Yale-New Haven Hospital (800-bed urban teaching hospital with 200 medical beds)

rescheduling medications and procedures) implemented by an interdisciplinary team that included Elder Life Specialists (see Facilitation below) , trained volunteers, and geriatricians who work closely with primary nurses. Interdisciplinary team for other interventions, consultation expert at twice-weekly interdisciplinary rounds for each patient. Recommended interventions tracked. **Environmental** Orientation board with names of care team members and daily schedule. Unit-wide noise-reduction strategies (e.g. silent pill crushers,

vibrating beepers, and quiet hallways).

**Administrative decisions** Monitoring of intervention adherence (quality assurance). **Facilitation**

Key HELP staff included Elder Life Nurse Specialist, Elder Life Specialist/volunteer coordinator, geriatrician, program director (one of above). Geriatrician consultation Interdisciplinary consultation as needed. Volunteer recognition incentive awards. **Discharge planning or care transition planning**

|  |  |  | Discharge communication form. Community linkages and telephone follow-up. Referrals and communication with community agencies to optimize transition home. Telephone follow-up within 7 days after discharge. |  |
| --- | --- | --- | --- | --- |
| Inouye et al.  (2006).  USA | **Method**  Cross-sectional survey  **Objective**  To describe the Hospital Elder  Life Program  (HELP) across dissemination sites, to detail adaptations, and to summarize advantages  across sites.  **Sample** | Volunteers were provided education sessions and supervision by paid staff about the HELP model. | **Level of intervention-** Large scale, 13 hospitals, model of care.  **Care process change**  The HELP program was implemented at academic teaching hospitals.  The HELP team included The Elder Life Nurse Specialist implemented nursing-related assessments and protocols targeted toward the six delirium risk factors. | **Functional outcome**s  Activities of daily living, falls were assessed. Tool not reported.  **Cognitive outcomes** Cognitive function assessed using Mini  Mental State |

| 11,344 patients  **Setting**  13 HELP sites (academic teaching hospitals) | Volunteers assisted implementation in HELP model interventions. Sites purchased implementation packages (program manuals, business tools, training video tapes/CD, tracking software). There was a help website for their support.  Regular interdisciplinary rounds were available at 69.2% of sites and geriatric consultation at 92.3% of  sites.  **Environmental**  Not described but part of program materials.  **Facilitation**  Elder Life Specialist, Elder Life Nurse  Specialist, and geriatrician. The Elder | Examination  (MMSE).  Delirium assessed  (tool not reported). |
| --- | --- | --- |

|  |  |  | Life Specialist is the program and volunteer coordinator. The Elder Life Nurse Specialist is an advanced practice nurse with specialized training in geriatrics  Regular monitoring and educational sessions for volunteers were available to enhance their performance.  **Discharge planning or care transition planning**  38.5% of sites conducted postdischarge telephone follow up. |  |
| --- | --- | --- | --- | --- |
| Juneau et al.  (2018).  Canada | **Method**  Feasibility study, crosssectional study | Geriatric assessment unit (GAU) professionals (nurses, physicians) received 20 min group training on | **Level of intervention-** small scale, 1 hospital, 1 unit, single focus.  **Care process changes** | **Functional outcomes** Physical dependency  for Activities of Daily Living (ADL) using |

**Objective**	SPRINT prior to study (No more

To assess the feasibility of	details in education component). implementing the SPecific Retraining in INTerdisciplinarity (SPRINT) in the context of a Geriatric

Assessment Unit (GAU).

**Sample**

19 patients

## Setting

Geriatric Assessment Unit of a hospital

SPRINT included four exercise categories according to a level of mobility. The categories range from patient completing exercises alone to requiring professional supervision. Visual and verbal reminders to professionals and encouragement to participants and caregivers. **Facilitation** Physiotherapist and coaches (a registered or ancillary nurse for day shift and a care attendant for evening shift).

Functional

Autonomy

Measurement

System (SMAF)

Fall risk using Berg

Balance scale

## Cognitive outcomes

Cognitive state using

Mini Mental State

Examination (MMSE)

King et al. (2016)	**Method**

UK	Cross-sectional study

**Objective** To determine whether all components of MOVIN (Mobilizing Older adult patients VIa a Nurse-driven intervention) can be implemented simultaneously and to test whether there was a change in nursing practice and unit culture after the intervention on one inpatient unit. To evaluate whether the identified measures were appropriate to detect practice and culture change.

RNs and certified nursing assistants received psychomotor

skills training.

Two PTs (physical therapists) provided psychomotor skills training using an ambulation skills training program that were created by a researcher and a PT collaborator.

**Level of intervention-** small scale, 1 hospital, 1-unit, single focus. **Care process changes** MOVIN mobility program implemented. Communication board and ambulation pathways. Implementation of the mobility aide role occurred on the first day of the intervention.

**Environmental** White boards used to share information on patient care. Visual markers used to measure distances that patients ambulate. **Administrative decisions** Ambulation resources (human and equipment) to maximize opportunity **Functional outcomes** Ambulation frequency (total number of ambulation occurrences on the unit per week), ambulation distance (total distance in feet of patients ambulated on the unit per week) assessed by observation and communication with patient.

|  | **Sample**  18 RNs completed the training; all nursing staff (32 RNs and 10 CNAs) were exposed to the MOVIN intervention; 10 RNs and 5 CNAs agreed to participate in focus groups and 1 RN participated in a single person interview.  **Setting**  25 bed adult general medical unit at a 648-bed tertiary academic teaching hospital. |  | for patient ambulation, and ambulation culture to establish nurse ownership and sustainability of patient ambulation.  **Facilitation**  Mobility aide role. Weekly incentives such as drawings for gifts cards and unit celebrations when ambulation goals were met were implemented. |  |
| --- | --- | --- | --- | --- |
| Kratz et al. (2015)  Germany | **Method**  Pre-post  (Quasi-experimental designs)  **Objective** | A nurse with special training in the management of delirium (“delirium liaison nurse”) carried out an intervention involving | **Level of intervention-** medium scale,  1 hospital, 2 units, model of care.  **Care process changes -** | **Functional outcomes**  **Cognitive**  Mini–Mental State  Examination Score, |

| To answer the following questions: ● What is the incidence of postoperative delirium in a general surgical ward in a general hospital?   - What preoperative factors are predictive of delirium? - Can a specialist geriatric psychiatric nurse reduce the incidence of postoperative delirium by means of non-pharmacological interventions?   **Sample** | component measures of the  Hospital Elder Life Program (HELP) on one of the two wards, with the aim of preventing postoperative delirium. Validation included a training program for nursing staff as a form of self-preservation therapy. | Delirium liaison nurse delivered interventions pre- and postoperatively, according to the individual needs of patients, in the following areas:   - Early mobilization - Improved sensory stimulation ● Improved fluid and nutritional intake - Non-drug sleep improvement - Cognitive activation - Validation   **Environmental**  Lighting to improve sensory stimulation, aids (clocks, calendar, daily newspaper) for reorientation.  **Facilitation** | using NOSGER. Risk  **Physical**  Barthel Index. |
| --- | --- | --- | --- |

N= 125 for the prevalence phase, for the intervention phase N = 114 (61 in intervention group, 53 in control group. **Setting** Two surgical wards or a teaching hospital delivering standard care (both wards underwent the prevalence phase, but during the intervention phase one ward became the intervention group and the other the control group).

Delirium liaison nurse supported delivery of interventions including education for relatives on cognitive activation.

Liu et al. (2018).	**Method**	Study team provided hospitals

Canada	with resources (education **Level of intervention-** medium scale,	**Functional outcomes** 11 hospitals, single focus.

Pragmatic, quasi-experimental interrupted time series (ITS) design.

(Quasi-experimental designs)

**Objective** To implement and evaluate an evidence-based strategy targeting staff to promote early mobilization in older hospitalized patients.

## Sample

12,490 patients (Preintervention: 3,318; during intervention: 2,786 and postintervention: 6,386). **Setting**

modules, checklists, mobility algorithms) to implement the intervention and invited them to use or adapt these, or develop new materials. Local implementation teams at each hospital included a physician, education coordinator, and research coordinator.

## Care process changes

Mobilization of Vulnerable Elders in

Ontario (MOVE ON)

Intervention focused on 1) patients being assessed for mobilization within 24 hours of admission, 2) mobilization 3 times a day, 3) mobility to be progressive and scaled, tailored to patients’ abilities, All hospitals had access to an online community of practice and collaborated in monthly teleconferences. **Facilitation** Local education coordinator, physician champion, and research coordinator facilitated

Patient considered mobilized, if out of bed during visual audit.

|  | 11 University affiliated hospitals |  | implementation of intervention and led/supported the local working group. Implementation coaches worked with local implementation teams to select intervention strategies mapped to identified barriers and facilitators as well as provided support. |  |  |
| --- | --- | --- | --- | --- | --- |
| Martinez-Velilla et al. (2016).  Spain | **Method**  Cohort. Prospective intervention study with blinded outcome progression  (Quasi-experimental designs)  **Objective**  To assess if an exercise intervention involving patients and families could modify the | Ward and research team staff were educated to actively encourage mobility and functional independence (Not explained in detail). | **Level of intervention-** small scale, 1 hospital, 1 unit, single focus.  **Care process changes**  Individualized graduated exercise programs were prescribed and supervised by an exercise physiologist and the unit physiotherapist. Patients and caregivers were educated to | **Functional outcomes**  Dependence in daily activities was assessed using  Barthel index. Nutritional status by the mini-nutritional assessment (MNA). |  |
|  | cognitive and affective progression of hospitalized older patients, from admission to discharge and 30 days after discharge.  **Sample**  29 patients (17: intervention and 12: control).  **Setting**  Geriatric ward of a tertiary hospital | | encourage mobility and functional independence. Participants had to exercise 30-40 min 3 times daily. **Discharge planning or care transition planning**  Participants continued exercise after discharge for 1 month later, recorded in a calendar and reviewed during follow-up medical visit. | **Cognitive outcomes:** Dementia assessed using Global deterioration scale (GDS).  Mental function evaluation using the Spanish version of the Folstein Mini-  Mental State  Examination.  Delirium assessed by the Spanish version | |

of the Delirium

Rating Scale Revised-

98 and Confusion

Assessment Method (CAM).

Depression assessed using Spanish version of the Yesavage abbreviated questionnaire (GDS-

Y)

Cognitive processing speed using Trail

Making Test.

| Milisen et al.  (2001).  Belgium | **Method**  Longitudinal prospective before/after design (Quasiexperimental designs)  **Objective** | Educational poster placed in the emergency department and the traumatological to educate all nurses on assessment, pathophysiology, diagnosis and treatment of delirium, | **Level of intervention-** medium scale,  1 hospital, 2 units, single focus.  **Care process changes**  **Model of Care Change**  An integrated model of geriatric nursing care | **Functional**  **Outcomes:**  Activities of Daily  Living (ADLs) using  Katz Index of |
| --- | --- | --- | --- | --- |

To develop and test the effect of a nurse led interdisciplinary intervention program for delirium on the incidence and course (severity and duration) of delirium, cognitive functioning, functional rehabilitation, mortality, and length of stay in older hipfracture patients.

## Sample

60 patients in intervention cohort and 60 patients in a usual care/non intervention cohort. **Setting**

depression, and dementia. Poster included (1) core symptoms of delirum according to the CAM

(Confusion Assessment Method), (2) comparative features, and differences between delirium, dementia, and depression, and (3) the relevance of correct and early recognition of delirium. All nurses were trained in using the NEECHAM Confusion Scale – screening instrument to assess acute confusion/delirium. Nurses were recruited as resource nurses by a geriatric nurse specialist. Resource nurses were given special training in the for enhanced quality of nursing care for older hip fracture patients. Nurses identified high risk patients and provide prompt anti delirium interventions, provided anti-delirium interventions from a nursing guide for evaluation of causes of delirium in older hospitalized patients (details not given) although regular administration of pain medications (a short acting weak opioid in combination with a nonopioid analgesic such as acetaminophen) was identified. **Facilitation** 8 Specially trained unit-based resource nurses all of whom were Activities of Daily Living.

## Cognitive Outcomes

Delirium using the

English version of the

CAM (Confusion

Assessment Method). Acute confusion using NEECHAM

Confusion scale. Cognitive status using MMSE (Mini

Mental State

Examination).

|  | The emergency department of the university hospitals of Leuven and two traumatological units of an academic medical center | identification and management of older hip-fracture patients at risk for delirium. | prepared at the graduate level. Consultation with a geriatric nurse specialist or a psychogeriatrician if needed. |  |
| --- | --- | --- | --- | --- |
| Miller et al.  (2004).  USA | **Method**  Pre-post, quasi experimental design  (Quasi-experimental designs)  **Objective**  To test the Elder Care  Supportive Interventions Protocol (ECSIP), which was designed to reduce the discomfort of confused, hospitalized | All nursing staff in the participating unit were provided a 2-hour education program. The education program included significance of confusion and discomfort with hospitalized older adults, the purpose and design of the study, and examination of the Elder Care Support Intervention Protocol  (ECSIP). | **Level of intervention-** medium scale,  1 hospital, 2 units, model of care  **Care process change**  Elder Care Support Intervention Protocol (ECSIP) included four components: staff education, implementation team, elder care assistants (ECAs) and environmental props.  Patients’ information (routines of rest and activity, food preferences, diversional activities, personal | **Functional outcome**s Physical function using Katz index of ADL. Discomfort using the modified  Discomfort Screen-  Dementia  Alzheimer’s Type  (DS-DAT)  **Cognitive outcomes** Severity of delirium assessed by |

older adults and the associated negative consequences of delirium, impaired physical function, and increased need for posthospital care; and to foster the interest of undergraduate nursing students in gerontological nursing practice.

**Sample** Pre-intervention: 81 patients

(control: 38 and intervention: 43) and post-intervention: 52 patients (control: 20 and intervention: 32) Nursing staff Undergraduate nursing students received basic undergraduate coursework regarding physical care, assessment of basic needs, patient safety, and therapeutic communication.

history, etc.) obtained from primary	NEECHAM confusion caregivers within 24 hours of	scale.

admission was used to individualize

care protocol. Interventions included prevention and control of discomfort and maintaining a familiar environment with meaningful communication and sensory input. Family caregivers were provided with a thank you for coming booklet to encourage their participation in care

## Environment

Environmental props (no detail provided)

## Facilitation

|  | from the study unit participated.  **Setting**  A 34-bed geriatric medical unit and a 32-bed orthopedic/trauma surgical unit of a tertiary care, academic hospital. |  | Nurse managers and clinical nurse specialists designed and supported staff in implementation. Formal and informal meetings were held re individual patient progression. |  |
| --- | --- | --- | --- | --- |
| Mudge et al.  (2008).  Australia | **Method**  Prospective controlled trial with blinded outcome evaluation  (Quasi-experimental designs)  **Objective**  To evaluate the effect of a structured, multicomponent, | Ward nurses educated on deconditioning risks and benefits of encouraging independence.  Group in-service. | **Level of intervention-** medium scale,  1 hospital, units, model of care  **Care process changes** The multicomponent, early rehabilitation program included: a graduated exercise program prescribed and supervised by the unit physiotherapist and | **Functional outcomes** Change in functional status (admission and discharge) using modified Barthel index (MBI).  Change in mobility  (admission and |

| early rehabilitation program on functional status, delirium, and discharge outcomes of older acute medical inpatients.  **Sample**  124 patients (Intervention group: 62 and control group:  62)  **Setting**  Internal medicine department of a metropolitan, tertiary public teaching hospital | multidisciplinary team staff encouraged mobility and functional independence Cognitive interventions delivered by psychology students supervised by a senior psychologist. Information resources included a walking map of ward and surroundings. Socialization, orientation and memory activities conducted. Daily patient review and grand rounds. Patients provided with diary to record daily activity program  **Facilitation**  Recognition and fostering of clinical champions. | discharge) using the timed up-and-go  test.  **Cognitive outcomes** Delirium identified through chart review. |
| --- | --- | --- |

| Mudge et al.  (2020).  Australia | **Method**  Prospective pre-post (Quasiexperimental designs)  **Objective**  To implement a multidisciplinary comanagement model for older vascular patients and evaluate impact on length of stay (LOS), delirium incidence, functional decline, medical complications and discharge destination.  **Sample**  Pre-intervention: 112 patients and post-intervention: 123 patients. | This study reported that allied health assistants were trained for smooth implementation of “Eat  Walk Engage” program. NOTE Education component described in model protocol/description Mudge et al.  (2015) (cited by authors) | **Level of intervention-** medium scale,  1 hospital, 1 unit, model of care  **Care process change**  Eat Walk Engage Intervention included physician reviewed all patients over 65 years and younger patients with complex comorbidities, prioritized cases based on daily discussion with the nursing team; experienced clinical facilitators implemented the model of care to enhance nutrition and hydration, mobility and meaningful cognitive activities of patients. Daily physician rounds, huddles and weekly discharge planning meetings.  **Environmental** | **Functional outcome**s Functional status measured as the number of basic ADL (bathing, dressing, mobility, transfer, toileting and feeding) documented by  nursing staff.  **Cognitive outcomes** Delirium assessed using Confusion  Assessment Method. |
| --- | --- | --- | --- | --- |

**Setting**	Orientation boards

23-bed vascular surgical ward	**Facilitation** in a publicly funded	Facilitators involved a local metropolitan teaching	multidisciplinary work group hospital.	(MDWG), identified care practices

for older patients, supported

reflection on practice to prioritize improvements; and supported small improvement cycles.

Monthly MDWG meetings were held to encourage team communication and shared goals.

Mudge et al.	**Method**	A nurse or allied health	**Level of intervention-** medium scale,	**Functional outcomes**

(2022)	Cluster randomized CHERISH	professional from within each	4 hospitals, 8 units, model of care	Composite measure

Australia	(Collaboration for Hospitalized	hospital was employed for 2 days	**Care process changes –**	of hospital-

Elders Reducing the Impact of	per week as a site facilitator and	CHERISH (Collaboration for	associated

Stays in Hospital) trial	were trained and mentored by 2	Hospitalized Elders Reducing the	complications of

(RCT)

**Objective** To implement and evaluate a ward-based improvement program (“Eat Walk Engage”) to more consistently deliver age-friendly principles of care to older individuals in acute inpatient wards. **Sample** 539 consecutive inpatients aged 65 years or older, admitted for 3 days or more to study wards. **Setting**

8 acute

experienced facilitators on Eat Walk Engage program –that focused on nutrition and hydration, mobility, meaningful cognitive and social engagement and in multidisciplinary teamwork.

Site facilitators engaged staff to form a multidisciplinary working group (nurse unit manager, nurse educator, physiotherapist, dietitian, and occupational therapist) that reviewed local interview and audit findings to prioritize areas for improvement aligned with key principles and program goals (met for 1 hour Impact of Stays in Hospital), also called the EAT WALK ENGAGE program. Focused on consistent delivery of nutrition and hydration, mobility and cognitive/social engagement as well as interdisciplinary teamwork. Improved the patient lounge as walking destination, maps and signage to assist navigation, and chairs. Nursing assistant shift times changed to support meals. Nurse assistant/PT/OT to assist patients into chairs to eat. Nurses revise workflow at meal time. **Environmental**

To support cognition

older people (HACOP) such as delirium, disability, hospitalassociated incontinence, fall or pressure injury

## Cognitive outcomes

Delirium assessed by Confusion

Assessment Method.

medical and surgical wards in 4	per month and worked with the public hospitals.	facilitator and other staff as

required).

3D-CAM (Confusion Assessment Method) training videos were also provided.

orientation boards and purchases and resources (eg, puzzles, games, pencils, books, glasses, cognitive resource cupboard, daily newspaper). Note: Intervention strategies listed in article referred to as “examples” so may not be comprehensive. **Facilitation** On each ward, a nurse or allied health professional, acted as site facilitator

## Administrative decisions

| Mudge et al.  (2023).  Australia | **Method**  Prospective multi-method implementation evaluation  (Quasi-experimental designs)  **Objective**  To understand how Eat Walk  Engage worked across sites by  (i) describing context, implementation and improvements in each site; (ii) describing and analyzing changes in process measures, (iii) describing how facilitation supported tailored implementation as hypothesised by the underpinning i-PARiHS | Experienced facilitators provided four half-day initial group training sessions for site facilitators. Training included didactic and interactive content based on the i-PARiHS facilitation guide, evidence for age-friendly care principles and the prevention and management of hospitalassociated complications and provision of key readings.  Allied health or nursing assistants (selected by interview) were provided two weeks of training in care of older people, including instruction manual, face-to-face training with local allied health | **Level of intervention-** medium scale,  4 hospitals, 4 units, model of care  **Care process changes**  **Model of Care Change** Eat Walk Engage Programme focused on improving care of older people.  Allied health or nursing assistants set patients up for meals, supervised mobility or exercise activities, provided assisted listening devices, or updated orientation boards.  **Environmental**  Orientation boards  **Administrative decisions** Project funding supported 24 hour/week experienced facilitator | **Functional outcomes** Patients were assessed for eating patterns by crosssectional structured mealtime observations. Observers recorded whether patients required assistance with meal set-up or eating, and whether they received this assistance within 10 minutes.  Observer also noted whether each |
| --- | --- | --- | --- | --- |

implementation framework and (iii) proposing how key contextual features of different sites may have contributed to implementation success or failure.

## Sample

In-patients on the four intervention wards, and multidisciplinary work group members and facilitators. **Setting** Four hospitals (2 inner metropolitan, 1 outer metropolitan, 1 regional) professionals (e.g., physiotherapist, speech pathologist) and work shadowing with an experienced assistant. time. to support project management and external

facilitation across sites. In addition, 16 hour/week site facilitator plus 20 hour/week multi-professional assistant for each implementation ward.

## Facilitation

Mentoring included monthly halfday face-to-face peer group meetings. Telephone and email support were provided between meetings, supporting debriefing, reflection on practice and shared learning.

Experienced facilitators visited. patient was sitting up when the meal arrived, had the tray table in reach, and was interrupted during the meal. Percentage of mealtimes when patients were sitting in a chair and received timely assistance were evaluated.

**Cognitive outcomes** Patients were observed systematically for

two minutes before moving to the next room in continuous sequential observations between 8 am and 4 pm. The highest level of activity during each observation period was recorded along with physical activity, cognitive/social activity, and

company. Percentage of observations where patient was standing or walking and the percentage where they were engaged in cognitive or social functioning (e.g., talking to others, reading, watching television) were evaluated.

Naylor et al.	**Method**	Education/training component	**Level of intervention-** medium scale,	**Functional outcomes**

(2014)	Controlled trial. Prospective	was present only in Resource	3 hospitals, units, model of care	Functional status USA	comparative effectiveness	Nurse Care (RNC) or Transitional	**Care process changes**	(patient’s ability to

study	Care Model (TCM) interventions.	One of the three interventions ASC,	conduct self-care (Quasi-experimental designs)	For RNC intervention, hospital	RNC and TCM were randomly	[BADL] and

**Objective**	employed RNs completed a web-	assigned to one of three hospitals.	instrumental

To report the effects of three evidence-based interventions of varying intensity, each designed to improve outcomes of hospitalized cognitively impaired older adults. **Sample**

202 older patients (Augmented

Standard Care (ASC): 65; Resource Nurse Care (RNC): 71 and Transitional Care Model [TCM]: 66)

**Setting** 3 hospitals within an academic health system.

based module developed by the study team in consultation with clinical experts prior to the intervention. The module focused on managing and transitioning hospitalized cognitively impaired older adults. They also attended seminars on issues relevant to cognitive impairment.

For TCM intervention, Advanced Practice Nurses (APNs) completed orientation designed for resource nurses, along with TCM specific web-based modules and

individualized clinical experiences.

All interventions included screening, reporting and documenting cognitive

deficits.

RNC conducted cognitive screening within 24 hours of hospitalization. Findings were communicated to the interdisciplinary team within 2 hours. TCM protocol consisted of hospital and home visits by advanced practice nurses (APN), physician follow-up, telephone outreach and supplemented care of hospitalized patients (not defined) and APNs substituted for RN home care. activities of daily living [IADL], i.e., patient’s ability to perform higher **l**evel activities such as using the telephone, medication management, household chores) were collected by inperson interviews with caregivers.

Peyrusque et al.	**Method**

(2021)	Cross-sectional study. Single

Canada	arm interventional pragmatic

pilot study

**Objective** To assess the effect of a pragmatic, unsupervised, and specific PA program (SPRINT) on health care practice and functional capacities in hospitalized older patients

## Sample

19 patients enrolled in SPRINT program

RNC intervention: Trained RNs coached other nurses involved in patient care. All GAU professionals (nurses, physicians and physiotherapists) received a 20-minute group training on SPRINT prior to study. Note education previously described in Juneau et al. (2018).

**Level of intervention-** small scale, 1 hospital, 1-unit, single focus. **Care process changes** SPRINT included four exercise categories according to a level of mobility. The categories range from patient completing exercises alone to requiring professional supervision. Visual and verbal reminders to professionals and encouragement to participants and caregivers. Patients wore physical activity monitoring using accelerometers.

**Functional outcomes** Ability of the patient to complete 7 activities of daily living (ADL) (eating, washing, dressing, grooming, urinary function, bowel function and toileting) assessed using the Functional

Autonomy

Measurement

System (SMAF).

## Setting

Geriatric Assessment Unit

(GAU)

Rodrigues et al.	**Method**

(2020).	2-group randomized controlled

Portugal	trial (RCT)

**Objective** To test the hypothesis that a nursing care program focused on basic self-care (N_BSC) improves functional outcomes in older patients admitted to an acute medical unit.

All nurses and nursing assistants were educated on N_BSC. The N_BSC program was inspired by the Eat Walk Engage model and Function-focused Care Philosophy. Program focused on improving daily walking, trips to the toilet by walking (with support devices or with people support) and all daytime meals seated (out of bed). Education **Level of intervention-** medium scale,

1 hospital, 1 unit, model

**Care process changes** N_BSC program involved changing physical structures of the service, reorganizing the nursing care, and coaching nurses and nursing assistants. Nurses encouraged and assisted patients to eat/walk or toilet use, ensure patients sitting ready to eat and promoted self-care, Mobility was evaluated using the Physiotherapy

Functional Mobile

Profile (PFMP) tool.

## Functional outcomes

**Functional status** (Bathing, dressing, eating, and toileting [BADL]) were assessed using Katz index. Functional status of patients was assessed for 2 weeks prior to admission

| **Sample**  182 older adults (Intervention group: 91 and usual care group: 91)  **Setting**  580-bedded teaching hospital | was provided prior to the intervention. | walk destinations, and minimized clinical activities during meal times.  **Environmental**  A 30-meter blue line along with handrail in the central corridor for walking support.  Ambulation pathways with distance markers. Hospital furniture rearranged to eliminate obstacles. Social and dining room. Television, sofa, games, magazines/ newspapers available. 2 hand-held carts to carry portable oxygen cylinder.  **Administrative decisions** | (based on selfreport), at admission (observation based) and at discharge  (nursing evaluation). |
| --- | --- | --- | --- |

| Rubin et al. (2011)  USA | **Method**  Not reported (? cohort study)  (Quasi-experimental designs)  **Objective**  To describe the evolution of the HELP program at Shadyside over the 7-year period from 2002–2008 (inclusive), including adaptations, patient outcomes, cost savings, challenges and successes.  **Sample**  Over 7000 Patients aged 70 and older who met the HELP  criteria. **Setting** | Volunteers received a complete training program. Some volunteers were trained by speech therapists in an enhanced feeding protocol, while some were trained by physical therapists in an enhanced mobilization protocol. Measured staff knowledge at baseline and re-assessed after 6 months, implies that staff may have received training/education) Volunteer trainings were fully standardized and welldocumented. | **Level of intervention-** medium scale,  1 hospital, 6 units, model  **Care process changes**  HELP model included staffing, operational structure, interventions, and quality assurance. The care processes from Inouye et al. 2000 & 2006. Sleep protocol was modified to include hand rather than back massage.  **Environmental**  Unit-wide noise reduction strategies were already being implemented prior to HELP.  **Administrative decisions** Hospital leaders agreed to continuously fund the HELP and | **Cognitive outcomes**  Delirium assessed by  Confusion  Assessment Method. |
| --- | --- | --- | --- | --- |

500 bed community teaching	Note education previously

hospital	described in Inouye et al. (1993a

& 2000).

allowed the HELP program to expand to additional units. **Facilitation**

Weekly meetings were held with Hospital Elder Life Program Staff and monthly meetings with the program

Suwanpasu et al.	**Method**	The registered nurses (RNs) who

(2015)	volunteered to become GRNs, director and a process improvement

specialist.

Elder Life Specialist was designated as the ‘lead volunteer coordinator’ to provide front-line supervision and coordination to volunteers. A bachelor-prepared nurse was enrolled so as to allow nurse specialist to extend her scope.

## Level of intervention- medium scale, Functional outcomes

1 hospital,

Thailand	A pre-post test design without

a control group

(Quasi-experimental designs)

**Objective** To assess the effectiveness of a geriatric resource nurse (GRN) programme in maintaining the functional status of elderly patients hospitalized for community-acquired pneumonia (CAP). **Sample** 15 men and 8 women (N=23) with CAP and admitted to the hospital between July 2014 and December 2014.

## Setting

had worked with elderly patients for at least 5 years and possessed the knowledge, skills, and ability to improve care for hospitalized elders. GRNs were trained to be the frontline experts to assess elderly patients and to consult with a geriatric advanced practice nurse with expertise in handling more complex cases involving elders at high risk. Geriatric core curriculum included contents such as: geriatric syndromes, geriatric specific disease entities, multidimensional assessment of the older adult (SPICCIES tool),

1 unit, model

## Care process changes

Used Nurses Improving Care for Health System Elders (NICHE) model to train geriatric resource nurses. Focused on geriatric syndromes, using the geriatric core curriculum protocols. Unit-based clinical rounds with a geriatric advanced practice nurse were held twice a week. Multidisciplinary advisory team including physician, nursing administrator, physiotherapist, and pharmacist was formed.

**Facilitation** Geriatric advanced practice nurses supported the GRNs to distinguish The SPICES tool was used to assess patients’ sleep disorder, problems with eating or feeding, incontinence, confusion, immobility, evidence of falls, and skin breakdown. Sleep disorder assessed using the

Verran and SynderHalpern Sleep Scale. Problems with eating or feeding are

King Chulalongkorn Memorial	and best practice protocol.

hospital	SPICCIES assessed sleep disorder,

problem with eating or feeding, incontinence, confusion, caregiver preparedness, immobility, evidence of falls, and skin breakdown.

risk factors and to make recommendations, physical assessment skills, and to encourage communication between health care teams, patients and families. GRNs were also provided with relevant journal articles as support for recommendations. GRNS were continuously mentored

to improve their skills.

evaluated using the Edinburgh Feeding

Evaluation in

Dementia

Questionnaire. The immobility assessment tool is based on the NANDA statement: “immobility is the individual experiencing a limitation of ability for independent physical movement”. Evidence of falls evaluated using the

Hendrich II Fall Risk Model. Skin breakdown is evaluated using the Braden Scale for

Predicting Pressure Sore Risk.

**Cognitive outcomes** Confusion is assessed using the

Thai version of the

Confusion Method Assessment algorithm, based on the Diagnostic and

Statistical Manual of

Vidan et al. (2009)	**Method**

Spain	Prospective controlled clinical

trial

(Quasi-experimental designs)

## Objective

To analyze the effectiveness of a multicomponent intervention integrated into daily practice for the prevention of in-hospital delirium in elderly patients.

## Sample

542 patients were included

(170 patients in the GI

Briefly mentioned that education was targeted to staff to change the approach of patient care, but not discussed in detail. Educational session explaining the characteristics of delirium, recognition of delirium and risk factors were given by senior geriatrician and specialist geriatric nurse. Poster with environmental and general prevention measures such as avoiding noise, stimulating mobilization and hydration, advice about speaking **Level of intervention-** medium scale,

1 hospital, 1 unit, model

**Care process changes** Intervention included staff education and specific targeted delirium risk factors – hydration and nutrition, orientation, sensory perception (glasses and hearing aids), sleep preservation, mobilization, and a drug list review.

**Environmental**

Clocks and calendars in every room.

## Administrative decisions

Mental Disorders criteria for delirium.

**Functional outcomes** Functional decline calculated as the proportion of participants whose performance on

activities of daily living (ADLs) was worse at discharge than at baseline.

ADLs assessed by Katz index of activities of daily living. The ability to walk was defined

|  | [Geriatric Unit] group and 372 in the UC [Usual Care] group)  **Setting**  University hospital | to the patient were kept in nurses’ station.  Nurses used cards with recommendations included in the first page of the treatment book. | Avoid physical restraints. Used only with medical authorization and signature of physician.  **Facilitation**  Geriatrician and Specialist Geriatric  Nurse | according to the  Functional  Ambulation  Classification.  **Cognitive outcomes** Cognitive function assessed by Spanish version of the  Folstein Mini-Mental  State Examination (MMSE). Delirium assessed by  Confusion  Assessment Method. |
| --- | --- | --- | --- | --- |
| von Renteln-Kruse  & Krause (2007).  Germany. | **Method**  Prospective cohort with historical control study. | Nurses, therapeutic staff, and physicians were given an hourlong presentation fall prevention | **Level of intervention-** small scale, 1 hospital,  1 unit, single focus | **Functional outcome**s  Activities of daily living were assessed |

(Quasi-experimental designs)

**Objective** To evaluate the effect of an interdisciplinary team approach on reducing the number of falls in geriatric hospital inpatients by comparing long periods before and after introduction of the intervention.

## Sample

Before intervention: 4,272 patients and After intervention: 2,982 patients. **Setting**

measures. Participants were trained in supporting patient transfer, fall-risk assessment tool and the checklists to be used on the wards. Patients at risk and their relatives/caregivers were provided individual education about fall risk and preventive measures, including behavioral changes. Four half-day training seminars and refresher courses on patient handling were held per year. Other routinely available measures were: training in ADLs, balance and gait, transfer, safe **Care process change** Fall prevention included assessment for fall-risk within 48 hours of admission. If a risk, a visible ‘risk alert’ sign was placed above the patient’s bed. 5-page flyer for patients and families about typical risks and in hospital preventative measures (footwear, eyeglasses, hearing aids, hip protectors and mobility devices) instructions on getting out of bed, fluid intake and communications. Patients in need of frequent toileting were provided.

using the Barthel Index. Fall risk was assessed using the St.

Thomas’s Risk

Assessment Tool in

Falling Elderly

Inpatients

(STRATIFY).

|  | Geriatric clinic of an academic teaching hospital | use of devices (occupational therapists, nurses). | Patients were encouraged to get out of bed as early as possible and to sit on normal chairs when eating meals. additional assistance or supervision.  **Environmental**  A visible ‘risk alert’ sign was placed above the patient’s bed if they were identified at risk on assessment. Mobility devices were provided immediately upon admission if it was determined necessary. Commodes were provided |  |
| --- | --- | --- | --- | --- |
| Wand et al.  (2014).  Australia | **Method**  A before and after study  (Quasi-experimental designs)  **Objective** | One-hour education sessions were organized as part of the routine education program for Nursing and medical staff. Nurse educator conducted education | **Level of intervention-** medium scale,  1 hospital,  1 unit, model  **Care process changes** | **Functional outcomes** Overall functioning measured by the  Barthel activities of daily living index. |

To evaluate the effectiveness of a multifaceted educational program in preventing delirium in hospitalized older patients and improving staff practice, knowledge and confidence. **Sample**

255 patients of age 65 years and older (126: preintervention and 129: postintervention) 77 staff (45 prior to the educational intervention and 32 after intervention). 35 were nurses and 39 doctors. **Setting**

sessions for nurses and psychogeriatrician and geriatrician conducted sessions for medical staff. Educational content included general information on delirium (definition, risk factors for developing delirium, symptoms, epidemiology, differential diagnosis and outcomes), prevention (detailed discussion of how to address potentially modifiable risk factors), and management of patients with delirium (early detection, legal framework of care, nonInterventions included addressing bladder and bowel function (constipation, urinary retention), hydration, pain, nutrition, sensory aides and communication, remove attachments (catheters, IVs, and catheters), oxygenation, sleep, medication review, management of drug dependence. **Environmental** Ward environment was optimized to make it elder friendly. Examples include large-face clocks in patient rooms and large picture-based signs indicating toilets.

## Administrative decisions

Avoid use of physical restraints.

**Cognitive outcomes** Cognitive impairment assessed using Mini-Mental

Status examination. Dementia assessed using the Blessed dementia scale and clock-draw test. Delirium assessed by confusion assessment method. Barthel’s score from admission to discharge in the post intervention group.

|  | 22-bed general medical ward of a district hospital | pharmacological and pharmacological management). Weekly tutorials were provided on the study ward and one-page summary of the key points of each education session. Posters were displayed in prominent areas of the study ward reinforcing these key points. | **Facilitation**  Local champions for delirium prevention were identified as the delirium resource staff, a Nurse  Educator and senior geriatrician  (WT)  Weekly tutorials with these delirium resource staff. |  |
| --- | --- | --- | --- | --- |
| Wang et al.  (2020).  China | **Method**  2-arm parallel-group, singleblind, cluster randomized  clinical trial  (RCT)  **Objective** | Project director provided intensive training, including theoretical knowledge and assessment skills for delirium to all the assessors. Training included a review of the t-HELP procedure outlined in a short booklet and video. | **Level of intervention-** medium scale,  1 hospital, 6-unit model  **Care process changes**  The interdisciplinary t-HELP (tailored for family involvement instead of volunteers from the hospital elder life program adapted from the HELP program) Intervention included 3 | **Functional outcomes** Changes in physical function before and after the surgical procedures measured using the Barthel index and instrumental |

| To investigate the effectiveness of the Tailored,  Family-Involved Hospital Elder Life Program (t-HELP) for preventing postoperative delirium (POD) and functional decline in older patients after a noncardiac surgical procedure.  **Sample**  281 patients (Intervention/thelp group: 152 and usual care group/control group: 129)  **Setting**  24 nursing units across 6 surgical floors of hospital | Medical postgraduates, majoring in geriatrics or geriatric nursing were trained as coordinators, who took charge of delivering the intervention plan to nurses, surgical doctors and other team  staff. | universal protocols and 8 targeted protocols. Universal protocols included orientation, therapeutic activities, and early mobilization. Targeted protocols were implemented according to the presence of delirium-related risk factors and included pain management, sleep enhancement, nutritional assessment, fluid repletion/constipation management, etc. Control patients in a usual care unit received the usual treatment and care, without individualized assessment or interdisciplinary intervention. | activities of daily living (IADLs).  **Cognitive outcomes** Postoperative delirium assessed using CAM  (Confusion  Assessment Method) Incidence of severe delirium during hospitalization was measured by the  Chinese version of  MDAS (Memorial  Delirium Assessment  Scale) |
| --- | --- | --- | --- |

|  |  |  | Nurses screened for delirium risk factors. Geriatric specialists develop individualized intervention plan based on delirium risk factors.  Intervention daily after surgery. Patients provided with information booklets with information about interdisciplinary interventions, pain alleviation, sleep improvement, and relaxation skills.  **Facilitation**  Coordinators assisted nurses organizing the interdisciplinary consultation twice a week to discuss special cases | Change in cognitive function was assessed by the Chinese version of the SPMSQ (Short  Portable Mental  Status  Questionnaire). |
| --- | --- | --- | --- | --- |
| Wanich et al.  (1992) | **Method**  Quasi-experimental design | Two geriatric clinical specialists gave an in-service program to | **Level of intervention-** medium scale,  1 hospital, 1 unit, model | **Functional outcomes** |

USA	**Objective**	nursing staff that included mental	**Care process changes**	Functional status

To examine the effectiveness	and functional status	Nurse specialists provided	assessed by Katz

of a nursing intervention for	assessments, gerontological	orientation cues to patients (e.g. day	Index of Activities of

elderly	nursing management of deficits	of the week, current events, a	Daily Living.

hospitalized patients as	in sensory-perceptual function,	discussion of their condition and	**Cognitive outcomes** measured by functional	mobility and environmental	information about upcoming	Cognitive deficits outcomes	modifications. Educational	diagnostic or therapeutic measures).	assessed by Mini-

**Sample**	sessions were repeated once	Families and nursing staff were	Mental State

235 hospitalized patients (135	during the course of the study.	taught to communicate clearly and	Examination.

in intervention group and 100	slowly with patients and use	Delirium diagnosed in control group) aged 70 years	repetition and orientation cues.	by a study and older, admitted between	Patients mobilized by nursing staff	psychiatrist based on

Sunday noon and Friday noon.	out of bed each day, ambulated,	DSM-III (Diagnostic &

**Setting**	taken out of their rooms and	Statistical Manual,

700-bed urban, teaching	consulted physical and occupational	third edition)

hospital affiliated with schools	therapy.	criteria.

of nursing and medicine.

Families were asked to visit

frequently or call daily; bring in photos and personal mementos from home to assist in orientation and personalizing the environment.

Patients' medications reviewed daily. Medications suspected as contributing to delirium evaluated critically on an individual basis, use discouraged. Families asked to make glasses and hearing aids available and nursing staff encouraged to use them. Patients and families asked about preferences (favorite television programs, news channels and radio stations) to assist with stimulation and orientation.

**Environmental** Environmental modifications were done to meet the needs of hearing or vision impaired patients (details not given). Updated calendars were placed in every room. Lighting was used to decrease sensory deprivation. Night lights were turned on at dusk and left on until morning. **Facilitation** Nurse specialists held frequent informal discussions with the

staff.

## Discharge planning/transition care

A Geriatric Clinical Nurse Specialist (GCS) reviewed patient’s functional and cognitive status weekly with the

|  |  |  | discharge planning team, including primary nurse, a social worker, a discharge planning nurse, a physical and occupational therapist and a nutritionist. |  |
| --- | --- | --- | --- | --- |
| Zisberg et al.  (2018).  Israel | **Method**  Prospective cohort study  (Quasi-experimental designs)  **Objective**  To demonstrate the process of adapting a human factors framework, the Systems  Engineering  Initiative for Patient Safety (SEIPS 2.0)], as a guided model to articulate a site-specific, culturally based intervention | Staff were provided online tutorials and face to face training by PTs to enhance their knowledge. | **Level of intervention-** small scale, 1 hospital,  2 units, single focus  **Care process changes**  The Systems Engineering Initiative for Patient Safety (SEIPS) 2.0 model, development of WALKFFOR model.  Interdisciplinary protocols for mobility  Bilingual patient/family resources (brochures, posters) on maintaining mobility. | **Functional outcomes**  In-hospital mobility (900 steps and above considered as mobility level). |

to improve in-hospital mobility in older adults.

## Sample

203 older adults hospitalized in two internal medicine units at an academic medical center. 116 medical staff, including nurses, nurse’s aides (NAs), physical therapists (PTs), and medical doctors (MDs) **Setting**

Academic medical center **Environmental**

Walking trails,

## Administrative decisions

Extra walkers purchased Electronic reports on mobility, mandatory reporting on mobility

## Facilitation

Head nurse led team meetings to discuss interventions, workflow. Daily review of EMRs, weekly discussion of protocol implementation.

612 **___________________________________________________________________________________________________________**
613 Table 2.

# 614 *Details on Focus of Educational Strategies Utilized by Studies*

S. N First	HCPs included in	Topics covered.	Education author	education	(e.g., delirium, mobility)	strategies used

(Year)	(e.g., in-service,

workshops, pockets cards)

1. Allegri et Yes: Staff (doctors, Dementia, cognitive Training using al. (2022) nurses, physical impairment, depression teaching modules.

therapists and	bipolar psychotic

healthcare assistants).	disorder, delirium, pain,

communication and interaction with patients

2. Apolinario	Yes (nurses,	Fall, pressure injury,	Education targeted

et al.	physiotherapist, speech bronchoaspiration, delirium,	to train

(2022)	therapist, nutritionist,	mechanical restraint,	professionals from

psychologist, and malnutrition, medication different areas. clinical iatrogenesis, and functional Staff were given pharmacologist). decline. resources such as

booklets, video

lessons, infographics, onsite small group training, and training workshops.

| 3. | Bakker et al. (2014). | Yes (Nurses and Physicians). | Delirium, physical decline, fall, frailty screening, medical review, geriatric assessment, stimulation of cognitive and physical activities. | Educational session, on the job coaching. |
| --- | --- | --- | --- | --- |
| 4. | Boltz et al.  (2014) | Yes (Nurses). | Prevention and management of delirium and functional decline; assessment of mood, cognition, and function; incorporation of function-focused care into care routines; ways to communicate, partnering, and motivating patients and family; and discharge planning. | Education session. |

5. Bryant et Yes (nurses, ancillary Delirium prevention, Educational al. (2019) staff, residents, mobility, medication presentation,

physician assistants management/prognostic pocket card, and attending staff assessment, reducing other mandatory who frequently care complications, social educational for older patients in needs/goal setting and injury modules. trauma unit and in the prevention.

ICU).

6. Chang et al Yes (nurses).	Mobility.	In-service.

(2007)

7. Cohen et al Yes (Nurses, nurse	Mobility.	Theoretical and

(2019)	aides).	practical training.

8. Gazineo et	Yes (only one nurse).	Mobility.	Training.

al. (2021)

9. Gill et al	Yes (unit staff	Mobility.	Theoretical and

(2023)	including nurses, nurse	practical training.

aides and physical

therapists).

10. Hamilton	Yes (nurses).	Mobility.	Staff education.

& Lyon

(1995)

11. Heim et al.	Yes (nurses and	Frailty, ADL functioning.	On the job

(2016).	medical staff).	training.

12. Holt et al.	Yes (ward staff	Delirium.	Interactive lecture,

(2013)	including staff nurses	handout, quiz, and healthcare	poster, reference assistants).	materials and case

vignettes.

13 Inouye et Yes (Nurses). Geriatric care (Delirium, Education, al. (1993b) functional impairment, training, lecture

incontinence and pressure	series.

sores).

14. Inouye et	Yes (Research and	Delirium risk factors:	Training.

al. (1999)	clinical nurses).	cognitive impairment, sleep

deprivation, immobility, visual impairment, hearing impairment and dehydration.

15. Inouye et	Yes (Nurses and	Mobility, vision/hearing,	Formal didactic al. (2000)	Physicians) and	volume repletion, feeding	sessions, small

volunteers.	assistance, sleep.	group training, and

resource materials.

16. Inouye et	Unsure (volunteers).	Delirium.	Regular education

al. (2006)	sessions

17. Juneau et Yes (Nurses, Mobility. Training on al. (2018) physicians and exercise program.

physiotherapist).

18. King et al.	Yes (Nurses and	Mobility.	Training.

(2016).	certified nursing

assistants).

19. Kratz et al.	Yes (Nurses).	Delirium.	Training.

(2015)

20. Liu et al.	Yes (Physicians,	Mobility.	Education

(2018)	nurses).	modules.

21. Martinez-	Yes (ward and	Mobility and functional	Education.

Velila et	research team staff).	independence.

al. (2016)

22. Milisen et	Yes (Nurses).	Delirium, depression, and	Educational

al. (2001).	dementia.	poster, training

23. Miller et	Yes (Nurses and	Confusion, discomfort,	Education

al. (2004)	undergraduate nursing	physical care, assessment of	program.

students).	basic needs, patient safety

and therapeutic communications.

24. Mudge et	Yes (Nurses).	Functional independence	Education, group

al. (2008)	and mobility	in-services

25. Mudge et	Yes (Nurses or allied	Nutrition and hydration,	Training, videos.

al. (2022).	health professional).	mobility, meaningful

cognitive and social engagement and in multidisciplinary teamwork.

26. Mudge et	Yes (allied health	Nutrition and hydration,	Training.

al. (2020).	assistants).	mobility, meaningful

cognitive and social engagement and in multidisciplinary teamwork.

27. Mudge et	Yes (allied health or	Nutrition and hydration,	Training including al. (2023)	nursing assistants).	mobility, meaningful	didactic and

cognitive and social	interactive engagement and in	content.

multidisciplinary teamwork.

28. Naylor et	Yes (nurses and	Cognitive deficit.	Web based

al. (2014).	advanced practice	modules,

nurses).	seminars.

29. Peyrusque	Yes (nurses,	Mobility.	Group training.

et al.	physicians,

(2021)	physiotherapists).

30. Rodrigues	Yes (nurses and	Mobility, nutrition.	Training and

et al.	nursing assistants).	regular education

(2020).	sessions.

31. Rubin et	Unsure (Volunteers).	Nutrition, delirium, overall	Training program.

al. (2011)	care of frail older adults.

32. Suwanpasu Yes (nurses).	Sleep disorder, problem with	Training program.

et al.	eating or feeding,

(2015)	incontinence, confusion,

mobility, falls, and skin breakdown.

33. Vidan et	Yes (nurses).	Delirium.	Educational

al. (2009)	session, poster,

cards with recommendations included in the first page of the treatment book.

34. von	Yes (Nurses,	Mobility.	Presentations,

Renteln-	therapeutic staff, and	training.

Kruse &	physicians).

Krause

(2007).

35. Wand et	Yes (nurses, medical	Delirium.	Ward based in-

al. (2014).	staff).	services,

workshops,

tutorials, education session, posters.

36. Wang et	Yes (Medical	Delirium.	Training.

al. (2020)	postgraduates,

majoring in geriatrics or geriatric nursing were trained as coordinators, who took charge of delivering the intervention plan to nurses, surgical doctors and other team

staff).

37. Wanich et	Yes (Nurses).	Delirium.	In-service

al. (1992)	program.

38. Zisberg et	Yes (Nurses, nurse	Mobility.	Online tutorials,

al. (2018).	aides).	face to face

training.

615

616 Table 4.

# 617 *Interventions in Addition to Education*

| **Study** | **Level of intervent ion** | | | **Care process changes**  (Nurse only (N); Interprofessional (IP)) | | | | | | | | | | | | | | **Environment changes** | | | | | **Administrative**  **Support** | | | | | | **Facilitation/Leadership** | | | | | | **Discharge/**  **Transition**  **Planning** | | | | **Functional outcomes improveme**  **nt** |
| --- | --- | --- | --- | --- | --- | --- | --- | --- | --- | --- | --- | --- | --- | --- | --- | --- | --- | --- | --- | --- | --- | --- | --- | --- | --- | --- | --- | --- | --- | --- | --- | --- | --- | --- | --- | --- | --- | --- | --- |
|  | Large | Medium | Small | Multifocal geriatric protocols | Mobility interventions | Family involvement | Cognitive  interventions | Continence interventions | Pain interventions | Medication review/protocol | Pressure ulcer prevention | Nutrition/Dehydration | Sleep protocol | Physical Function/Falls | Geriatric/Frailty screening | Geriatric patient rounds | Volunteers | Walking trails | Ori  entation (clocks) | Modifications/Declutter | Adjusting seating/bed height | Sensory and mobility aids | Equipment purchase | Additional staffing | Restraint use/policy | Patient/family in shift report | Electronic record modifications | Regional Intersectoral | Advanced  practice nurse | Geriatric resource nurse/staff | Nurse supervisor/case manager | Geriatrician | IP resource person | IP geriatric team | IP family meetings | Links to discharge settings | Minimum data set/digital info | Pati  ent follow up |  |
| Allegri et al. (2022) |  | ***** |  | N |  |  | N |  | N | N |  |  |  |  |  |  |  |  |  |  |  |  |  |  |  |  |  |  |  |  |  |  |  |  |  |  |  |  | **Physical**  Maintained  **Cognitive**  No |
| Apolinari  o et al.  (2022) |  | ***** |  | I  P |  | I  P |  |  |  |  |  |  |  |  | * | I  P |  |  |  |  |  |  |  |  |  |  |  |  |  |  | * |  |  |  |  | * |  |  | **Physical:**  ADL - Yes |

| Bakker et al. (2014). |  | ***** |  | I  P |  |  | * |  |  | * |  |  | * | * | * | * | * |  |  |  |  |  |  |  |  |  |  |  | * |  |  | * |  |  | * |  |  | * | **Physical**  ADL No  **Cognitive**  No |
| --- | --- | --- | --- | --- | --- | --- | --- | --- | --- | --- | --- | --- | --- | --- | --- | --- | --- | --- | --- | --- | --- | --- | --- | --- | --- | --- | --- | --- | --- | --- | --- | --- | --- | --- | --- | --- | --- | --- | --- |
| Boltz et al. (2014) |  | ***** |  | N |  | * |  |  |  |  |  |  |  |  |  |  |  | * | * |  | * |  |  |  |  | * |  |  |  | * | * |  |  |  |  |  |  | * | **Physical:** ADL/Mobili  ty Yes  **Physical:**  Gait and Balance No  **Cognitive:** Delirium severity  Yes |
| Bryant et al. (2019) |  | ***** |  | I  P | ***** |  | ***** |  |  | ***** |  | ***** |  | ***** | ***** | ***** |  |  |  |  |  |  |  |  |  |  |  |  |  | * |  |  |  |  | * |  |  |  | **Cognitive:** delirium  Yes |
| Chang et al (2007) |  | ***** |  | N |  |  |  |  |  | * |  |  |  |  |  |  |  |  |  | * |  |  |  |  |  |  |  |  |  | * |  | * |  |  |  |  |  |  | **Physical:**  ADL Yes |
| Cohen et al. (2019). |  |  | ***** |  | N | * |  |  |  |  |  |  |  |  |  |  |  | * |  | * |  |  | * |  |  |  | * |  |  |  |  |  |  |  |  |  |  |  | **Physical:** |

|  |  |  |  |  |  |  |  |  |  |  |  |  |  |  |  |  |  |  |  |  |  |  |  |  |  |  |  |  |  |  |  |  |  |  |  |  |  |  | BADL No  IADL No |
| --- | --- | --- | --- | --- | --- | --- | --- | --- | --- | --- | --- | --- | --- | --- | --- | --- | --- | --- | --- | --- | --- | --- | --- | --- | --- | --- | --- | --- | --- | --- | --- | --- | --- | --- | --- | --- | --- | --- | --- |
| Gazineo et al. (2021) |  |  | ***** |  | N | * |  |  |  |  |  |  |  |  |  |  |  |  |  |  |  |  |  |  |  |  |  |  |  | * |  |  |  |  |  |  |  |  | **Physical:**  Mobility  Yes |
| Gill et al  (2023) |  |  | ***** |  | I  P |  |  |  |  |  |  |  |  |  |  |  |  | * |  | * |  |  | * |  |  |  |  |  |  |  |  |  |  |  |  |  |  |  | **Physical:**  Mobility  Yes |
| Hamilton  & Lyon  (1995) |  |  | ***** |  | N |  |  | N |  |  |  |  |  |  |  |  |  |  |  |  |  |  |  | * |  |  |  |  | * |  | * |  |  |  |  |  |  |  | **Physical:**  ADL Yes |
| Heim et al. (2016). | * |  |  | I  P |  |  | * |  |  |  |  | * |  | * | * | * |  |  |  |  |  |  |  |  |  |  |  | * |  | * |  |  |  |  |  | * | * |  | **Physical:** ADL Yes (composite outcome) |
| Holt et al.  (2013) |  |  | ***** |  |  |  | N |  |  |  |  |  |  |  |  |  |  |  |  |  |  |  |  |  |  |  |  |  | * | * | * | * |  |  |  |  |  |  | **Cognitive -**  Delirium  Yes |

| Inouye et  al.  (1993b) |  | ***** |  | N |  |  | * | * |  |  | * | * | * | * |  |  |  |  |  |  |  |  |  |  |  |  |  |  | * | * |  | * |  |  |  |  |  |  | **Physical:**  ADL Yes |
| --- | --- | --- | --- | --- | --- | --- | --- | --- | --- | --- | --- | --- | --- | --- | --- | --- | --- | --- | --- | --- | --- | --- | --- | --- | --- | --- | --- | --- | --- | --- | --- | --- | --- | --- | --- | --- | --- | --- | --- |
| Inouye et al. (1999) |  | ***** |  | I  P | * |  | * |  |  |  |  | * | * |  |  |  | * |  | * | * |  |  | * |  | * |  |  |  | * |  |  | * | * | * |  |  |  |  | **Physical**  ADL- yes  (trend)  **Cognitive**  Delirium  Yes |
| Inouye et al. (2000) |  | * |  | I  P | * | * | * |  |  |  |  | * | * |  |  | * | * |  | * | * |  |  |  |  |  |  |  |  | * |  | * | * | * |  |  | * | * | * | **Physical**  ADL Yes  **Cognitive**  Delirium  Yes |
| Inouye et al. (2006) | * |  |  | I  P | * |  | * |  |  |  |  | * | * |  |  |  | * |  | * | * |  |  | * |  | * |  |  |  | * |  |  | * | * |  |  |  |  | * | **Physical**  ADL Yes |

|  |  |  |  |  |  |  |  |  |  |  |  |  |  |  |  |  |  |  |  |  |  |  |  |  |  |  |  |  |  |  |  |  |  |  |  |  |  |  | **Cognitive**  Delirium  Yes |
| --- | --- | --- | --- | --- | --- | --- | --- | --- | --- | --- | --- | --- | --- | --- | --- | --- | --- | --- | --- | --- | --- | --- | --- | --- | --- | --- | --- | --- | --- | --- | --- | --- | --- | --- | --- | --- | --- | --- | --- |
| Juneau et al. (2018) |  |  | * |  | I  P | * |  |  |  |  |  |  |  |  |  |  |  |  |  |  |  |  | * |  |  |  |  |  |  |  |  |  | * |  |  |  |  |  | **Physical**  ADL  maintained. |
| King et al.  (2016). |  |  | * |  | N |  |  |  |  |  |  |  |  |  |  |  |  | * | * |  |  |  | * |  |  |  |  |  |  |  |  |  | * |  |  |  |  |  | **Physical**  Mobility  Yes |
| Kratz et al. (2015) |  | * |  | N | * |  | * |  |  |  |  | * | * |  |  |  |  |  | * |  |  |  |  |  |  |  |  |  |  | * |  |  |  |  |  |  |  |  | **Cognitive**  Delirium  Yes |

| Liu et al.  (2018) |  | * |  |  | I  P |  |  |  |  |  |  |  |  |  |  |  |  |  |  |  |  |  |  |  |  |  |  |  |  | * |  | * |  |  |  |  |  |  | **Physical**  Mobility  Yes |
| --- | --- | --- | --- | --- | --- | --- | --- | --- | --- | --- | --- | --- | --- | --- | --- | --- | --- | --- | --- | --- | --- | --- | --- | --- | --- | --- | --- | --- | --- | --- | --- | --- | --- | --- | --- | --- | --- | --- | --- |
| MartinezVelila et al. (2016) |  |  | * |  | I  P | * |  |  |  |  |  |  |  |  |  |  |  |  |  |  |  |  |  |  |  |  |  |  |  |  |  |  |  |  |  |  |  | * | **Cognitive**  Delirium  Yes |
| Milisen et al. (2001). |  | * |  | N |  |  | * |  | * | * |  |  |  |  |  |  |  |  |  |  |  |  |  |  |  |  |  |  | * | * |  | * |  |  |  |  |  |  | **Cognitive**  Delirium  Yes |
| Miller et al. (2004) |  | * |  | N |  | * | * |  | * |  |  |  |  |  |  |  |  |  |  | * |  |  |  |  |  |  |  |  | * |  | * |  |  |  |  |  |  |  | **Physical**  ADL  No |
| Mudge et al. (2008) |  | * |  | I  P | * |  | * |  |  |  |  |  |  |  |  | * |  | * |  |  |  |  |  |  |  |  |  |  |  | * |  |  |  |  |  |  |  |  | **Physical**  ADL  Yes  **Cognitive**  Delirium |

|  |  |  |  |  |  |  |  |  |  |  |  |  |  |  |  |  |  |  |  |  |  |  |  |  |  |  |  |  |  |  |  |  |  |  |  |  |  |  | Yes |
| --- | --- | --- | --- | --- | --- | --- | --- | --- | --- | --- | --- | --- | --- | --- | --- | --- | --- | --- | --- | --- | --- | --- | --- | --- | --- | --- | --- | --- | --- | --- | --- | --- | --- | --- | --- | --- | --- | --- | --- |
| Mudge et  al. (2020). |  | * |  | I  P | * |  | * |  |  |  |  | * |  |  |  | * |  | * | * |  |  |  |  |  |  |  |  |  |  |  |  |  |  |  | * |  |  |  | **Physical**  Yes  **Cognitive**  Delirium  Yes |
| Mudge et  al. (2022). | * |  |  | I  P | * |  | * |  |  |  |  | * |  |  |  |  |  | * | * |  |  |  | * | * |  |  |  |  |  |  |  |  |  | * |  |  |  |  | **Physical**  No  (composite score)  **Cognitive**  Delirium  Yes |
| Mudge et al. (2023) | * |  |  | I  P | * |  | * |  |  |  |  | * |  |  |  |  |  | * | * |  |  | * |  | * |  |  |  |  |  |  |  |  |  | * |  |  |  |  | **Physical**  Yes **Cognitive/ social**  Yes |

| Naylor et al. (2014). |  |  | * |  |  |  | N |  |  |  |  |  |  |  |  |  |  |  |  |  |  |  |  |  |  |  |  |  |  | * |  |  |  |  |  |  |  | * | **Physical**  ADL/IADL  No |
| --- | --- | --- | --- | --- | --- | --- | --- | --- | --- | --- | --- | --- | --- | --- | --- | --- | --- | --- | --- | --- | --- | --- | --- | --- | --- | --- | --- | --- | --- | --- | --- | --- | --- | --- | --- | --- | --- | --- | --- |
| Peyrusque  et al.  (2021) |  |  | * |  | I  P | * |  |  |  |  |  |  |  |  |  |  |  |  |  |  |  |  | * |  |  |  |  |  |  |  |  |  |  | * |  |  |  |  | **Physical**  ADL,  Mobility  Yes |
| Rodrigues  et al.  (2020). |  | * |  | N | * |  | * |  |  |  |  | * |  |  |  |  |  | * | * | * |  |  |  |  |  |  |  |  |  |  |  |  |  |  |  |  |  |  | **Physical**  ADL  Yes |
| Rubin et al. (2011) |  | * |  | I  P | * | * | * |  |  |  |  | * | * |  |  |  |  |  |  | * |  |  |  |  |  |  |  |  | * | * |  |  | * | * |  |  |  |  | **Cognitive**  Delirium  Yes |
| Suwanpas  u et al.  (2015) |  | * |  | N | * |  | * | * |  |  | * | * | * | * |  | N |  |  |  |  |  |  |  |  |  |  |  |  | * |  |  |  |  | * |  |  |  |  | **Physical**  ADL No |

| Vidan et al. (2009) |  | * |  | N |  | * | * |  | * |  |  | * |  | * |  |  |  |  | * |  |  |  |  |  | * |  |  |  | * |  |  | * |  |  |  |  |  |  | **Physical**  ADL  Yes  **Cognitive**  Delirium  Yes |
| --- | --- | --- | --- | --- | --- | --- | --- | --- | --- | --- | --- | --- | --- | --- | --- | --- | --- | --- | --- | --- | --- | --- | --- | --- | --- | --- | --- | --- | --- | --- | --- | --- | --- | --- | --- | --- | --- | --- | --- |
| von  RentelnKruse &  Krause  (2007). |  |  | * |  |  | * |  | * |  |  |  |  |  | I  P |  |  |  |  |  |  |  | * |  |  |  |  |  |  |  |  |  |  |  |  |  |  |  |  | **Physical**  Falls risk  Yes |
| Wand et al. (2014) |  | * |  | I  P |  |  | * | * | * |  |  | * | * |  |  |  |  |  | * |  |  |  |  |  | * |  |  |  |  | * |  | * |  |  |  |  |  |  | **Physical**  ADL Yes  **Cognitive**  Delirium  Yes |
| Wang et al. (2020) |  | * |  | I  P | * | * | * |  | * |  |  | * | * |  |  | * |  |  |  |  |  |  |  |  |  |  |  |  |  |  |  |  |  | * |  |  |  |  | **Physical**  ADL  Yes  **Cognitive**  Delirium  Yes |
| Wanich et al. (1992) |  | * |  | N | * | * | * |  |  | * |  |  |  |  |  |  |  |  | * |  |  | * |  |  |  |  |  |  | * |  |  |  |  |  |  |  |  |  | **Physical**  ADL  Yes |
| Zisberg et al. (2018). |  |  | * |  | I  P | * |  |  |  |  |  |  |  |  |  |  |  | * |  |  |  |  | * |  |  |  | * |  |  |  | * |  |  |  |  |  |  |  | **Physical**  Mobility yes |

618 *Note:* N= Nurse; IP=Interprofessional; ADL= activities of daily living; IADL= instrumental activities of daily livin
619 **Supplementary Material**
620 Supplementary Table 1.
621 *Preferred Reporting Items for Systematic reviews and Meta-Analyses extension for Scoping Reviews* 622	*(PRISMA-ScR) Checklist*

| **SECTION** | **ITEM** | **PRISMA-ScR CHECKLIST ITEM** | **REPORTED**  **ON PAGE #** |
| --- | --- | --- | --- |
| **TITLE** | | | |
| Title | 1 | Identify the report as a scoping review. | 1 |
| **ABSTRACT** | | | |
| Provide a structured summary that includes  (as applicable): background, objectives,  Structured eligibility criteria, sources of evidence,  2 4-5 summary charting methods, results, and conclusions  that relate to the review questions and objectives. | | | |
| **INTRODUCTION** | | | |
| Rationale | 3 | Describe the rationale for the review in the context of what is already known. Explain why the review questions/objectives lend themselves to a scoping review approach. | 6-7 |
| Objectives | 4 | Provide an explicit statement of the questions and objectives being addressed with reference to their key elements (e.g., population or participants, concepts, and context) or other relevant key elements used to conceptualize the review questions and/or objectives. | 7 |
| **METHODS** | | | |
| Protocol and registration | 5 | Indicate whether a review protocol exists; state if and where it can be accessed (e.g., a Web address); and if available, provide registration information, including the registration number. | 22 |
| Eligibility criteria | 6 | Specify characteristics of the sources of evidence used as eligibility criteria (e.g., years considered, language, and publication status), and provide a rationale. | 8 |
| Information sources* | 7 | Describe all information sources in the search (e.g., databases with dates of coverage and contact with authors to identify additional sources), as well as the date the most recent search was executed. | 9 |
| Search | 8 | Present the full electronic search strategy for at least 1 database, including any limits used, such that it could be repeated. | 8-9 |

| **SECTION** | **ITEM** | **PRISMA-ScR CHECKLIST ITEM** | **REPORTED**  **ON PAGE #** |
| --- | --- | --- | --- |
| Selection of sources of evidence† | 9 | State the process for selecting sources of evidence (i.e., screening and eligibility) included in the scoping review. | 9 |
| Data charting process‡ | 10 | Describe the methods of charting data from the included sources of evidence (e.g., calibrated forms or forms that have been tested by the team before their use, and whether data charting was done independently or in duplicate) and any processes for obtaining and confirming data from investigators. | 9 |
| Data items | 11 | List and define all variables for which data were sought and any assumptions and simplifications made. | 10 |
| Critical appraisal of individual sources of evidence§ | 12 | If done, provide a rationale for conducting a critical appraisal of included sources of evidence; describe the methods used and how this information was used in any data synthesis (if appropriate). | 10 |
| Synthesis of results | 13 | Describe the methods of handling and summarizing the data that were charted. | 10 |
| **RESULTS** | | | |
| Selection of sources of evidence | 14 | Give numbers of sources of evidence screened, assessed for eligibility, and included in the review, with reasons for exclusions at each stage, ideally using a flow diagram. | 10 |
| Characteristics of sources of evidence | 15 | For each source of evidence, present characteristics for which data were charted and provide the citations. | 10 |
| Critical appraisal within sources of evidence | 16 | If done, present data on critical appraisal of included sources of evidence (see item 12). | 11-12 |
| Results of individual sources of evidence | 17 | For each included source of evidence, present the relevant data that were charted that relate to the review questions and objectives. | 12 |
| Synthesis of results | 18 | Summarize and/or present the charting results as they relate to the review questions and objectives. | 12-18 |
| **DISCUSSION** | | | |
| Summary of evidence | 19 | Summarize the main results (including an overview of concepts, themes, and types of evidence available), link to the review | 18-19 |

| **SECTION** | **ITEM** | **PRISMA-ScR CHECKLIST ITEM** | **REPORTED**  **ON PAGE #** |
| --- | --- | --- | --- |
|  |  | questions and objectives, and consider the relevance to key groups. |  |
| Limitations | 20 | Discuss the limitations of the scoping review process. | 20-21 |
| Conclusions | 21 | Provide a general interpretation of the results with respect to the review questions and objectives, as well as potential implications and/or next steps. | 21 |
| **FUNDING** | | | |
| Funding | 22 | Describe sources of funding for the included sources of evidence, as well as sources of funding for the scoping review. Describe the role of the funders of the scoping review. | 21 |

JBI = Joanna Briggs Institute; PRISMA-ScR = Preferred Reporting Items for Systematic reviews and Meta-Analyses extension for Scoping Reviews.

* Where *sources of evidence* (see second footnote) are compiled from, such as bibliographic databases, social media platforms, and Web sites.

1. A more inclusive/heterogeneous term used to account for the different types of evidence or data sources (e.g., quantitative and/or qualitative research, expert opinion, and policy documents) that may be eligible in a scoping review as opposed to only studies. This is not to be confused with *information sources* (see first footnote).
2. The frameworks by Arksey and O’Malley (6) and Levac and colleagues (7) and the JBI guidance (4, 5) refer to the process of data extraction in a scoping review as data charting*.*
3. The process of systematically examining research evidence to assess its validity, results, and relevance before using it to inform a decision. This term is used for items 12 and 19 instead of "risk of bias" (which is more applicable to systematic reviews of interventions) to include and acknowledge the various sources of evidence that may be used in a scoping review (e.g., quantitative and/or qualitative research, expert opinion, and policy document).

*From:* Tricco AC, Lillie E, Zarin W, O'Brien KK, Colquhoun H, Levac D, et al. PRISMA Extension for Scoping Reviews (PRISMAScR): Checklist and Explanation. Ann Intern Med. 2018;169:467–473[. doi: 10.7326/M18-0850.](http://annals.org/aim/fullarticle/2700389/prisma-extension-scoping-reviews-prisma-scr-checklist-explanation)

641	Supplementary Table 2.

# 642 *Search Strategies for MEDLINE*

643

Line

\#	Search Terms

1 Geriatrics/ or exp Aged/ or Health Services for the Aged/ or (elders or elderly or geriatric* or "old age?" or (seniors not "high school") or "older adult*" or centenarian* or nonagenarian* or octogenarian* or septuagenarian* or sexagenarian* or dottering or decrepit or tottering or overaged or "oldest old").mp.
2 Hospitalization/
3 hospital units/ or hospitals/
4 ((acute adj1 care) or hospital? or "hospital unit?" or hospitali?ation*).mp.
5 2 or 3 or 4
6 1 and 5
7 "Activities of Daily Living"/ 8	(ADL or BADL).ti,kw.
9 ("activit* of daily living" or "daily life activit*").mp.
10 Self Care/
11 ("self care" or selfcare or "personal care").mp.
12 ("functional status" or "functional decline").ti,ab,kw.
13 "functional* independen*".ti,ab,kw.
14 ("functional mobility" or transferring).ti,ab,kw.
15 walking/ or dependent ambulation/
16 ((walk* or mobility or mobili?ation) adj (assisted or unassisted or aided or unaided or independent* or dependent* or abilit* or function*)).ti,ab,kw.
17 (ambulation adj1 (assisted or unassisted or aided or unaided or independent* or dependent* or abilit* or function*)).ti,ab,kw.
18 Hygiene/ or oral hygiene/
19 ("personal hygiene" or "personal grooming" or (hair adj (brush* or comb* or styl*)) or toothbrush* or "tooth brush*" or floss* or "oral care").ti,ab,kw.
20 ((bath* or shower* or clean*) adj (assisted or unassisted or aided or unaided or independent* or dependent*)).ti,ab,kw.
21 (dress* adj1 (assisted or unassisted or aided or unaided or independent* or dependent*)).ti,ab,kw.
22 ((toilet* adj (hygiene or cleanliness or behaviour?r or routine*)) or toileting or continence).ti,ab,kw.
23 (defecation/ or urination/) and (hygiene or hygienic or clean* or behaviour?r or routine*).mp.
24 ("bladder control" or "bowel? control").ti,ab,kw.
25 eating/ or drinking/
26 (((eat* or drink*) adj1 (assisted or unassisted or aided or unaided or independent* or dependent*)) or "self feed*").ti,ab,kw.
27 or/7-26
28 6 and 27
29 (intervention? or program* or initiative? or strateg*).mp.
30 28 and 29
31 case reports.pt.
32 comment.pt.
33 editorial.pt.
34 31 or 32 or 33
35 30 not 34
36 (qualitative* or quantitative* or "mixed-method*").ti,ab,kf.
37 (((systematic or scoping or synthesis or umbrella or integrative or rapid or comprehensive or meta or realist or concept or evidence or narrative or literature) adj3 (review? or anayls?s or synthes?s or ethnography or study or studies or map? or mapping)) or metaanalys?s).ti,kf,pt.
38 meta-analysis/ or "systematic review"/
39 ("health technolog* assessment?" or HTA?).ti,kf,pt.
40 ("randomi?ed control* trial?" or RCT).ti,kf,pt.
41 "clinical trial?".ti,kf,pt.
42 36 or 37 or 38 or 39 or 40 or 41
43 35 and 42

644

645

646	Supplementary Table 3.

# 647 *Inclusion and Exclusion Criteria*

648

Inclusion Criteria

Exclusion Criteria

- Any design (quantitative, qualitative, Fails to satisfy any/all inclusion criteria.

mixed methods) with a focus on initiatives or care delivery models to improve functional outcomes for persons, 65 years of age and older, in hospital

- Address multiple interventions (e.g. maintenance of more than one basic activity of daily living) OR a single focused intervention and outcome (e.g. maintaining mobility or continence).
- Includes educational component for health care providers as part of the practice change
- Report of contextual factors (interventions) that were addressed in addition to education
- Outcome measures that include

(patient) functional outcomes

649	650

Supplementary Fig 1.

*Preferred Reporting Items for Systematic Reviews and Meta-Analyses (PRISMA) Flow Diagram*

Prisma ScR Checklist

[Click here to access/downloa](https://www2.cloud.editorialmanager.com/pone/download.aspx?id=37339735&guid=0a5c2a01-293d-426c-9363-b2af68c1d293&scheme=1)

[d](https://www2.cloud.editorialmanager.com/pone/download.aspx?id=37339735&guid=0a5c2a01-293d-426c-9363-b2af68c1d293&scheme=1)

[Supporting Informatio](https://www2.cloud.editorialmanager.com/pone/download.aspx?id=37339735&guid=0a5c2a01-293d-426c-9363-b2af68c1d293&scheme=1)

[n](https://www2.cloud.editorialmanager.com/pone/download.aspx?id=37339735&guid=0a5c2a01-293d-426c-9363-b2af68c1d293&scheme=1)

[S-1.doc](https://www2.cloud.editorialmanager.com/pone/download.aspx?id=37339735&guid=0a5c2a01-293d-426c-9363-b2af68c1d293&scheme=1)

[x](https://www2.cloud.editorialmanager.com/pone/download.aspx?id=37339735&guid=0a5c2a01-293d-426c-9363-b2af68c1d293&scheme=1)
