# Supplementary material for: Interventions supporting the translation of gerontological evidence into practice to optimize functional outcomes for hospitalized older adults: A scoping review
Source: PLoS One. 2025 Jun 16;20(6):e0324953. doi: 10.1371/journal.pone.0324953 (PMC12169582; doi:10.1371/journal.pone.0324953)
Supplement: Attachment — Submitted filename: PLos_One_Scoping Review_Response to Reviewers_14_01_2025.docx [file pone.0324953.s009.docx]

**Response to Reviewer Comments Received Dec. 30th, 2024**

**Manuscript ID:** PONE-D-24-35120

**Title:** Interventions Supporting the Translation of Gerontological Knowledge into Practice to Improve Functional Outcomes for Hospitalized Older Adults: A Scoping Review

| **Comment** | **Response** | **Changes Made** |
| --- | --- | --- |
| **Journal Requirements** |  |  |
| Please ensure that your manuscript meets PLOS ONE's style requirements, including those for file naming. The PLOS ONE style templates can be found at  <https://journals.plos.org/plosone/s/file?id=wjVg/PLOSOne_formatting_sample_main_body.pdf> and  <https://journals.plos.org/plosone/s/file?id=ba62/PLOSOne_formatting_sample_title_authors_affiliations.pdf> | We have ensured that the manuscript meets PLOS ONE's style requirements, including those for file naming. |  |
| We notice that your supplementary [figures/tables] are included in the manuscript file. Please remove them and upload them with the file type 'Supporting Information'. Please ensure that each Supporting Information file has a legend listed in the manuscript after the references list. | We have relocated the table to the supplementary material as “S-1 Table”. | P. 43. |
| We note you have included a table to which you do not refer in the text of your manuscript. Please ensure that you refer to Table 4 in your text; if accepted, production will need this reference to link the reader to the Table. | We have updated references to the tables throughout the manuscript. |  |
| **Reviewer 1** |  |  |
| The paper chooses to review the papers that contain the element of education for staff. In addition to knowledge deficit, there are numerous factors of why there were insufficient interventions that aimed at physical and cognitive functions. No sufficient reasons and theory support were described to backup the choice of focusing on education. While a typical staff training that aims at improving functional outcomes usually involves staff education, such choice led to authors excluding papers that did not elaborate staff knowledge training. More justifications are needed to support this choice. | We now explain in the introduction why we focused on education. | P. 5. We now indicate that “The implementation of function-oriented approaches, however, has been hampered by inadequate gerontological knowledge on the part of healthcare professionals, which explains our focus on education as a starting point in this review.” |
| More quantitative findings could be cited in the introduction section to show the adverse effects of acute hospital in worsening physical and cognitive function, and thereby highlight the importance of the paper. | We have integrated several recent sources substantiating the adverse effects of acute hospitalization on physical and cognitive function. | P. 3. We now indicate that “Physical activity in acutely hospitalized older adults is highly limited^4, 5^ and loss of ability to perform activities of daily living (ADL) is a common problem. Almost one third of this patient population experience functional decline at discharge.^6^ Crucially, if they do not return to their pre-hospitalization level of function within the 30 days of discharge, they are at high risk for functional decline becoming permanent.^7^” |
| The research priority mentioned on line 94 can be moved to the introduction section. | This line has been moved to the introduction section. | P. 3. |
| More elaborations of the terms in the introduction section are desirable, e.g. “ecological concepts from implementation science” on line 62, “numerous factors on line 80”. | See above. We have clarified that we mean an “ecological lens from implementation science”.  We have also clarified that we mean “a wide array of factors” and have provided examples. | P.4. We now state that “Informed by an ecological lens from implementation science, we conceptualize intervention implementation as influenced by determinants at multiple levels (micro, meso, and macro), which must be addressed conjointly.”  P. 5. We now indicate that “a wide array of micro, meso and macro factors factors (e.g. patient attitudes, organizational culture/climates, financial resources, leadership)^9^ shape healthcare professionals’ perceptions” |
| As per my understanding, acute hospital focuses on short-term medical treatment. For each study, for how long do the participants stay in the hospital and receive the interventions? I wonder if the focus of acute hospital interventions should be “improve” or maintain functional outcomes as stated on line 107 due to the short-term nature of hospital stay. | Length of stay was not consistently reported on across studies, so we are not able to systematically extract this information. We have replaced “improve” with “maintain” and added more explanation. | P. 4. We now indicate that “In acute care settings, healthcare professionals endeavour to maintain older patients’ functioning at their pre-hospitalization baseline, to avoid functional losses becoming permanent.” |
| On line 122-124, “The search focused on physical function and BADL, but both physical and cognitive outcomes were abstracted from any included studies”. Please explain this in the introduction section. | Although our search focused on physical function BADL, both physical and cognitive outcomes were abstracted because evidence indicates that attending to physical function and BADL can also support cognition. Accordingly, if cognitive function was measured and reported on, we included it in our abstraction to lend context to the physical domains that were measured. | P. 7. |
| What are the major physical and cognitive function domains and measures among the papers reviewed? | We now summarize the major physical and cognitive function domains and measures included in the papers reviewed. | P. 12. We now indicate that “The major physical function domains explored were related to mobility, dexterity, and ADL. The major cognitive function domains examined domains including orientation to time and place, recall, etc. The most common measures included the Barthel Index, Katz Activities of Daily Living Scale, Mini Mental State Exam (MMSE), and the Montreal Cognitive Assessment (MoCA).” |
| The intervention categories did not seem to be discussed in detail in discussion section. What is “variety of interventions” as on line 243? Please explain in sufficient detail to show that this is the objective of the paper as stated in the Objective section. | We now discuss the intervention categories in the discussion in greater detail. | P. 13. We now indicate that “ The educational interventions that supported functional outcomes for hospitalized older adults in this review focused on changes to care processes (e.g. multifocal protocols, mobility interventions, and cognitive interventions), environmental changes (e.g. walking trails, clocks, decluttering), administrative support (e.g. purchase of equipment, electronic record modification), facilitation/leadership (e.g. geriatricians and specialized nurses), and discharge/transition planning.” |
| Nurses as primary interventionist vs “Interprofessional approach” is the focus in the first paragraph in discussion section. What are the professions in “Interprofessional approach”? | We now more clearly define “Interprofessional approach” using a WHO definition. | P. 13. We now explain that an interprofessional approach “typically entails multiple health workers from different professional backgrounds working together with patients, families, carers and communities to deliver the highest possible quality care across settings. The professionals involved differ on a case-by-case basis, but in the acute care context, this would typically entail physicians, nurses, and allied health professionals (e.g. physiotherapists, occupational therapists, dieticians etc.). |
| How are the division of roles and cooperation among the professions in the papers reviewed? | We explain in the methods that the division of professional roles in the studies included in our scoping review were analyzed using the same inductive, descriptive approach described in our analysis section in the methods. | P. 9. We now indicate that “we used our descriptive summary to map the educational interventions/practice change initiatives, their purposes, the division of interprofessional roles, as well as the outcomes examined and how they were measured.” |
| The third paragraph in the discussion section concerns sustainability but elaborate if some interventions were expanded to other hospitals. Please define sustainability. | We now include a definition of sustainability. | P. 14. We now indicate that “sustainability” refers to “how the intervention would be maintained after the study (Lennox et al., 2018).” |
| **Reviewer 2** |  |  |
| Title - To simplify/clarify the title: “Interventions Supporting Implementation of Gerontological Education for Healthcare Professionals to Improve Functional Outcomes for Hospitalized Older Adults: A Scoping Review”; could be: interventions supporting gerontological education for healthcare professionals to improve … OR interventions supporting the translation of gerontological knowledge into practice to improve functional outcomes for … OR Interventions Supporting the Implementation of healthcare professionals’ Gerontological knowledge to Improve | We have simplified/clarified the title. | P. 1. The title now reads “Interventions Supporting the Translation of Gerontological Evidence into Practice to Optimize Functional Outcomes for Hospitalized Older Adults: A Scoping Review”. |
| Abstract: |  |  |
| L.30: examine what? Authors may want to add “the characteristics or type of interventions” | This change has been implemented. | P. 2. |
| L40: Interventions included in addition to educational strategies, care processes … | This change has been implemented. | P. 2. |
| L40: environmental changes and leadership roles. | This change has been implemented. | P. 2. |
| Add relevant key words to facilitate retrieval of the study (may include healthcare professionals, implementation science) | Additional keywords “healthcare professionals” and “implementation science” have now been included. | P. 2. |
| Add source of evidence to the abstract (as per Prisma) | We now explain in the abstract that was primary studies were the source of evidence. | P. 2. We now indicate that “Target studies were primary research studies that examined…” |
| Words describing the main examined concept should be unified and maintained throughout the manuscript starting with the title and especially when talking about aims and objectives in different sections to avoid misleading the readers. When I first read the manuscript, I wasn’t sure whether the focus was merely on educational interventions or other supportive strategies to implement knowledge and improve functional outcomes. It is until the results section that things got clear. It could be “initiatives supporting educational interventions for healthcare professionals to improve functional outcomes among hospitalized older adults” OR as per the title “interventions supporting gerontological education for healthcare professionals to improve…” RATHER THAN “interventions that have been used to support the implementation of gerontological knowledge by hospital-based healthcare professionals and/or interprofessional teams to improve older adults’ functioning” (in the abstract) | We have ensured consistent language across the different section of the manuscript. To enhance clarity, we have implemented the same phrasing as the title. | P. 2. We now indicate that “The objective of this study was to examine the characteristics or type of interventions supporting the translation of gerontological knowledge into practice to improve functional outcomes for hospitalized older adults.” |
| - L34: “Target studies examined initiatives or care delivery models to improve functional outcomes”: this could be removed and keep in L35: “Included studies described an educational intervention for healthcare professionals as part of a wider initiative to promote practice change and assessed functional outcomes (physical and/or cognitive)”. | This change has been implemented. | P. 2. We now indicate that “Included studies were primary research studies that described an educational intervention for healthcare professionals as part of an initiative to promote practice change and assessed functional outcomes (physical and/or cognitive).” |
| Introduction: - Justify the use of the ScR approach; why authors did not choose other approaches? As per Prisma checklist and Peters’ et al., the objectives of this review should be aligned with those of the ScR approach: “Explain why the review questions/objectives lend themselves to a scoping review approach”. (for instance: explore the breadth or depth of the literature, map and summarize the evidence, inform future research, and identify or address knowledge gaps, heterogeneous literature, precursor of systematic review, identify key characteristics or factors related to the concept). | A justification for our SCR approach was included in the methods section and has now been moved up to the introduction section to contextualize the objectives. | P. 6. |
| Methods - L92 “We used the steps for scoping reviews recommended by Peters et al.” better to say guidelines as this reference does not include steps. It rather clarifies guidelines related to ScR conduct, in line with PRISMA-ScR. | This change has been implemented. | P. 6. |
| L93 “Our project sought to understand how healthcare professionals’ gerontological knowledge can be improved and applied, one of the research priorities identified in a provincial priority-setting partnershi”: the wording of the ScR aim should be consistent. While it was in the aim section, “to determine what interventions for hospital-based healthcare professionals and/or interprofessional teams, in addition to gerontological education, have been implemented to optimize older adults’ functional outcomes in hospital settings”, ~~here it turned to be “how healthcare professionals’ gerontological knowledge can be improved and applied~~”. Those are two different research questions. | We have ensured that the wording of the ScR aim is consistent throughout the manuscript. |  |
| Line 95-102: justification for the use of ScR approach: I suggest this is removed to the introduction section, to anchor the choice of the approach in the available literature, what do we know about the topic and what could this review add. Align the objective of this study with the objectives of the ScR itself. How could ScR help to meet the study aims? | This paragraph has been relocated to the introduction/objectives section. | P. 6. We now indicate that “As we could not locate any existing review in the hospital setting, we determined that a broader scoping review to map the breadth of the available evidence (as opposed to a more focused systematic review) was most appropriate.^”^ |
| L104: refer to th availability of the checklist as supplementary file. | This change has been implemented. | We now indicate that readers should “(See S-2 Table).” |
| Inclusion in L106 (all designs) contradicts L11 (excluded reviews): add to L106 all designs of primary research | This change has been implemented. | P. 6. We now indicate that we “We included primary studies of any design”. |
| L106: there is a need to explain how studies with different languages were handled (translation? Multilingual authors?) | Non-English sources were translated using Google Translate. | P. 6. We now indicate that “eligible sources that were not published in English were translated using were translated using Google Translate” |
| L112 “less than 65” justify here the cutoff age (used by WHO as explained in later sections) | We selected 65+ as our cutoff because this a common cutoff used in the literature (e.g. in many WHO studies. | P. 6. We now indicate that “adults aged 65+ (in line with the age cutoff used in many World Health Organization reports) in acute care hospital settings. |
| L113: specify (supplementary table 3) | This change has been implemented. | P. 6. |
| L119: search strategy: add here the main examined concept: “educational interventions…”. “intervention” as a concept was mentioned in the Medline strategy; however, the population (PCC mnemonic) was absent (health care professionals-interprofessional teams). The cognitive function was also absent but authors mentioned this as a limitation. | Our search strategy was guided an experienced by a medical librarian. We have added “educational interventions” as a main concept. We will consider the population (PCC mnemonic) in our searches for future scoping reviews. | P. 7. We now indicate that “Natural language terms were derived from three main concepts: 1) educational interventions, 2) older hospitalized patients, and 3) Basic Activities of Daily Living (BADL)…” |
| L115-139: the review is not blinded so pay attention to add authors initials. | Author initials have been added. |  |
| Results - L185: suggest to replace "capture” with “characterize” | This change has been implemented. | P. 10. |
| L188: managerial intervention, leadership or facilitation roles | This change has been implemented for leadership or facilitation roles, but we prefer our phrasing “administration/management” to managerial intervention. | PP. 10, 12. |
| L201: medium scale interventions and not studies | This change has been implemented. | P. 10. |
| L201: add “on” after focused | This change has been implemented. | P. 10. |
| Table 1 include details about the studies and is lengthy; authors may decide to include a summarized table in the text and add this one as supplementary file. | The table is now included as supplementary file “S-2 Table. |  |
| Discussion - Strengths: “We also grounded our study in a robust theoretical conceptualization”: not explained-registration link not accessible | We now explain that we are referring to our ecological conceptualization. We have updated the OSF registration link. | P. 16. We now indicate that  “study in a robust, context-sensitive theoretical conceptualization drawn from ecological implementation science frameworks.” |
| L309: referring to 65+ as a WHO definition of older age is not accurate, even in the cited reference. UN defines older people as aged over 60. WHO use 60+ in some reports and 65+ in others. Better to say 65+ as a cutoff age used in many WHO reports. | We now indicate 65+ as used in many WHO reports. | PP. 6, 16. |
| Supplementary files: - At the end of references add all supplementary files (search strategy, inclusion criteria table, and PRISMA flow diagram in addition to the PRISMA checklist) | This change has been implemented. | PP. 24. |
| Prisma flow diagram: o reverse first arrow to the right. | We believe the arrow directions are accurate. |  |
| If 4482 are removed at the level of title and abstract screening, what are the 3921 removed first? Are they duplicates? | The 4482 papers were removed because they were not relevant, which is now specified in the PRISMA Flow Diagram. | S-1 Figure. We now indicate that “Studies excluded as irrelevant (n = 4482)” |
| The box of excluded studies (screening level) is incomplete. The rest might include other exclusion causes like not describing the education intervention (as mentioned earlier in the text). | We have corrected the box on excluded studies. | S-1 Figure. |
