# Supplementary material for: Interventions supporting the translation of gerontological evidence into practice to optimize functional outcomes for hospitalized older adults: A scoping review
Source: PLoS One. 2025 Jun 16;20(6):e0324953. doi: 10.1371/journal.pone.0324953 (PMC12169582; doi:10.1371/journal.pone.0324953)
Supplement: Attachment — Submitted filename: second review SD 21022025 PONE-D-24-35120_R1.docx [file pone.0324953.s008.docx]

PLOS ONE

In er en ion S ppor ing he Tran la ion of Geron ological E idence in o Prac ice o Op imi e F nc ional O come for Ho pi ali ed Older Ad l : A Scoping Re ie

--Man	crip Draf --

| Man crip N mber: | PONE-D-24-35120R1 |
| --- | --- |
| Ar icle T pe: | Re earch Ar icle |
| F ll Ti le: | In er en ion S ppor ing he Tran la ion of Geron ological E idence in o Prac ice o Op imi e F nc ional O come for Ho pi ali ed Older Ad l : A Scoping Re ie |
| Shor Ti le: | In er en ion S ppor ing he Tran la ion of Geron ological E idence in o Prac ice: A Scoping Re ie |
| Corre ponding A hor: | Ka hleen France H n er, PhD  Uni er i of Alber a  Edmon on, Alber a CANADA |
| Ke ord : | older ad l , ho pi al , ed ca ion, f nc ion, heal hcare profe ional , implemen a ion cience, kno ledge ran la ionn |
| Ab rac : | Backgro nd and Objec i e . Ho pi ali ed older ad l are a ri k for bo h ph ical and cogni i e f nc ional decline. To ppor he implemen a ion of in er en ion op imi ing  heir f nc ion, bo h heal hcare profe ional kno ledge and o her fac or ha ma  impac prac ice change ho ld be addre ed. The objec i e of hi d a o e amine he charac eri ic or pe of in er en ion ppor ing he ran la ion of geron ological e idence in o prac ice o op imi e f nc ional o come for ho pi ali ed older ad l .  Ma erial and Me hod . Thi coping re ie ed he me hod and ep recommended b Pe er e al. Si elec ronic da aba e ere earched from da aba e incep ion o 2024. Incl ded die ere primar re earch die ha de cribed an ed ca ional in er en ion for heal hcare profe ional a par of an ini ia i e o promo e prac ice change and a e ed f nc ional o come (ph ical and/or cogni i e).  Re l . Thir -eigh die ere deemed eligible. N mero in er en ion o ppor implemen a ion of kno ledge o impro e f nc ional o come ere iden ified. In addi ion o ed ca ional ra egie , o her in er en ion incl ded, care proce e , en ironmen , admini ra i e ppor , facili a ion/leader hip, and di charge/ ran i ion planning. S die emplo ed a range of mea re o a e f nc ional o come in ho pi ali ed older ad l .  Di c ion. Older die ended o foc on n r e-dri en in er en ion , and ne er one on in erprofe ional in er en ion . Kno ledge gap ere highligh ed in erm of heor , re earch de ign , in er en ion de crip ion , co n r repre en a ion, polic , en ironmen al change , and leader hip role .  F re e al a ion of in er en ion o enhance older ad l f nc ioning ho ld e amine effec i ene and compare ingle- and m l i-in er en ion program .  S ra egie ba ed on implemen a ion cience heor are needed o en re cce f l p ake of effec i e in er en ion , hile al o addre ing real- orld i e in ho pi al ni , ch a hor affing, re o rce con rain , and o ercro ding. |
| Order of A hor : | Ka hleen France H n er, PhD |
|  | Jeffre I. B ler |
|  | Sho ana Shre ha |
|  | Adrian Wagg |
|  | Nick Millar |
|  | France Carr |
|  | Sherr Dahlke |
|  | C. All on Jone |
| Re pon e o Re ie er : | Hello, I recei ed a req e o confirm ha o r bmi ion con ain all ra da a req ired o replica e he re l of o r d . Plea e no e ha hi i correc and ha da a |

|  | needed o replica e he d i incl ded in he pplemen ar file .  Regard ,  Ka hleen H n er |
| --- | --- |
| Addi ional Informa ion: |  |
| Q e ion | Re pon e |
| Financial Di clo re  En er a financial di clo re a emen ha de cribe he o rce of f nding for he ork incl ded in hi bmi ion. Re ie  he bmi ion g ideline for de ailed  req iremen . Vie p bli hed re earch ar icle from PLOS ONE for pecific e ample .  Thi a emen i req ired for bmi ion  and ill appear in he p bli hed ar icle if he bmi ion i accep ed. Plea e make re i i acc ra e. | Ye |
| F nded die  En er a a emen i h he follo ing de ail : Ini ial of he a hor ho recei ed each a ard  Gran n mber a arded o each a hor  The f ll name of each f nder  URL of each f nder eb i e  Did he pon or or f nder pla an role in he d de ign, da a collec ion and  anal i , deci ion o p bli h, or prepara ion of he man crip ? |  |
| Did o recei e f nding for hi ork? |  |
| Plea e add f nding de ail . a follo - p o "Financial Di clo re  En er a financial di clo re a emen ha de cribe he o rce of f nding for he ork incl ded in hi bmi ion. Re ie  he bmi ion g ideline for de ailed  req iremen . Vie p bli hed re earch ar icle from PLOS ONE for pecific e ample .  Thi a emen i req ired for bmi ion | KFH a a arded a Kno ledge S n he i Gran from he Senior Heal h S ra egic  Clinical Ne ork (SCN), Alber a Heal h Ser ice (no gran # a igned). Alber a Heal h Ser ice did no pla an role in he he d de ign, da a collec ion and anal i , deci ion o p bli h, or prepara ion of he man crip . h p :// .alber aheal h er ice .ca/re earch/Page14021.a p |

| and ill appear in he p bli hed ar icle if he bmi ion i accep ed. Plea e make re i i acc ra e. |  |
| --- | --- |
| F nded die  En er a a emen i h he follo ing de ail : Ini ial of he a hor ho recei ed each a ard  Gran n mber a arded o each a hor  The f ll name of each f nder  URL of each f nder eb i e  Did he pon or or f nder pla an role in he d de ign, da a collec ion and  anal i , deci ion o p bli h, or prepara ion of he man crip ? |  |
| Did o recei e f nding for hi ork?" |  |
| Plea e elec he co n r of o r main re earch f nder (plea e elec caref ll a in ome ca e hi i ed in fee calc la ion). a follo - p o "Financial Di clo re  En er a financial di clo re a emen ha de cribe he o rce of f nding for he ork incl ded in hi bmi ion. Re ie  he bmi ion g ideline for de ailed  req iremen . Vie p bli hed re earch ar icle from PLOS ONE for pecific e ample .  Thi a emen i req ired for bmi ion  and ill appear in he p bli hed ar icle if he bmi ion i accep ed. Plea e make re i i acc ra e. | CANADA - CA |
| F nded die  En er a a emen i h he follo ing de ail : Ini ial of he a hor ho recei ed each a ard  Gran n mber a arded o each a hor  The f ll name of each f nder  URL of each f nder eb i e  Did he pon or or f nder pla an role in he d de ign, da a collec ion and  anal i , deci ion o p bli h, or prepara ion of he man crip ? |  |
|  |  |

| Did o recei e f nding for hi ork?" |  |  |
| --- | --- | --- |
| Compe ing In ere  U e he in r c ion belo o en er a compe ing in ere a emen for hi bmi ion. On behalf of all a hor ,  di clo e an compe ing in ere ha co ld be percei ed o bia hi  ork ackno ledging all financial ppor  and an o her rele an financial or nonfinancial compe ing in ere .  Thi a emen i req ired for bmi ion  and ill appear in he p bli hed ar icle if he bmi ion i accep ed. Plea e make  re i i acc ra e and ha an f nding o rce li ed in o r F nding Informa ion la er in he bmi ion form are al o  declared in o r Financial Di clo re a emen .  Vie p bli hed re earch ar icle from PLOS ONE for pecific e ample . | The a hor ha e declared ha no compe ing in ere | e i . |
| NO a hor ha e compe ing in ere  En er: The a hor hae declared ha no compeing inere ei .  A hor i h compe ing in ere  En er compe ing in ere de ail beginning i h hi a emen :  I hae read he jornal' polic and he a hor of hi man crip hae he follo ing compeing inere : [iner compeing inere here] |  |  |
| * pe e |  |  |
| E hic S a emen  En er an e hic a emen for hi bmi ion. Thi a emen i req ired if he d in ol ed: | N/A. |  |

| H man par icipan  H man pecimen or i e  Ver ebra e animal or cephalopod  Ver ebra e embr o or i e  Field re earch  Wri e "N/A" if he bmi ion doe no req ire an e hic a emen .  General g idance i pro ided belo . Con l he bmi ion g ideline for de ailed in r c ion . Make re ha all informa ion en ered here i incl ded in he Me hod ec ion of he man crip . |  |
| --- | --- |

| Forma for pecific d pe  H man S bjec Re earch (in ol ing h man par icipan and/or i e)  Gi e he name of he in i ional re ie  board or e hic commi ee ha appro ed he d  Incl de he appro al n mber and/or a a emen indica ing appro al of hi re earch  Indica e he form of con en ob ained ( ri en/oral) or he rea on ha con en a  no ob ained (e.g. he da a ere anal ed anon mo l )  Animal Re earch (in ol ing er ebra e animal , embr o or i e )  Pro ide he name of he In i ional Animal Care and U e Commi ee (IACUC) or o her rele an e hic board ha re ie ed he d pro ocol, and indica e he her he appro ed hi re earch or gran ed a formal ai er of e hical appro al  Incl de an appro al n mber if one a ob ained  If he d in ol ed non-hman primae , add addiional deail abo animal elfare and ep aken o ameliora e ffering If ane he ia, e hana ia, or an kind of animal acrifice i par of he d , incl de briefl hich b ance and/or me hod ere applied  Field Re earch  Incl de he follo ing de ail if hi d in ol e he collec ion of plan , animal, or o her ma erial from a na ral e ing:  Field permi n mber  Name of he in i ion or rele an bod ha gran ed permi ion |  |
| --- | --- |
|  |  |
| Da a A ailabili  A hor are req ired o make all da a nderl ing he finding de cribed f ll a ailable, i ho re ric ion, and from he  ime of p blica ion. PLOS allo rare  e cep ion o addre legal and e hical concern . See he PLOS Da a Polic and FAQ for de ailed informa ion. | Ye - all da a are f ll a ailable i ho re ric ion |

| A Da a A ailabili S a emen de cribing here he da a can be fo nd i req ired a bmi ion. Yo r an er o hi q e ion  con i e he Da a A ailabili S a emen  and ill be p bli hed in he ar icle, if accep ed. |  |  |
| --- | --- | --- |
| Impor an : S a ing da a a ailable on req e from he a hor i no fficien . If o r da a are onl a ailable pon req e , elec No for he fir q e ion and e plain o r e cep ional i a ion in he e bo . |  |  |
| Do he a hor confirm ha all da a nderl ing he finding de cribed in heir man crip are f ll a ailable i ho re ric ion? |  |  |
| De cribe here he da a ma be fo nd in f ll en ence . If o are cop ing o r ample e , replace an in ance of XXX i h he appropria e de ail .  If he da a are held or ill be held in a p blic repo i or , incl de URL , acce ion n mber or DOI . If hi informa ion ill onl be a ailable af er accep ance, indica e hi b icking he bo belo . For e ample: All XXX file  are aailable from he XXX daabae (acce ion nmber() XXX, XXX.).  If he da a are all con ained i hin he man crip and/or S ppor ing Informa ion file , en er he follo ing: All relean daa are ihin he man crip and i Spporing  Informaion file.  If nei her of he e applie b o are able o pro ide de ail of acce el e here, i h or i ho limi a ion , plea e do o. For e ample:  Daa canno be hared pblicl beca e of [XXX]. Daa are aailable from he  XXX In i ional Daa Acce / Ehic Commiee (conac ia XXX) for reearcher ho mee he crieria for acce o confidenial daa.  The daa nderling he re l preened in he d are aailable from (inclde he name of he hird par | All rele an da a are i hin he man | crip and i S ppor ing Informa ion file . |

| and conac informaion or URL). Thi e i appropria e if he da a are o ned b a hird par and a hor do no ha e permi ion o hare he da a.  * pe e |  |
| --- | --- |
| Addi ional da a a ailabili informa ion: |  |

Man scrip	Click he e o acce	/do nload;Man	c i ;BADL_Sco ing

Re ie _ R1_23_01_2025_FINAL_Clean.doc

1

2	**Interventions Supporting the Translation of Gerontological Evidence into Practice to**

# 3 Optimize Functional Outcomes for Hospitalized Older Adults: A Scoping Review

4

5 *Kathleen F. Hunter^1^, Jeffrey I. Butler^1^, Shovana Shrestha^1^, C. Allyson Jones^2^, Adrian Wagg^3^,
6 Nick Millar^1^, Frances Carr^3^, Sherry Dahlke^1^

7

8 ^1^Faculty of Nursing, University of Alberta, Edmonton, Alberta, Canada
9 ^2^Department of Physical Therapy, University of Alberta, Edmonton, Alberta, Canada
10 ^3^Division of Geriatric Medicine, Department of Medicine, University of Alberta, Canada

11

12

13

14

15 * Corresponding author
16 Email: kathleen.hunter@ualberta.ca (KFH)

17

18

19

20

21

22

23

24

# Abstract

**Background and Objectives.** Hospitalized older adults are at risk for both physical and cognitive functional decline. To support the implementation of interventions optimizing their function, both healthcare professional knowledge and other factors that may impact practice change should be addressed. The aim of this study was to examine the characteristics and type of interventions supporting the translation of gerontological evidence into practice to optimize functional outcomes for hospitalized older adults.

**Materials and Methods.** This scoping review used the guidelines recommended by Peters et al. Six electronic databases were searched from database inception to 2024. Included studies were primary research studies that 1) described an educational intervention for healthcare professionals as part of an initiative to promote practice change and 2) assessed functional outcomes (physical and/or cognitive).

**Results.** Thirty-eight studies were deemed eligible. Numerous interventions to support implementation of knowledge to improve functional outcomes were identified. In addition to educational strategies, other interventions included, care processes, environment?, administrative support, facilitation/leadership, and discharge/transition planning. Studies employed a range of measures to assess functional outcomes among hospitalized older adults.

**Discussion.** Older studies tended to focus on nurse-driven interventions, and newer ones on interprofessional interventions. Knowledge gaps were highlighted in terms of theory, research designs, intervention descriptions, country representation, policy, environmental changes, and leadership roles.

Future evaluations of interventions to enhance older adults’ functioning should examine effectiveness and compare single- and multi-intervention programs. Strategies based on implementation science theory are needed to ensure successful uptake of effective interventions,

49	while also addressing real-world issues in hospital units, such as short staffing, resource 50	constraints, and overcrowding.

51 *Keywords:* older adults, hospitals, education, function, healthcare professionals,
52 implementation science, knowledge translation

# Introduction

Older adults in hospital are at risk for both physical and cognitive decline.^1-3^ Physical activity in acutely hospitalized older adults is highly limited,^4, 5^ and the loss of ability to perform activities of daily living (ADL) is a common problem. Almost one third of this patient population experience functional decline at discharge.^6^ Crucially, if older adults do not return to their pre-hospitalization level of function within 30 days of discharge, any functional decline is likely to become permanent.^7^

Many older adults have highly complex care needs and entering the hospital system, which prioritizes acute treatment and system efficiency,^8^ can result in functional losses and poor outcomes.^9, 10^ Factors including limited mobility and suboptimal continence care are associated with functional decline in hospital and can contribute to further decline post-discharge.^10, 11^

Consequently, optimizing function for this patient population should be a health system priority. To address this concern, interventions impacting practice change should be addressed along with healthcare professionals knowledge.^12^ Informed by an ecological lens from implementation science,^13^ we conceptualize intervention implementation as influenced by determinants at multiple levels (micro, meso, and macro), which must be addressed conjointly. This scoping review sought to examine? interventions to support implementation of evidence from gerontological education for hospital-based healthcare professionals and/or interprofessional teams to maintain and restore older adults functioning.

In acute care settings, healthcare professionals endeavour to maintain older patients prehospitalization baseline function, to avoid functional losses becoming irreversible. To optimize function and limit preventable hospital-acquired functional loss, there is a need to shift the focus of care for hospitalized older people to the preservation and restoration of function.^14, 15^ For example, evidence suggests that engaging hospitalized older persons in mobility^16^ and basic activities of daily living (e.g., toileting, dressing)^17^ may prevent both physical and cognitive decline. The implementation of function-oriented approaches, however, has been hampered by inadequate gerontological knowledge on the part of healthcare professionals, which explains our focus on education as a starting point in this review.^18-21^

Yet, education alone does not change practice and not all gerontological education initiatives will spur a transformation in older adults care;^22^ a wide variety of factors (e.g., dedicated champions, strong communication, effective teamwork, and appropriate resources) must be recognized as integral to promoting knowledge uptake .^23, 24^ Consequently, we conceptualize healthcare professional education as necessary to catalyze a shift to more functionoriented approaches, but as insufficient in isolation. It is thus essential to develop a broader understanding of both the education that has been employed, as well as other interventions that have been implemented in acute hospital settings to support the application of new knowledge to optimize older adults functioning.

Previous studies have underscored that, in acute care settings, a wide array of micro, meso and macro factors (e.g. patient attitudes, organizational culture/climates, financial resources, leadership)^13^ shape healthcare professionals’ perceptions around the feasibility of implementing new approaches to care.^25^ However, it remains unknown what educational interventions have been employed, to promote function-oriented care for acutely hospitalized older adults.

# Objectives

In line with a research priority identified in a provincial priority-setting partnership,^26^ our aim was to determine what educational interventions for hospital-based healthcare professionals and/or interprofessional teams, have been implemented to optimize older adults functional outcomes in hospital settings. As we could not locate any existing review in the hospital setting, we determined that a broad scoping review to map the breadth of the available evidence was most appropriate.^27, 28^ Accordingly, we mapped the evidence on educational interventions for healthcare professionals that have been implemented as part of an initiative to drive practice change.

# Materials and Methods

We used the guidelines for scoping reviews recommended by Peters et al.^29^ According to Munn et al., (2022), scoping reviews are a type of evidence synthesis that aim to systematically identify and map the breadth of evidence available on a particular topic, field, concept, or issue, often irrespective of source (i.e., primary research, reviews, non-empirical evidence) within or across particular contexts (page 1).^28^ Informed/guided by this approach, we formulated the research question; identified relevant studies; selected studies; charted/extracted data; and collated, summarized, and reported results. The PRISMA-ScR (Preferred Reporting Items for Systematic Reviews and

Meta-Analyses: Extension for Scoping Reviews) guided our reporting^29, 30^ (See S-1 Table).

# Inclusion/Exclusion Criteria

We included primary studies of any design, with no restrictions on language/year of publication. Eligible sources that were not published in English were translated using Google Translate. Studies focused on initiatives or care delivery models to improve functional outcomes for adults aged 65+ (in line with the age cutoff used in many World Health Organization reports)^31^ in acute care hospital settings. Eligible studies 1) described educational interventions for healthcare professionals as a part of an initiative to promote practice change (e.g., care process change, environmental modifications), 2) assessed functional outcomes in older adults (physical or cognitive), and 3) addressed single or multiple interventions. We excluded reviews and editorials, non-hospital studies, studies focused on adults younger than 65 years old, and studies that did not describe an educational intervention component (see S-2 Table).

# Search Strategy

An academic health sciences librarian (MK) developed the search strategy and conducted the search for relevant published studies. Searches were conducted in 2021 and updated in 2023.

Search terms included a combination of natural language vocabulary and controlled terms (subject headings), where available. Natural language terms were derived from three main concepts: 1) educational interventions, 2) older hospitalized patients, and 3) Basic Activities of Daily Living (BADL) interventions, including interventions addressing specific elements such as mobility, ambulation, and personal hygiene. Search terms for BADL also included language for self-care, functional status, and functional decline (see S-3 Table for search strategies for MEDLINE). The search focused on physical function and BADL, but both physical and cognitive outcomes were abstracted from any included studies. Both physical and cognitive outcomes were abstracted because evidence indicates that attending to physical function and BADL can also support cognition. Accordingly, if cognitive function was measured and reported, we included it in our abstraction to lend context to the physical domains that were measured. We conducted the search in six electronic databases: MEDLINE via OVID (1946 -

Present); EMBASE via OVID (1974 - Present); Cumulative Index for Nursing and Allied Health

Literature (CINAHL) via EBSCOhost (1946 - Present); Scopus via Elsevier (1972 - Present); Cochrane Library via Wiley (1992 - Present); TRIP Pro Medical Database (1997 - Present). We additionally conducted hand searches of the reference lists of included studies.

# Study Selection

Identified studies were uploaded to Covidence to support knowledge synthesis. After removal of duplicates, research team member pairs (SS and JIB; KFH and SD) independently screened the abstracts. The full text versions of potentially relevant studies were reviewed as pairs and any discrepancies were resolved through discussion and involvement of a third reviewer, if consensus could not be achieved through discussion. Interrater agreement was not evaluated but regular consensus meetings were held for abstract screening.

# Data Extraction

Research assistants (NM and SS) conducted data extraction, which was verified by the principal investigator (KFH) and research coordinator (JIB). We extracted the following elements: (a) study characteristics: author, year, country; (b) study objectives, design, setting, sample; (c) education component(s), contextual component(s) (e.g., level of intervention, care/team process, facilitation) and functional outcomes (see S-2 Table) using a standardized extraction form.

# Data Analysis

We employed an inductive approach that facilitated identification of unanticipated patterns in the data, as well as an ecological lens that allowed us to differentiate between micro, meso, and macro levels of healthcare practice change.^13, 32^ A descriptive numerical summary of the included studies (overall number of studies included, types of study designs, year of publications, countries where studies were conducted, and study settings) and narrative summary of how review findings address the study aim and research question were developed,^29^ and key findings and gaps in the research evidence were identified. We developed preliminary categories, which were iteratively refined as analysis progressed, to provide a descriptive summary. We did not re-frame the study findings beyond what was reported in the original studies. Given that this was a scoping review which should not address more targeted questions of intervention feasibility or effectiveness,^33^ we used our descriptive summary to map the educational interventions/practice change initiatives, their purposes, the division of interprofessional roles, as well as the outcomes examined and how they were measured.

# Results

Database searches located 8,702 citations, 299 of which were included in full-text screening (see PRISMA Flow Diagram in supplementary material).^30^ Of the 38 Included studies which were conducted between 1993 and 2023; 29 were non-randomized studies with a control;^34-62^ five were randomized controlled trials (RCTs)^63-67^; three were mixed-method studies,^68-70^ and one was a quantitative descriptive study.^71^ Most studies were conducted in the

United States (n=10), Australia (n=6), Canada (n=4) and Israel (n=3), two in Italy, Netherlands, UK, Germany, and Spain, and one in Brazil, Belgium, Portugal, Thailand, and China. The sample size of the included studies ranged from 19 to 12,490 older adults (see S-2 Table for a description of the included studies).

# Geriatric/Gerontological Education Initiatives

More than half the studies (n=23) reported a single educational initiative;^34, 47, 48^ ^38, 63, 61,^ 49, 39, 44, 71, 69, 68, 53, 54, 41, 62, 44, 65, 56, 58, 46, 67, 59, while the remaining studies reported two or more

educational initiatives.35, 36, 37, 51, 41, 51, 55, 43, 64, 52, 66, 45, 57, 60, 70

The educational initiatives were described as training (n=22 studies), education session (n=12), lecture/tutorial (n=5), in-service (n=4), poster (n=4), education module (n=3), as well as workshop, on the job coaching/training, presentation, pocket cards, resources, and material (n=2), and seminar, booklet, videos, handout, quiz, case vignette (n=1) (see Table 1 for frequency of educational strategies reported). Half of the studies targeted a single clinical

outcome (n=19),48, 38, 63, 61, 49, 50, 71, 69, 68, 53, 54, 42, 56, 45, 57, 60, 67, 59, 70 and half (n=19) addressed

multiple clinical outcomes.34-37, 39, 47, 39, 46, 51, 41, 52, 41, 55, 62, 43, 44, 58, 64-66, 58 Most single-outcome education focused on mobility (n=11), followed by delirium (n=7) and cognitive deficits (n=1). Studies focusing on multiple clinical outcomes examined diverse topics such as: dementia, communication with patients, falls, and incontinence. Mobility and delirium were common clinical outcomes in studies targeting multiple outcomes (see S-2 Table). Education targeted

nurses (n=18)47, 48, 38, 63, 49, 51, 40, 53, 68, 55, 62, 43, 52, 66, 46, 45, 59, 71 and various members of the

interprofessional team (n=20).34-36, 61, 39, 50, 52, 71, 69, 54, 41, 44, 64, 65, 56-58, 60, 67

# Interventions to Support Implementation of Gerontological Knowledge

We developed the following categories to characterize the interventions that have been implemented to support the implementation of gerontological knowledge and promote practice change: intervention level , care process changes, changes to the built environment, administration/management?, leadership or facilitation roles, and discharge/transition planning. See Table 2. Of note, 16 studies reported on interprofessional interventions, with 16 relying on nurses to implement the intervention.

**Intervention level.** Interventions were categorized into three levels based on scale, with small-scale defined as a single care unit focused on one or two geriatric concerns, medium scale defined as one or more care units addressing multiple geriatric concerns, and large-scale defined as multiple hospitals addressing multiple geriatric concerns. There were four large-scale interventions, one included four hospitals working with regional health organizations in a formalized collaboration.^39^ The other three involved scale up/spread of two previously tested care models: the hospital elder life program (HELP) to 13 hospitals,^71^ and the EAT, WALK,

ENGAGE program to multiple units in four hospitals.^64, 65^ Sixteen were medium-scale interventions that had multifocal geriatric protocols in one hospital and 12 were small-scale, focused on one intervention on one unit.

Medium-scale interventions focused on a variety of interventions that included combinations directed at cognition, pain, continence, mobility, nutrition/hydration, sleep and pressure ulcers, physical function/falls, family involvement, medication reviews/protocols,

geriatric/frailty screening, and volunteers.34-37, 45-48, 51, 40, 53-55, 62, 43, 44, 66, 58-60, 67 Of these, eleven

involved nurses implementing interventions,34, 47, 45-48, 51, 52, 55, 62, 66, 59 and the rest were interprofessional.

Large-scale studies focused on similar interventions to the medium-scale studies but were

implemented in multiple hospitals.^39, 71, 64, 66^ All involved an interprofessional approach.

**Care Process Changes.** Care process changes ranged from a single focused intervention to models of care that addressed multiple geriatric issues. Small-scale studies focused on

mobility,38, 63, 61, 69, 68, 41, 56, 70 cognition,50, 52 falls,57 or continence and mobility.49, 68 Of these, five

focused on nurses implementing interventions,^38, 63, 49, 68, 42^ while the remainder involved an interprofessional approach.

**Environmental Changes.** Environmental changes included walking trails (Boltz et al.,

2014,47, 68, 43, 44, 64-66, 70 orientation strategies (e.g. clocks),47, 40, 51, 52, 68, 53, 44, 64-66, 45, 59, 60

modifications/decluttering,48, 38, 61, 40, 52, 71, 62, 66, 58 and sensory and mobility aids.65, 57, 59 One study examined the impact of adjusted seating and bed heights.^47^

**Administration/Management? Support?.** Administrative support included provision of

equipment,38, 61, 40, 71, 69, 68, 64, 56, 70 restraint policies,40, 71, 45, 60 electronic record modifications,38, 70 and additional staffing.^49, 64, 65^ Only one study involved regional inter-sectoral collaboration.^34^ **Leadership or Facilitation Roles.** Facilitation/leadership roles in practice change

included advanced practice nurses,36, 49-52, 40, 71, 55, 62, 58, 46, 45, 59 geriatric resource nurse/staff,47, 37, 48, 63, 39, 50, 51, 53-55, 42, 43, 58, 60 and nurse supervisor/case managers.35, 47, 49, 50, 52, 62, 70 Additional

personnel included geriatricians,36, 48, 50-55, 40, 71, 54, 55, 45, 60 interprofessional resource persons,40, 52,

71, 69, 68, 58 and interprofessional geriatric teams.40, 64, 65, 56, 58, 46, 67

**Discharge/Transition Planning.** Discharge/transition planning included

interprofessional family meetings,^36, 37, 44^ links to discharge settings^35, 39, 52^ and minimum data set information.^39, 52^ The most common discharge/transition process involved patient follow-up.^36, 47,^

52, 71, 41, 42

# Measured outcomes

Most studies reported on change in physical function (baseline to discharge).^35, 48, 38, 63, 61,^

49, 51, 69, 68, 54, 62, 52, 56, 66, 46, 57, 59, 70 Only a few studies used self-report38, 66 or caregiver report42 of

pre-hospital function as baseline; the remainder were performance-based assessments by healthcare professionals. Six studies reported on cognitive function, primarily delirium,^37, 50, 53, 41,^ 55, 58 and 12 reported on both physical and cognitive outcomes.34, 36, 47, 40, 52, 71, 43, 44, 65, 45, 60, 67 All studies included nurse or interprofessional multifocal protocols. Two studies reported on a composite score: one an adverse outcome score measuring physical function and high healthcare demand post-hospital,^39^ and the other a composite of hospitalized outcomes such as delirium and pressure injury.^64^ The major physical function domains assessed were related to mobility, dexterity, and ADL. The major cognitive function domains assessed included orientation to time and place, recall etc. The most common measures included the Functional Independence

Measure, Barthel Index, Katz Activities of Daily Living Scale, Mini Mental State Exam

(MMSE), and the Montreal Cognitive Assessment (MoCA).

# Discussion

The educational interventions that supported functional outcomes for hospitalized older adults in this review focused????????? on changes to care processes (e.g. multifocal protocols, mobility interventions, and cognitive interventions), environmental changes (e.g. walking trails, clocks, decluttering), administrative support (e.g. purchase of equipment, electronic record modification), facilitation/leadership? (e.g. geriatricians and specialized nurses), and discharge/transition planning. We used a tripartite conceptualization to describe the scale of the interventions. Half the studies (18/38) concentrated on nurses for education and the delivery of the intervention. This could be because nurses make up almost half of the global health workforce^72^ and are the healthcare professionals that interact the most with patients in hospitals.^73^ In recent years, there has been increasing recognition of the importance of interprofessional collaboration in complex care.^14^ The trend from uni-professional interventions to multi-professional interventions was observed in our findings; studies published in 2013 or

earlier^48, 51, 40, 43, 62, 59^ tended to focus exclusively on nurse-driven interventions, whereas studies

published in the last decade (e.g.,34-36, 61, 39, 54, 44, 64, 56, 37, 67, 71) typically incorporated an interprofessional approach. This typically entails multiple healthcare workers from different professional backgrounds working together with patients, families, carers and communities to deliver the highest possible quality care across settings.^74^ The professionals involved differ on a case-by-case basis, but in the acute care context, this would normally involve physicians, nurses, and allied health professionals (e.g. physiotherapists, occupational therapists, dieticians etc.).

The studies reported that educational interventions to enhance knowledge uptake, had a measurable effect on functional outcomes, but were overwhelmingly physical and/or cognitive, with very few focusing on cognitive function exclusively. Cognitive function is now receiving more scholarly attention^75^ due to increasing recognition that cognitive and physical function are interconnected.^76^ However, our findings echo those of a recent systematic review^3^ which found that cognition is largely ignored; of 94 included studies, 93 focused exclusively on physical deconditioning.

Our review also highlights gaps in the existing research in terms of theory, research designs, intervention descriptions, country representation, policy, and environmental changes. Included studies typically reported on time-limited initiatives and did not address sustainability AND????how the intervention would be maintained after the study^77^. The WALK-FOR program is an

exception as it was developed and tested on two units^38, 70^ and examined the sustainability in a third study.^61^ Another model, EAT WALK ENGAGE, was tested for effectiveness on a single unit and then expanded to eight units in four hospitals,^64, 65^ but was not examined for sustainability. Notably, the HELP model^51^ spread to other hospital sites and was assessed for sustainability.^71^ Moreover, elements of the HELP model were incorporated into other studies.^67^ Future reviews should examine the characteristics of these models/programs that enhance sustainability.

Another gap is the absence of theoretical frameworks; only three studies explicitly used a structured framework to guide development/implementation of their intervention model,^64, 65, 70^ likely due to studies pre-dating recent advances in implementation science. Moreover, many included studies seemed to view change as a linear process, although current theoretical work emphasizes complexity and construes change as non-linear.^78, 33^ As such, some included studies failed to account for contextual elements previously identified as barriers to older adults care (e.g., short staffing, lack of resources, a medical focus, inter-professional team inaccessibility during evenings and weekends).^14,^ ^79-83^ We suggest that future work in this area be guided by implementation theory.

Other gaps identified include a lack of rigorous designs, descriptions of interventions, education components, and representation. Only five of the 38 studies used a randomized controlled design. While we are conscious that randomized controlled trials (RCTs), typically viewed as the gold standard in clinical research, are not always the most appropriate method for every research question and can be undermined by high costs, ethical considerations, and insufficient generalizability to broader, more diverse populations, we believe that more rigorous studies on the topic are needed. Such studies should include RCTs as well as qualitative and mixed methods approaches to understand the complexity of practice change. Descriptions of interventions were inconsistently presented, making it difficult to understand the education intervention and other initiatives provided to support implementation. A large number of studies were excluded during the review process because the education element was not described.

Included studies generally did not concentrate on policy and environmental changes, despite evidence that the built environment can contribute to improved functioning in older adults.^84^ Most of the included environmental changes were minor/superficial (e.g., clocks, decluttering). No studies described a major renovation to the built environment, possibly because the cost is prohibitive. There was little evidence on administrative changes to support

interventions; a few studies describing restraint policies or practices were identified.^40, 71, 45, 60^ Most administrative support was minor except for Heim et al.,^39^ in which a system change was undertaken.

# Strengths and Limitations

This study adds to the literature on care for hospitalized older adults by synthesizing the evidence on education that supported intervention implementation, scaling/spread, and sustainability, and by providing a close examination of other interventions supporting the implementation of gerontological knowledge, which are typically overlooked in reviews. We also grounded our study in a robust, context-sensitive theoretical conceptualization drawn from ecological implementation science frameworks. We demonstrated that prior initiatives are mostly local in scope and piece-meal, with very few initiatives cutting across a healthcare system. We searched the literature in other languages, thereby accessing valuable studies that would have been excluded in a review with language filters?. However, we did not search the grey literature, dissertations/theses, or Clinical Practice Guidelines. Moreover, it is possible that education may have occurred in some studies that was not reported on (and therefore mistakenly excluded when assessing eligibility). We also focused our search on physical function and did not include cognitive interventions or other environmental interventions in our search terms (though we did extract any cognitive outcomes reported). Future reviews should explicitly include cognitive function. Additionally, we adhered to the definition of older adults as aged 65 + used in many World Health Organization reports^31^, admittedly an arbitrary cutoff point, and we thus excluded studies with younger definitions of older adult that may have otherwise been eligible. Finally, as with all scoping reviews, there is an unknown risk of publication bias due to the tendency of researchers to publish only positive findings. Finally, most studies were conducted in highincome countries and may not be generalizable to low- and middle-income countries.

# Directions for Future Research and Policy

We conceive of this scoping review, which mapped the breadth of the existing evidence, as a precursor to a future systematic review.^33^ Future systematic reviews should build on our work by examining which of the interventions that we identified are most effective in optimizing the functioning of hospitalized older adults, and by comparing single interventions with multiintervention programs. Scholars should then develop strategies based on implementation science theory to ensure successful implementation of the most effective interventions while taking into consideration the realities of hospital units, such as short staffing, resource constraints, and overcrowding. Researchers reporting on similar interventions should describe the education component of interventions to enhance transparency and facilitate replication. Further work needs to be undertaken to integrate care of older persons across healthcare systems, and policylevel changes are required to facilitate the provisioning of function-oriented care.

# Funding

This work was supported by a Knowledge Synthesis Grant from the Seniors Health

Strategic Clinical Network	(SCN), Alberta Health Services.

**Conflict of Interest**

We have no conflict of interest to declare.

# Acknowledgements

All data and materials are included in the main manuscript and supplementary materials. This research was pre-registered with the Open Science Framework, registration #: gr7nt; https://osf.io/gr7nt

References

1. Loyd C, Markland AD, Zhang Y, Fowler M, Harper S, Wright NC, Carter CS, Buford

TW, Smith CH, Kennedy R, Brown CJ. Prevalence of hospital-associated disability in

older adults: a meta-analysis. Journal of the American Medical Directors Association. 2020 Apr 1;21(4):455-61

2. Boucher EL, Gan JM, Rothwell PM, Shepperd S, Pendlebury ST. Prevalence and outcomes of frailty in unplanned hospital admissions: a systematic review and metaanalysis of hospital-wide and general (internal) medicine cohorts. EClinicalMedicine. 2023 May 1;59.
3. Chen Y, Almirall Sánchez A, Mockler D, Adrion E, Domínguez Vivero C, Romero Ortu o R. Hospital associated deconditioning: Not only physical, but also cognitive. International Journal of Geriatric Psychiatry [Internet]. 2022 Feb 10;37(3). Available from: https://www.ncbi.nlm.nih.gov/pmc/articles/PMC9303382/
4. Fisher SR, Graham JE, Ottenbacher KJ, Deer R, Ostir GV. Inpatient walking activity to predict readmission in older adults. Archives of physical medicine and rehabilitation. 2016 Sep 1;97(9):S226-31.
5. Sallis R, Roddy Sturm Y, Chijioke E, Litman K, Kanter MH, Huang BZ, Shen E, Nguyen HQ. Stepping toward discharge: Level of ambulation in hospitalized patients. Journal of hospital medicine. 2015 Jun;10(6):384-9.
6. Zisberg A, Shadmi E, Gur Yaish N, Tonkikh O, Sinoff G. Hospital associated functional decline: the role of hospitalization processes beyond individual risk factors. Journal of the American Geriatrics Society. 2015 Jan;63(1):55-62.
7. Boyd CM, Landefeld CS, Counsell SR, Palmer RM, Fortinsky RH, Kresevic D, Burant C, Covinsky KE. Recovery of activities of daily living in older adults after hospitalization for acute medical illness. Journal of the American Geriatrics Society. 2008 Dec;56(12):2171-9.
8. Manning L, Islam MdS. A systematic review to identify the challenges to achieving effective patient flow in public hospitals. The International Journal of Health Planning and Management. 2023 Feb 28;38(3).
9. Montero-Errasquín B, Cruz-Jentoft AJ. Acute sarcopenia. Gerontology. 2023 May 30;69(5):519-25.
10. Zisberg A, Shadmi E, Gur-Yaish N, Tonkikh O, Sinoff G. Hospital-Associated Functional Decline: The Role of Hospitalization Processes Beyond Individual Risk Factors. Journal of the American Geriatrics Society. 2015 Jan;63(1):55 62. 11. Dharmarajan K, Han L, Gahbauer EA, Leo Summers LS, Gill TM. Disability and Recovery After Hospitalization for Medical Illness Among Community Living Older Persons: A Prospective Cohort Study. Journal of the American Geriatrics Society. 2020 Feb 21;68(3):486 95.
12. Damschroder LJ, Aron DC, Keith RE, Kirsh SR, Alexander JA, Lowery JC. Fostering implementation of health services research findings into practice: a consolidated framework for advancing implementation science. Implementation Science. 2009 Aug 7;4(1).
13. Nilsen P, Bernhardsson S. Context matters in implementation science: a scoping review of determinant frameworks that describe contextual determinants for implementation outcomes. BMC Health Services Research. 2019 Mar 25;19(1).
14. Dahlke S, Hunter KF, Negrin K, Reshef Kalogirou M, Fox M, Wagg A. The educational needs of nursing staff when working with hospitalised older people. Journal of Clinical Nursing. 2018 Aug 28;28(1-2):221 34.
15. Swinnerton E, Price A. Recognising, reducing and preventing deconditioning in hospitalised older people. Nursing Older People. 2023 Feb 8;35(2).
16. Baldwin CE, Phillips AC, Edney SM, Lewis LK. Recommendations for older adults physical activity and sedentary behaviour during hospitalisation for an acute medical illness: an international Delphi study. International Journal of Behavioral Nutrition and Physical Activity. 2020 May 25;17(1).
17. Taylor NF, Harding KE, Dennett AM, Febrey S, Warmoth K, Hall AJ, et al. Behaviour change interventions to increase physical activity in hospitalised patients: a systematic review, meta-analysis and meta-regression. Age and Ageing. 2021 Jul 24;51(1). 18. Beltran SJ, Miller VJ. COVID-19 and Older Adults: The Time for GerontologyCurriculum across Social Work Programs is Now! Journal of Gerontological Social Work. 2020 Jul 7;1 4.
19. Dahlke S, Kalogirou MR, Swoboda NL. Registered nurses reflections on their educational preparation to work with older people. International Journal of Older People Nursing [Internet]. 2021 Mar 1;16(2):e12363. Available from: https://www.ncbi.nlm.nih.gov/pmc/articles/PMC7988531/#opn12363-bib-0040
20. Kalánková D, Stolt M, Scott PA, Papastavrou E, Suhonen R. Unmet care needs of older people: A scoping review. Nursing Ethics. 2020 Oct 1;28(2):096973302094811.
21. Tavares J, de Lurdes Almeida M, Duarte SFC, Apóstolo J. Older adult care in nursing education: How have curricula been developed? Nurse Education in Practice. 2021 Jan;50:102947.
22. Rangachari P, Rissing P, Rethemeyer K. Awareness of evidence-based practices alone does not translate to implementation: insights from implementation research. Quality management in health care [Internet]. 2013 [cited 2019 Oct 13];22(2):117 25. Available from: https://www.ncbi.nlm.nih.gov/pubmed/23542366
23. Kavanagh A, O Brien L, Maloney S, Osadnik C. Barriers and facilitators to adopting functional maintenance initiatives for acutely hospitalised older adults. Disability and Rehabilitation. 2019 May 13;42(26):3808 15.
24. Soong C, Shojania KG. Education as a low-value improvement intervention: often necessary but rarely sufficient. BMJ Quality & Safety. 2019 Dec 16;29(5):353 7.
25. Hunter KF, Dahlke S, Negrin K, Kalogirou MR, Fox M, Antonio N, et al. The feasibility of implementing education on older person care to practice on medical units: Nurses perceptions and the influence of practice context. International Journal of Older People Nursing. 2019 Aug 22;14(4).
26. Hanson HM, Cowan K, Wagg A. Identifying what matters most for the health of older adults in Alberta: results from a James Lind Alliance Research Priority Setting Partnership. CMAJ Open. 2021 Apr;9(2):E522 8.
27. Campbell F, Tricco AC, Munn Z, Pollock D, Saran A, Sutton A, et al. Mapping reviews, scoping reviews, and evidence and gap maps (EGMs): the same but different	the Big Picture review family. Systematic Reviews. 2023 Mar 15;12(1).
28. Munn Z, Pollock D, Khalil H, Alexander L, McInerney P, Godfrey CM, et al. What are scoping reviews? Providing a formal definition of scoping reviews as a type of evidence synthesis. JBI Evidence Synthesis. 2022 Mar 4;20(4).
29. Peters MDJ, Marnie C, Tricco AC, Pollock D, Munn Z, Alexander L, et al. Updated Methodological Guidance for the Conduct of Scoping Reviews. JBI Evidence Synthesis.

2020 Sep 22;18(10):2119 26.

30. Tricco AC, Lillie E, Zarin W, O Brien KK, Colquhoun H, Levac D, et al. PRISMA Extension for Scoping Reviews (PRISMA-ScR): Checklist and Explanation. Annals of Internal Medicine. 2018 Sep 4;169(7):467 73.
31. World Health Organization. Physical activity. 2004. https://www.who.int/initiatives/behealthy/physical-activity
32. May CR, Johnson M, Finch T. Implementation, context and complexity. Implementation Science [Internet]. 2016 Oct 19 [cited 2024 Jul 16];11(1). Available from: https://www.ncbi.nlm.nih.gov/pmc/articles/PMC5069794/
33. Pollock et al., 2023. Pollock D, Peters MDJ, Khalil H et al. Recommendations for the extraction, analysis, and presentation of results in scoping reviews. JBI Evid Synth. 2023 Mar 1;21(3):520-32.
34. Allegri N, Rosi A, Del Signore F, Cremascoli R, Cappa S, Tassorelli C, et al. Dementiafriendly intervention for hospitalized older adults with cognitive impairments: results of the Italian Dementia-Friendly Hospital Trial (IDENTITÀ). Aging & Mental Health. 2021 Mar 24;1 9.
35. Apolinario D, Yagamuti STF, Dutra AF, de Sousa Lara EM, de Cassia Pires Coli R, & Weber B. Safe hospital for the elderly program: An observational study on the impact in reducing functional decline. *Cad. Saúde Pública*. 2022;*38*(2), 1 16
36. Bakker FC, Persoon A, Sebastian J.H. Bredie, Jolanda van Haren-Willems, Leferink VJ, Luc Noyez, et al. The CareWell in Hospital program to improve the quality of care for frail elderly inpatients: results of a before after study with focus on surgical patients. 2014 Nov 1;208(5):735 46.
37. Bryant EA, Tulebaev S, Castillo-Angeles M, Moberg E, Senglaub SS, O Mara L, et al. Frailty Identification and Care Pathway: An Interdisciplinary Approach to Care for Older Trauma Patients. Journal of the American College of Surgeons. 2019 Jun;228(6):852859.e1.
38. Cohen Y, Zisberg A, Chayat Y, Gur-Yaish N, Gil E, Levin C, et al. Walking for Better Outcomes and Recovery: The Effect of WALK-FOR in Preventing Hospital-Associated Functional Decline Among Older Adults. Newman A, editor. The Journals of Gerontology: Series A. 2019 Mar 2;74(10):1664 70.
39. Heim N, Rolden H, van Fenema EM, Weverling - Rijnsburger AWE, Tuijl JP, Jue P, et al. The development, implementation and evaluation of a transitional care programme to improve outcomes of frail older patients after hospitalisation. Age and Ageing. 2016 Jun 13;45(5):642 50.
40. Inouye SK, Bogardus ST, Charpentier PA, Leo-Summers L, Acampora D, Holford TR, et al. A Multicomponent Intervention to Prevent Delirium in Hospitalized Older Patients. New England Journal of Medicine [Internet]. 1999 Mar 4 [cited 2020 Jan 7];340(9):669

76. Available from: https://www.nejm.org/doi/full/10.1056/NEJM199903043400901

41. Martínez-Velilla N, Garrués-Irisarri M, Ibañez-Beroiz B, Gil-Cabanas J, Richarte-García A, Idoate-Saralegui F, de Paz PC, Cambra K. An exercise program with patient s involvement and family support can modify the cognitive and affective trajectory of acutely hospitalized older medical patients: a pilot study. Aging Clinical and Experimental Research. 2016 Jun;28:483-90.
42. Naylor MD, Hirschman KB, Hanlon AL, Bowles KH, Bradway C, McCauley KM, et al.

Comparison of evidence-based interventions on outcomes of hospitalized, cognitively impaired older adults. Journal of Comparative Effectiveness Research. 2014 May;3(3):245 57.

43. Mudge AM, Giebel AJ, Cutler AJ. Exercising Body and Mind: An Integrated Approach to Functional Independence in Hospitalized Older People. Journal of the American Geriatrics Society. 2008 Apr;56(4):630 5.
44. Mudge AM, McRae P, Donovan PJ, Reade MC. Multidisciplinary quality improvement programme for older patients admitted to a vascular surgery ward ^*^. Internal Medicine Journal. 2020 Jun;50(6):741 8.
45. VidÃ¡n MT, SÃ¡nchez E, Alonso M, Montero B, Ortiz J, Serra JA. An Intervention Integrated into Daily Clinical Practice Reduces the Incidence of Delirium During Hospitalization in Elderly Patients. Journal of the American Geriatrics Society [Internet].

2009 Nov [cited 2019 Sep 16];57(11):2029 36. Available from: https://onlinelibrary.wiley.com/doi/10.1111/j.1532-5415.2009.02485.x

46. Suwanpasu S, Pongpaew W, Praparpak W. Geriatric resource nurse programme to maintain functional status of elderly patients hospitalised for community-acquired pneumonia. Asian Journal of Gerontology & Geriatrics. 2015 Dec;10(2):10.
47. Boltz M, Resnick B, Chippendale T, Galvin J. Testing a Family-Centered Intervention to Promote Functional and Cognitive Recovery in Hospitalized Older Adults. Journal of the American Geriatrics Society. 2014 Dec; *62*(12):2398 2407
48. Chang E, Hancock K, Hickman L, Glasson J, Davidson P. Outcomes of acutely ill older hospitalized patients following implementation of tailored models of care: A repeated measures (pre- and post-intervention) design. International Journal of Nursing Studies. 2007 Sep;44(7):1079 92.
49. Hamilton L, Lyon PS. A Nursing-Driven Program to Preserve and Restore Functional Ability in Hospitalized Elderly Patients. JONA: The Journal of Nursing Administration. 1995 Apr;25(4):30 7.
50. Holt R, Young J, Heseltine D. Effectiveness of a multi-component intervention to reduce delirium incidence in elderly care wards. Age and Ageing. 2013 Aug 26;42(6):721 7. 51. Inouye SK, Wagner DR, Acampora D, Horwitz RI, Cooney LM, Tinetii ME. A Controlled Trial of a Nursing-Centered Intervention in Hospitalized Elderly Medical Patients: The Yale Geriatric Care Program. Journal of the American Geriatrics Society. 1993 Dec;41(12):1353 60.
52. Inouye SK, Bogardus ST, Baker DI, Leo-Summers L, & Cooney, L. M. The hospital elder life program: A model of care to prevent cognitive and functional decline in older hospitalized patients. Journal of the American Geriatrics Society. 2000;*48*(12):1697 1706.
53. Kratz T, Heinrich M, Schlauß E, Diefenbacher A.Preventing postoperative delirium. Deutsches Ärzteblatt International, 2015;*112*:289 296.
54. Liu B, Moore JE, Almaawiy U, Chan WH, Khan S, Ewusie J, et al. Outcomes of Mobilisation of Vulnerable Elders in Ontario (MOVE ON): a multisite interrupted time series evaluation of an implementation intervention to increase patient mobilisation. Age and Ageing. 2017 Jul 15;47(1):112 9.
55. Milisen K, Foreman MD, Abraham IL, De Geest S, Godderis J, Vandermeulen E, et al. A Nurse-Led Interdisciplinary Intervention Program for Delirium in Elderly Hip-Fracture Patients. Journal of the American Geriatrics Society. 2001 May;49(5):523 32.
56. Peyrusqué E, Buckinx F, Bolduc A, Law C, Kergoat MJ, Aubertin-Leheudre M. Potential Efficacy of Pragmatic Exercise Program (Sprint) During Hospitalization in Older Adults on Health Care and Physical Performance: A Pilot Study. The Journal of nutrition, health & aging. 2020 Oct 12; *25*(1):126 133.
57. Von Renteln-Kruse W, Krause T. Incidence of In-Hospital Falls in Geriatric Patients

Before and After the Introduction of an Interdisciplinary Teamâ€ “ Based Fall-

Prevention Intervention. Journal of the American Geriatrics Society. 2007 Dec;55(12):2068 74.

58. Rubin FH, Neal K, Fenlon K, Hassan S, Inouye SK. Sustainability and Scalability of the Hospital Elder Life Program at a Community Hospital. Journal of the American Geriatrics Society. 2011 Feb;59(2):359 65.
59. Wanich CK, Sullivan-Marx EM, Gottlieb GL, Johnson JC. Functional Status Outcomes of a Nursing Intervention in Hospitalized Elderly. Image: the Journal of Nursing Scholarship. 1992 Sep;24(3):201 8.
60. Wand APF, Thoo W, Sciuriaga H, Ting V, Baker J, Hunt GE. A multifaceted educational intervention to prevent delirium in older inpatients: A before and after study. International Journal of Nursing Studies [Internet]. 2014 Jul [cited 2019 Nov 21];51(7):974 82. Available from: https://www.sciencedirect.com/science/article/abs/pii/S0020748913003556
61. Gil E, Zisberg A, Efrat Shadmi, Nurit Gur-Yaish, Ksenya Shulyaev, Yehudith Chayat, et al. Still WALKing-FOR: 2-year sustainability of the WALK FOR intervention. Age and ageing. 2023 Jun 1;52(6).
62. Miller J, Campbell J, Moore K, Schofield A. Elder Care Supportive Interventions Protocol: Reducing Discomfort in Confused, Hospitalized Older Adults. Journal of Gerontological Nursing. 2004 Aug 1;30(8):10 8.
63. Gazineo D, Godino L, Decaro R, Calogero P, Pinto D, Chiari P, et al. Assisted Walking Program on Walking Ability in In Hospital Geriatric Patients: A Randomized Trial. Journal of the American Geriatrics Society. 2020 Nov 12;69(3):637 43.
64. Mudge AM, McRae P, Banks M, Blackberry I, Barrimore S, Endacott J, et al. Effect of a Ward-Based Program on Hospital-Associated Complications and Length of Stay for Older Inpatients. JAMA Internal Medicine. 2022 Mar 1;182(3):274.
65. Mudge A, McRae P, Young A, Blackberry I, Lee Steere K, Barrimore SE, et al. Implementing a ward-based programme to improve care for older inpatients: process evaluation of the cluster randomised CHERISH trial. BMC Health Services Research. 2023 Jun 21;23(1).
66. Rodrigues C, Mendonça D, Martins MM. Effects of a nursing care program focused on basic self-care in older acute medical in-patients: a randomized controlled trial. Porto Biomedical Journal. 2020 Nov;5(6):e086.
67. Wang YY, Yue JR, Xie DM, Carter P, Li QL, Gartaganis SL, et al. Effect of the Tailored, Family-Involved Hospital Elder Life Program on Postoperative Delirium and Function in Older Adults. JAMA Internal Medicine. 2020 Jan 1;180(1):17.
68. King BJ, Steege LM, Winsor K, VanDenbergh S, Brown CJ. Getting Patients Walking: A Pilot Study of Mobilizing Older Adult Patients via a Nurse-Driven Intervention. Journal of the American Geriatrics Society. 2016 Aug 22;64(10):2088 94.
69. Juneau A, Bolduc A, Nguyen P, Leclerc BS, Rousseau J, Dubé F, et al. Feasibility of Implementing an Exercise Program in a Geriatric Assessment Unit: the SPRINT Program. Canadian Geriatrics Journal. 2018 Sep 14;21(3):284 9.
70. Zisberg A, Agmon M, Gur-Yaish N, Rand D, Hayat Y, Gil E. No one size fits all	the development of a theory-driven intervention to increase in-hospital mobility: the WALK-FOR study. BMC Geriatrics. 2018 Apr 13;18(1).
71. Inouye SK, Baker DI, Fugal P, Bradley EH. Dissemination of the Hospital Elder Life Program: Implementation, Adaptation, and Successes. Journal of the American Geriatrics Society. 2006 Oct;54(10):1492 9.
72. World Health Organization. Nursing and Midwifery; 2022.

https://www.who.int/news-room/fact-sheets/detail/nursing-and-midwifery.

73. Butler R, Monsalve M, Thomas GW, Herman T, Segre AM, Polgreen PM, et al. Estimating Time Physicians and Other Health Care Workers Spend with Patients in an Intensive Care Unit Using a Sensor Network. The American Journal of Medicine [Internet]. 2018 Aug;131(8):972.e9 15. Available from: https://www.ncbi.nlm.nih.gov/pubmed/29649458
74. World Health Organization. (2010) Framework for action on interprofessional education and collaborative practice. Geneva, Switzerland: WHO. https://iris.who.int/bitstream/handle/10665/70185/WHO_HRH_HPN_10.3_eng.pdf?sequ ence=1
75. Yi Chia Wei, Heather C, Wen Yi Huang, Yao Liang Chen, Lin C, Chih Ken Chen, et al. White Matter Integrity Underlies the Physical-Cognitive Correlations in Subjective Cognitive Decline. Frontiers in Aging Neuroscience. 2021 Aug 2;13.
76. Fox MT, Sidani S, Butler JI, Skinner MW, Macdonald M, Durocher E, et al. Optimizing hospital-to-home transitions for older persons in rural communities: a participatory, multimethod study protocol. Implementation Science Communications. 2021 Jul 22;2(1).
77. Lennox, L., Maher, L., & Reed, J. (2018). Navigating the sustainability landscape: A systematic review of sustainability approaches in healthcare. Implementation Science,

13(27), 1-17. DOI: 10.1186/s13012-017-0707-4

78. Lanham HJ, Leykum LK, Taylor BS, McCannon CJ, Lindberg C, Lester RT. How complexity science can inform scale-up and spread in health care: understanding the role of self-organization in variation across local contexts. Social science & medicine. 2013 Sep 1;93:194-202.
79. Dermody G, Kovach CR. Nurses Experience With and Perception of Barriers to Promoting Mobility in Hospitalized Older Adults: A Descriptive Study. Journal of Gerontological Nursing. 2017 May 30;43(11):22 9.
80. Fox MT, Butler JI. Nurses perspectives on how operational leaders influence functionfocused care for hospitalised older people. Journal of Nursing Management. 2016 Sep 16;24(8):1119 29.
81. Hower KI, Vennedey V, Hillen HA, Kuntz L, Stock S, Pfaff H, et al. Implementation of patient-centred care: which organisational determinants matter from decision maker s perspective? Results from a qualitative interview study across various health and social care organisations. BMJ Open [Internet]. 2019 Apr;9(4):e027591. Available from: https://bmjopen.bmj.com/content/9/4/e027591
82. Sepulveda-Pacsi AL, Soderman M, Kertesz L. Nurses perceptions of their knowledge and barriers to ambulating hospitalized patients in acute settings. Applied Nursing Research. 2016 Nov;32:117 21.
83. Yous ML, Ploeg J, Kaasalainen S, Martin LS. Nurses Experiences in Caring for Older Adults with Responsive Behaviors of Dementia in Acute Care. SAGE Open Nursing [Internet]. 2019 Jan;5. Available from: https://journals.sagepub.com/doi/full/10.1177/2377960819834127
84. Parke B, Boltz M, Hunter KF, Chambers T, Wolf-Ostermann K, Adi MN, et al. A Scoping Literature Review of Dementia-Friendly Hospital Design. The Gerontologist. 2016 Nov 9;57(4):gnw128.

S-1 **Table:** *Preferred Reporting Items for Systematic reviews and Meta-Analyses extension for Scoping*

*Reviews (PRISMA-ScR) Checklist*

S-2 **Table:** *Table of Included Studies (N=38)*
S-3 **Table**: *Search Strategies for MEDLINE*
S-4 **Table**: *Inclusion and Exclusion Criteria*
S-5 **Figure:** *Preferred Reporting Items for Systematic Reviews and Meta-Analyses (PRISMA)*

*Flow Diagram*

660 Table 1.
661 *Details on Focus of Educational Strategies Utilized by Studies*

S. N First	HCPs included in	Topics covered.	Education author	education	(e.g., delirium, mobility)	strategies used

(Year)	(e.g., in-service,

workshops, pockets cards)

1. Allegri et Yes: Staff (doctors, Dementia, cognitive Training using al. (2022) nurses, physical impairment, depression teaching modules.

therapists and	bipolar psychotic

healthcare assistants).	disorder, delirium, pain,

communication and interaction with patients

2. Apolinario	Yes (nurses,	Fall, pressure injury,	Education targeted

et al.	physiotherapist, speech bronchoaspiration, delirium,	to train

(2022)	therapist, nutritionist,	mechanical restraint,	professionals from

psychologist, and malnutrition, medication different areas. clinical iatrogenesis, and functional Staff were given pharmacologist). decline. resources such as

booklets, video

lessons, infographics, onsite small group training, and training workshops.

| 3. | Bakker et al. (2014). | Yes (Nurses and Physicians). | Delirium, physical decline, fall, frailty screening, medical review, geriatric assessment, stimulation of cognitive and physical activities. | Educational session, on the job coaching. |
| --- | --- | --- | --- | --- |
| 4. | Boltz et al.  (2014) | Yes (Nurses). | Prevention and management of delirium and functional decline; assessment of mood, cognition, and function; incorporation of function-focused care into care routines; ways to communicate, partnering, and motivating patients and family; and discharge planning. | Education session. |

5. Bryant et Yes (nurses, ancillary Delirium prevention, Educational al. (2019) staff, residents, mobility, medication presentation,

physician assistants management/prognostic pocket card, and attending staff assessment, reducing other mandatory who frequently care complications, social educational for older patients in needs/goal setting and injury modules. trauma unit and in the prevention.

ICU).

6. Chang et al Yes (nurses).	Mobility.	In-service.

(2007)

7. Cohen et al Yes (Nurses, nurse	Mobility.	Theoretical and

(2019)	aides).	practical training.

8. Gazineo et	Yes (only one nurse).	Mobility.	Training.

al. (2021)

9. Gill et al	Yes (unit staff	Mobility.	Theoretical and

(2023)	including nurses, nurse	practical training.

aides and physical

therapists).

10. Hamilton	Yes (nurses).	Mobility.	Staff education.

& Lyon

(1995)

11. Heim et al.	Yes (nurses and	Frailty, ADL functioning.	On the job

(2016).	medical staff).	training.

12. Holt et al.	Yes (ward staff	Delirium.	Interactive lecture,

(2013)	including staff nurses	handout, quiz, and healthcare	poster, reference assistants).	materials and case

vignettes.

13 Inouye et Yes (Nurses). Geriatric care (Delirium, Education, al. (1993b) functional impairment, training, lecture

incontinence and pressure	series.

sores).

14. Inouye et	Yes (Research and	Delirium risk factors:	Training.

al. (1999)	clinical nurses).	cognitive impairment, sleep

deprivation, immobility, visual impairment, hearing impairment and dehydration.

15. Inouye et	Yes (Nurses and	Mobility, vision/hearing,	Formal didactic al. (2000)	Physicians) and	volume repletion, feeding	sessions, small

volunteers.	assistance, sleep.	group training, and

resource materials.

16. Inouye et	Unsure (volunteers).	Delirium.	Regular education

al. (2006)	sessions

17. Juneau et Yes (Nurses, Mobility. Training on al. (2018) physicians and exercise program.

physiotherapist).

18. King et al.	Yes (Nurses and	Mobility.	Training.

(2016).	certified nursing

assistants).

19. Kratz et al.	Yes (Nurses).	Delirium.	Training.

(2015)

20. Liu et al.	Yes (Physicians,	Mobility.	Education

(2018)	nurses).	modules.

21. Martinez-	Yes (ward and	Mobility and functional	Education.

Velila et	research team staff).	independence.

al. (2016)

22. Milisen et	Yes (Nurses).	Delirium, depression, and	Educational

al. (2001).	dementia.	poster, training

23. Miller et	Yes (Nurses and	Confusion, discomfort,	Education

al. (2004)	undergraduate nursing	physical care, assessment of	program.

students).	basic needs, patient safety

and therapeutic communications.

24. Mudge et	Yes (Nurses).	Functional independence	Education, group

al. (2008)	and mobility	in-services

25. Mudge et	Yes (Nurses or allied	Nutrition and hydration,	Training, videos.

al. (2022).	health professional).	mobility, meaningful

cognitive and social engagement and in multidisciplinary teamwork.

26. Mudge et	Yes (allied health	Nutrition and hydration,	Training.

al. (2020).	assistants).	mobility, meaningful

cognitive and social engagement and in multidisciplinary teamwork.

27. Mudge et	Yes (allied health or	Nutrition and hydration,	Training including al. (2023)	nursing assistants).	mobility, meaningful	didactic and

cognitive and social	interactive engagement and in	content.

multidisciplinary teamwork.

28. Naylor et	Yes (nurses and	Cognitive deficit.	Web based

al. (2014).	advanced practice	modules,

nurses).	seminars.

29. Peyrusque	Yes (nurses,	Mobility.	Group training.

et al.	physicians,

(2021)	physiotherapists).

30. Rodrigues	Yes (nurses and	Mobility, nutrition.	Training and

et al.	nursing assistants).	regular education

(2020).	sessions.

31. Rubin et	Unsure (Volunteers).	Nutrition, delirium, overall	Training program.

al. (2011)	care of frail older adults.

32. Suwanpasu Yes (nurses).	Sleep disorder, problem with	Training program.

et al.	eating or feeding,

(2015)	incontinence, confusion,

mobility, falls, and skin breakdown.

33. Vidan et	Yes (nurses).	Delirium.	Educational

al. (2009)	session, poster,

cards with recommendations included in the first page of the treatment book.

34. von	Yes (Nurses,	Mobility.	Presentations,

Renteln-	therapeutic staff, and	training.

Kruse &	physicians).

Krause

(2007).

35. Wand et	Yes (nurses, medical	Delirium.	Ward based in-

al. (2014).	staff).	services,

workshops,

tutorials, education session, posters.

36. Wang et	Yes (Medical	Delirium.	Training.

al. (2020)	postgraduates,

majoring in geriatrics or geriatric nursing were trained as coordinators, who took charge of delivering the intervention plan to nurses, surgical doctors and other team

staff).

37. Wanich et	Yes (Nurses).	Delirium.	In-service

al. (1992)	program.

38. Zisberg et	Yes (Nurses, nurse	Mobility.	Online tutorials,

al. (2018).	aides).	face to face

training.

662

| o et al.  (2022) | Apolinar  i |  | al. (2022) | Allegri et |  |  |  |  | **Study** |
| --- | --- | --- | --- | --- | --- | --- | --- | --- | --- |
|  |  |  |  |  | Large |  | **ion** | **intervent** | **Level of** |
| ***** |  |  | ***** |  | Medium |  |  |  |  |
|  |  |  |  |  | Small |  |  |  |  |
| I  P |  |  | N |  | Multifocal geriatric protocols |  |  | (  Nurse only (N); Interprofessional (IP  )) | **Care process changes** |
|  |  |  |  |  | Mobility interventions |  |  |  |  |
| I  P |  |  |  |  | Family involvement |  |  |  |  |
|  |  |  | N |  | Cognitive interventions |  |  |  |  |
|  |  |  |  |  | Continence interventions |  |  |  |  |
|  |  |  | N |  | Pain interventions |  |  |  |  |
|  |  |  | N |  | Medication review/protocol |  |  |  |  |
|  |  |  |  |  | Pressure ulcer prevention |  |  |  |  |
|  |  |  |  |  | Nutrition/Dehydration |  |  |  |  |
|  |  |  |  |  | Sleep protocol |  |  |  |  |
|  |  |  |  |  | P  hysical Function/Falls |  |  |  |  |
| * |  |  |  |  | Geriatric/Frailty screening |  |  |  |  |
| I  P |  |  |  |  | Geriatric patient rounds |  |  |  |  |
|  |  |  |  |  | Volunteers |  |  |  |  |
|  |  |  |  |  | Walking trails |  |  | **changes** | **Environment** |
|  |  |  |  |  | Orientation (clocks) |  |  |  |  |
|  |  |  |  |  | Modifications/Declutter |  |  |  |  |
|  |  |  |  |  | Adjusting seating/bed height |  |  |  |  |
|  |  |  |  |  | Sensory and mobility aids |  |  |  |  |
|  |  |  |  |  | Equipment purchase |  |  | **Supp**  **o**  **rt** | **Administrative** |
|  |  |  |  |  | Additional staffing |  |  |  |  |
|  |  |  |  |  | Restraint  use/policy |  |  |  |  |
|  |  |  |  |  | Patient/family in shift report |  |  |  |  |
|  |  |  |  |  | Electronic record modifications |  |  |  |  |
|  |  |  |  |  | Regional Intersectoral |  |  |  |  |
|  |  |  |  |  | Advanced practice nurse |  |  |  | **Facilitation/Leadership** |
|  |  |  |  |  | Geriatric resource nurse/staff |  |  |  |  |
| * |  |  |  |  | Nurse supervisor/case manager |  |  |  |  |
|  |  |  |  |  | Geriatrician |  |  |  |  |
|  |  |  |  |  | IP resource person |  |  |  |  |
|  |  |  |  |  | IP geria  tric team |  |  |  |  |
|  |  |  |  |  | IP family meetings |  | **Planning** | **Transition** | **Discharge/** |
| * |  |  |  |  | Links to discharge settings |  |  |  |  |
|  |  |  |  |  | Minimum data set/digital info |  |  |  |  |
|  |  |  |  |  | Patient follow up |  |  |  |  |
| ADL  -    Yes | **Physical:** | No | Maintained    **Cognitive** | **Physical** |  | **nt** | **improveme** | **outcomes** | **Functional** |

Table

2

.

663

*Inte*

*r*

*ventions in*

*A*

*ddition to*

*E*

*ducation*

664

SUPPORTING

GERONTOLOGICAL EDUCATION

| u et al.  (2015) | Suwanpas |  | Rubin et  al. (2011) | | Rodrigues  et al.  (2020). | Peyrusque  et al.  (2021) |  | al. (2014). | Naylor et |
| --- | --- | --- | --- | --- | --- | --- | --- | --- | --- |
|  |  |  |  | |  |  |  |  |  |
| * |  |  | * | | * |  |  |  |  |
|  |  |  |  | |  | * | * |  |  |
| N |  | P | I | | N |  |  |  |  |
| * |  |  | * |  | * | I  P |  |  |  |
|  |  |  | * |  |  | * |  |  |  |
| * |  |  | * |  | * |  | N |  |  |
| * |  |  |  |  |  |  |  |  |  |
|  |  |  |  |  |  |  |  |  |  |
|  |  |  |  |  |  |  |  |  |  |
| * |  |  |  |  |  |  |  |  |  |
| * |  |  | * |  | * |  |  |  |  |
| * |  |  | * |  |  |  |  |  |  |
| * |  |  |  |  |  |  |  |  |  |
|  |  |  |  |  |  |  |  |  |  |
| N |  |  |  |  |  |  |  |  |  |
|  |  |  |  |  |  |  |  |  |  |
|  |  |  |  |  | * |  |  |  |  |
|  |  |  |  |  | * |  |  |  |  |
|  |  |  | * |  | * |  |  |  |  |
|  |  |  |  |  |  |  |  |  |  |
|  |  |  |  |  |  |  |  |  |  |
|  |  |  |  |  |  | * |  |  |  |
|  |  |  |  |  |  |  |  |  |  |
|  |  |  |  |  |  |  |  |  |  |
|  |  |  |  |  |  |  |  |  |  |
|  |  |  |  |  |  |  |  |  |  |
|  |  |  |  |  |  |  |  |  |  |
| * |  |  | * |  |  |  |  |  |  |
|  |  |  | * |  |  |  | * |  |  |
|  |  |  |  |  |  |  |  |  |  |
|  |  |  |  |  |  |  |  |  |  |
|  |  |  | * |  |  |  |  |  |  |
| * |  |  | * |  |  | * |  |  |  |
|  |  |  |  |  |  |  |  |  |  |
|  |  |  |  |  |  |  |  |  |  |
|  |  |  |  |  |  |  |  |  |  |
|  |  |  |  |  |  |  | * |  |  |
| ADL No | **Physical** | Yes | Delirium | **Cognitive** | **Physical**    ADL    Yes | **Physical**    ADL,  Mobility    Yes | No | ADL/IADL | **Physical** |

SUPPORTING

GERONTOLOGICAL EDUCATION

INTERVENTIONS SUPPORTING GERONTOLOGICAL EDUCATION

666

43

ad

S

C c e e acce

/d

g I f

a

S-1 Tab e.d c

ad

S

C c e e acce

/d

g I f

a

S2-Tab e.d c

ad

S

C c e e acce

/d

g I f

a

S-3 Tab e.d c

ad

S

C c e e acce

/d

g I f

a

S-4 Tab e.d c

ad

S

C c he e acce

/d

g I f

a

S-5 F g

e.d c

Re i ed Man	crip i h Track Change

INTERVENTIONS

1

2 **Interventions Supporting the Translation of Gerontological Evidence into Practice to**
3 **Optimize Functional Outcomes for Hospitalized Older Adults: A Scoping Review**

4

5 *Kathleen F. Hunter^1^, Jeffrey I. Butler^1^, Shovana Shrestha^1^, C. Allyson Jones^2^, Adrian Wagg^3^,
6 Nick Millar^1^, Frances Carr^3^, Sherry Dahlke^1^

7

8 ^1^Faculty of Nursing, University of Alberta, Edmonton, Alberta, Canada
9 ^2^Department of Physical Therapy, University of Alberta, Edmonton, Alberta, Canada
10 ^3^Division of Geriatric Medicine, Department of Medicine, University of Alberta, Canada

11

12

13

14

15 * Corresponding author
16 Email: kathleen.hunter@ualberta.ca (KFH)

17

18

19

20

21

22

23

24

# Abstract

**Background and Objectives.** Hospitalized older adults are at risk for both physical and cognitive functional decline. To support the implementation of interventions optimizing their function, both healthcare professional knowledge and other factors that may impact practice change should be addressed. The objective of this study was to examine the characteristics or type of interventions supporting the translation of gerontological evidence into practice to optimize functional outcomes for hospitalized older adults.

**Materials and Methods.** This scoping review used the method and steps recommended by Peters et al. Six electronic databases were searched from database inception to 2024. Included studies were primary research studies that described an educational intervention for healthcare professionals as part of an initiative to promote practice change and assessed functional outcomes (physical and/or cognitive).

**Results.** Thirty-eight studies were deemed eligible. Numerous interventions to support implementation of knowledge to improve functional outcomes were identified. In addition to educational strategies, other interventions included, care processes, environment, administrative support, facilitation/leadership, and discharge/transition planning. Studies employed a range of measures to assess functional outcomes in hospitalized older adults.

**Discussion.** Older studies tended to focus on nurse-driven interventions, and newer ones on interprofessional interventions. Knowledge gaps were highlighted in terms of theory, research designs, intervention descriptions, country representation, policy, environmental changes, and leadership roles.

Future evaluations of interventions to enhance older adults functioning should examine effectiveness and compare single- and multi-intervention programs. Strategies based on implementation science theory are needed to ensure successful uptake of effective interventions,

49	while also addressing real-world issues in hospital units, such as short staffing, resource 50	constraints, and overcrowding.

51 *Keywords:* older adults, hospitals, education, function, healthcare professionals,
52 implementation science, knowledge translation

# Introduction

Older adults in hospital are at risk for both physical and cognitive decline.^1-3^ Physical activity in acutely hospitalized older adults is highly limited,^4, 5^ and loss of ability to perform activities of daily living (ADL) is a common problem. Almost one third of this patient population experience functional decline at discharge.^6^ Crucially, if older adults do not return to their pre-hospitalization level of function within 30 days of discharge, any functional decline is likely to become permanent.^7^

Many older adults have highly complex care needs and entering the hospital system, which prioritizes acute treatment and system efficiency,^8^ can result in functional losses and poor outcomes.^9, 10^ Factors including limited mobility and suboptimal continence care are associated with functional decline in hospital and can contribute to further decline post-discharge.^10, 11^

Consequently, optimizing function for this patient population should be a health system priority. To address this concern, interventions impacting practice change should be addressed along with healthcare professionals knowledge.^12^ Informed by an ecological lens from implementation science,^13^ we conceptualize intervention implementation as influenced by determinants at multiple levels (micro, meso, and macro), which must be addressed conjointly. This scoping review sought to examine interventions to support implementation of evidence from gerontological education for hospital-based healthcare professionals and/or interprofessional teams to maintain and restore older adults functioning.

In acute care settings, healthcare professionals endeavour to maintain older patients prehospitalization baseline function, to avoid functional losses becoming irreversible. To optimize function and limit preventable hospital-acquired functional loss, there is a need to shift the focus of care for hospitalized older people to the preservation and restoration of function.^14, 15^ For example, evidence suggests that engaging hospitalized older persons in mobility^16^ and basic activities of daily living (e.g., toileting, dressing)^17^ may prevent both physical and cognitive decline. The implementation of function-oriented approaches, however, has been hampered by inadequate gerontological knowledge on the part of healthcare professionals, which explains our focus on education as a starting point in this review.^18-21^

Yet, education alone does not change practice and not all gerontological education initiatives will spur a transformation in older adults care;^22^ a wide variety of factors (e.g., dedicated champions, strong communication, effective teamwork, and appropriate resources) must be recognized as integral to promoting knowledge uptake .^23, 24^ Consequently, we conceptualize healthcare professional education as necessary to catalyze a shift to more functionoriented approaches, but as insufficient in isolation. It is thus essential to develop a broader understanding of both the education that has been employed, as well as other interventions that have been implemented in acute hospital settings to support the application of new knowledge to optimize older adults functioning.

Previous studies have underscored that, in acute care settings, a wide array of micro, meso and macro factors (e.g. patient attitudes, organizational culture/climates, financial resources, leadership)^13^ shape healthcare professionals perceptions of the feasibility of implementing new approaches to care.^25^ However, it remains unknown what educational interventions have been employed, to promote function-oriented care for acutely hospitalized older adults.

# Objectives

In line with a research priority identified in a provincial priority-setting partnership,^26^ our aim was to determine what educational interventions for hospital-based healthcare professionals and/or interprofessional teams, have been implemented to optimize older adults functional outcomes in hospital settings. As we could not locate any existing review in the hospital setting, we determined that a broad scoping review to map the breadth of the available evidence was most appropriate.^27, 28^ Accordingly, we mapped the evidence on educational interventions for healthcare professionals that have been implemented as part of an initiative to drive practice change.

# Materials and Methods

We used the guidelines for scoping reviews recommended by Peters et al.^29^ According to Munn et al., (2022), scoping reviews are a type of evidence synthesis that aim to systematically identify and map the breadth of evidence available on a particular topic, field, concept, or issue, often irrespective of source (i.e., primary research, reviews, non-empirical evidence) within or across particular contexts (page 1).^28^ Using this approach, we formulated the research question; identified relevant studies; selected studies; charted/extracted data; and collated, summarized, and reported results. The PRISMA-ScR (Preferred Reporting Items for Systematic Reviews and

Meta-Analyses: Extension for Scoping Reviews) guided our reporting^29, 30^ (See S-1 Table).

# Inclusion/Exclusion Criteria

We included primary studies of any design, with no restrictions on language/year of publication. Eligible sources that were not published in English were translated using Google Translate. Studies focused on initiatives or care delivery models to improve functional outcomes for adults aged 65+ (in line with the age cutoff used in many World Health Organization reports)^31^ in acute care hospital settings. Eligible studies described educational interventions for healthcare professionals as a part of an initiative to promote practice change (e.g., care process change, environmental modifications), assessed functional outcomes in older adults (physical or cognitive), and could address single or multiple interventions. We excluded reviews and editorials, non-hospital studies, studies focused on adults younger than 65 years old, and studies that did not describe an educational intervention component (see S-2 Table).

# Search Strategy

An academic health sciences librarian (MK) developed the search strategy and conducted the search for relevant published studies. Searches were conducted in 2021 and updated in 2023.

Search terms included a combination of natural language vocabulary and controlled terms (subject headings), where available. Natural language terms were derived from three main concepts: 1) educational interventions, 2) older hospitalized patients, and 3) Basic Activities of Daily Living (BADL) interventions, including interventions addressing specific elements such as mobility, ambulation, and personal hygiene. Search terms for BADL also included language for self-care, functional status, and functional decline (see S-3 Table for search strategies for MEDLINE). The search focused on physical function and BADL, but both physical and cognitive outcomes were abstracted from any included studies. Both physical and cognitive outcomes were abstracted because evidence indicates that attending to physical function and BADL can also support cognition. Accordingly, if cognitive function was measured and reported, we included it in our abstraction to lend context to the physical domains that were measured. We conducted the search in six electronic databases: MEDLINE via OVID (1946 -

Present); EMBASE via OVID (1974 - Present); Cumulative Index for Nursing and Allied Health

Literature (CINAHL) via EBSCOhost (1946 - Present); Scopus via Elsevier (1972 - Present); Cochrane Library via Wiley (1992 - Present); TRIP Pro Medical Database (1997 - Present). We additionally conducted hand searches of the reference lists of included studies.

# Study Selection

Identified studies were uploaded to Covidence to support knowledge synthesis. After removal of duplicates, research team member pairs (SS and JIB; KFH and SD) independently screened the abstracts. The full text versions of potentially relevant studies were reviewed as pairs and any discrepancies were resolved through discussion and involvement of a third reviewer, if consensus could not be achieved through discussion. Interrater agreement was not evaluated but regular consensus meetings were held for abstract screening.

# Data Extraction

Research assistants (NM and SS) conducted data extraction, which was verified by the principal investigator (KFH) and research coordinator (JIB). We extracted the following elements: (a) study characteristics: author, year, country; (b) study objectives, design, setting, sample; (c) education component(s), contextual component(s) (e.g., level of intervention, care/team process, facilitation) and functional outcomes (see S-2 Table) using a standardized extraction form.

# Data Analysis

We employed an inductive approach that facilitated identification of unanticipated patterns in the data, as well as an ecological lens that allowed us to differentiate between micro, meso, and macro levels of healthcare practice change.^13, 32^ A descriptive numerical summary of the included studies (overall number of studies included, types of study designs, year of publications, countries where studies were conducted, and study settings) and narrative summary of how review findings address the study aim and research question were developed,^29^ and key findings and gaps in the research evidence were identified. We developed preliminary categories, which were iteratively refined as analysis progressed, to provide a descriptive summary. We did not re-frame the study findings beyond what was reported in the original studies. Given that this was a scoping review which should not address more targeted questions of intervention feasibility or effectiveness,^33^ we used our descriptive summary to map the educational interventions/practice change initiatives, their purposes, the division of interprofessional roles, as well as the outcomes examined and how they were measured.

# Results

Database searches located 8,702 citations, 299 of which were included in full-text screening (see PRISMA Flow Diagram in supplementary material).^30^ Of the 38 Included studies which were conducted between 1993 and 2023; 29 were non-randomized studies with a control;^34-62^ five were randomized controlled trials (RCTs)^63-67^; three were mixed-method studies,^68-70^ and one was a quantitative descriptive study.^71^ Most studies were conducted in the

United States (n=10), Australia (n=6), Canada (n=4) and Israel (n=3), two in Italy, Netherlands, UK, Germany, and Spain, and one in Brazil, Belgium, Portugal, Thailand, and China. The sample size of the included studies ranged from 19 to 12,490 older adults (see S-2 Table for a description of the included studies).

# Geriatric/Gerontological Education Initiatives

More than half the studies (n=23) reported a single educational initiative;^34, 47, 48^ ^38, 63, 61,^ 49, 39, 44, 71, 69, 68, 53, 54, 41, 62, 44, 65, 56, 58, 46, 67, 59, while the remaining studies reported two or more

educational initiatives.35, 36, 37, 51, 41, 51, 55, 43, 64, 52, 66, 45, 57, 60, 70

The educational initiatives were described as training (n=22 studies), education session (n=12), lecture/tutorial (n=5), in-service (n=4), poster (n=4), education module (n=3), as well as workshop, on the job coaching/training, presentation, pocket cards, resources, and material (n=2), and seminar, booklet, videos, handout, quiz, case vignette (n=1) (see Table 1 for frequency of educational strategies reported). Half of the studies targeted a single clinical

outcome (n=19),48, 38, 63, 61, 49, 50, 71, 69, 68, 53, 54, 42, 56, 45, 57, 60, 67, 59, 70 and half (n=19) addressed

multiple clinical outcomes.34-37, 39, 47, 39, 46, 51, 41, 52, 41, 55, 62, 43, 44, 58, 64-66, 58 Most single-outcome education focused on mobility (n=11), followed by delirium (n=7) and cognitive deficits (n=1). Studies focusing on multiple clinical outcomes examined diverse topics such as: dementia, communication with patients, falls, and incontinence. Mobility and delirium were common clinical outcomes in studies targeting multiple outcomes (see S-2 Table). Education targeted

nurses (n=18)47, 48, 38, 63, 49, 51, 40, 53, 68, 55, 62, 43, 52, 66, 46, 45, 59, 71 and various members of the

interprofessional team (n=20).34-36, 61, 39, 50, 52, 71, 69, 54, 41, 44, 64, 65, 56-58, 60, 67

# Interventions to Support Implementation of Gerontological Knowledge

We developed the following categories to characterize the interventions that have been implemented to support the implementation of gerontological knowledge and promote practice change: level of intervention, care process changes, environmental changes, administration/management, leadership or facilitation roles, and discharge/transition planning. See Table 2. Of note, 16 studies reported on interprofessional interventions, with 16 relying on nurses to implement the intervention.

**Level of Intervention.** Interventions were categorized into three levels based on scale, with small-scale defined as a single care unit focused on one or two geriatric concerns, medium scale defined as one or more care units addressing multiple geriatric concerns, and large-scale defined as multiple hospitals addressing multiple geriatric concerns. There were four large-scale interventions, one included four hospitals working with regional health organizations in a formalized collaboration.^39^ The other three involved scale up/spread of two previously tested care models: the hospital elder life program (HELP) to 13 hospitals,^71^ and the EAT, WALK,

ENGAGE program to multiple units in four hospitals.^64, 65^ Sixteen were medium-scale interventions that had multifocal geriatric protocols in one hospital and 12 were small-scale, focused on one intervention on one unit.

Medium-scale interventions focused on a variety of interventions that included combinations directed at cognition, pain, continence, mobility, nutrition/hydration, sleep and pressure ulcers, physical function/falls, family involvement, medication reviews/protocols,

geriatric/frailty screening, and volunteers.34-37, 45-48, 51, 40, 53-55, 62, 43, 44, 66, 58-60, 67 Of these, eleven

involved nurses implementing interventions,34, 47, 45-48, 51, 52, 55, 62, 66, 59 and the rest were interprofessional.

Large-scale studies focused on similar interventions to the medium-scale studies but were

implemented in multiple hospitals.^39, 71, 64, 66^ All involved an interprofessional approach.

**Care Process Changes.** Care process changes ranged from a single focused intervention to models of care that addressed multiple geriatric issues. Small-scale studies focused on

mobility,38, 63, 61, 69, 68, 41, 56, 70 cognition,50, 52 falls,57 or continence and mobility.49, 68 Of these, five

focused on nurses implementing interventions,^38, 63, 49, 68, 42^ while the remainder involved an interprofessional approach.

**Environmental Changes.** Environmental changes included walking trails (Boltz et al.,

2014,47, 68, 43, 44, 64-66, 70 orientation strategies (e.g. clocks),47, 40, 51, 52, 68, 53, 44, 64-66, 45, 59, 60

modifications/decluttering,48, 38, 61, 40, 52, 71, 62, 66, 58 and sensory and mobility aids.65, 57, 59 One study examined the impact of adjusted seating and bed heights.^47^

**Administration/Management.** Administrative support included provision of

equipment,38, 61, 40, 71, 69, 68, 64, 56, 70 restraint policies,40, 71, 45, 60 electronic record modifications,38, 70 and additional staffing.^49, 64, 65^ Only one study involved regional inter-sectoral collaboration.^34^ **Leadership or Facilitation Roles.** Facilitation/leadership roles in practice change

included advanced practice nurses,36, 49-52, 40, 71, 55, 62, 58, 46, 45, 59 geriatric resource nurse/staff,47, 37, 48, 63, 39, 50, 51, 53-55, 42, 43, 58, 60 and nurse supervisor/case managers.35, 47, 49, 50, 52, 62, 70 Additional

personnel included geriatricians,36, 48, 50-55, 40, 71, 54, 55, 45, 60 interprofessional resource persons,40, 52,

71, 69, 68, 58 and interprofessional geriatric teams.40, 64, 65, 56, 58, 46, 67

**Discharge/Transition Planning.** Discharge/transition planning included

interprofessional family meetings,^36, 37, 44^ links to discharge settings^35, 39, 52^ and minimum data set information.^39, 52^ The most common discharge/transition process involved patient follow-up.^36, 47,^

52, 71, 41, 42

# Outcomes Measured

Most studies reported on change in physical function (baseline to discharge).^35, 48, 38, 63, 61,^

49, 51, 69, 68, 54, 62, 52, 56, 66, 46, 57, 59, 70 Only a few studies used self-report38, 66 or caregiver report42 of

pre-hospital function as baseline; the remainder were performance-based assessments by healthcare professionals. Six studies reported on cognitive function, primarily delirium,^37, 50, 53, 41,^ 55, 58 and 12 reported on both physical and cognitive outcomes.34, 36, 47, 40, 52, 71, 43, 44, 65, 45, 60, 67 All studies included nurse or interprofessional multifocal protocols. Two studies reported on a composite score: one an adverse outcome score measuring physical function and high healthcare demand post-hospital,^39^ and the other a composite of hospitalized outcomes such as delirium and pressure injury.^64^ The major physical function domains assessed were related to mobility, dexterity, and ADL. The major cognitive function domains assessed included orientation to time and place, recall etc. The most common measures included the Functional Independence

Measure, Barthel Index, Katz Activities of Daily Living Scale, Mini Mental State Exam

(MMSE), and the Montreal Cognitive Assessment (MoCA).

# Discussion

The educational interventions that supported functional outcomes for hospitalized older adults in this review focused on changes to care processes (e.g. multifocal protocols, mobility interventions, and cognitive interventions), environmental changes (e.g. walking trails, clocks, decluttering), administrative support (e.g. purchase of equipment, electronic record modification), facilitation/leadership (e.g. geriatricians and specialized nurses), and discharge/transition planning. We used a tripartite conceptualization to describe the scale of the interventions. Half the studies (18/38) concentrated on nurses for education and the delivery of the intervention. This could be because nurses make up almost half of the global health workforce^72^ and are the healthcare professionals that interact the most with patients in hospitals.^73^ In recent years, there has been increasing recognition of the importance of interprofessional collaboration in complex care.^14^ The trend from uni-professional interventions to multi-professional interventions was observed in our findings; studies published in 2013 or

earlier^48, 51, 40, 43, 62, 59^ tended to focus exclusively on nurse-driven interventions, whereas studies

published in the last decade (e.g.,34-36, 61, 39, 54, 44, 64, 56, 37, 67, 71) typically incorporated an interprofessional approach. This typically entails multiple healthcare workers from different professional backgrounds working together with patients, families, carers and communities to deliver the highest possible quality care across settings.^74^ The professionals involved differ on a case-by-case basis, but in the acute care context, this would normally involve physicians, nurses, and allied health professionals (e.g. physiotherapists, occupational therapists, dieticians etc.).

The studies reported that educational interventions to enhance knowledge uptake, had a measurable effect on functional outcomes, but were overwhelmingly physical and/or cognitive, with very few focusing on cognitive function exclusively. Cognitive function is now receiving more scholarly attention^75^ due to increasing recognition that cognitive and physical function are interconnected.^76^ However, our findings echo those of a recent systematic review^3^ which found that cognition is largely ignored; of 94 included studies, 93 focused exclusively on physical deconditioning.

Our review also highlights gaps in the existing research in terms of theory, research designs, intervention descriptions, country representation, policy, and environmental changes. Included studies typically reported on time-limited initiatives and did not address sustainability how the intervention would be maintained after the study^77^. The WALK-FOR program is an

exception as it was developed and tested on two units^38, 70^ and examined the sustainability in a third study.^61^ Another model, EAT WALK ENGAGE, was tested for effectiveness on a single unit and then expanded to eight units in four hospitals,^64, 65^ but was not examined for sustainability. Notably, the HELP model^51^ spread to other hospital sites and was assessed for sustainability.^71^ Moreover, elements of the HELP model were incorporated into other studies.^67^ Future reviews should examine the characteristics of these models/programs that enhance sustainability.

Another gap is the absence of theoretical frameworks only three studies explicitly used

a structured framework to guide development/implementation of their intervention model,^64, 65, 70^ likely due to studies pre-dating recent advances in implementation science. Moreover, many included studies seemed to view change as a linear process, although current theoretical work emphasizes complexity and construes change as non-linear.^78, 33^ As such, some included studies failed to account for contextual elements previously identified as barriers to older adults care (e.g., short staffing, lack of resources, a medical focus, inter-professional team inaccessibility during evenings and weekends).^14,^ ^79-83^ We suggest that future work in this area be guided by implementation theory.

Other gaps identified include a lack of rigorous designs, descriptions of interventions, education components, and representation. Only five of the 38 studies used a randomized controlled design. While we are conscious that randomized controlled trials (RCTs) typically viewed as the gold standard in clinical research are not always the most appropriate method for every research question and can be undermined by high costs, ethical considerations, and insufficient generalizability to broader, more diverse populations, we believe that more rigorous studies on the topic are needed. Such studies should include RCTs as well as qualitative and mixed methods approaches to understand the complexity of practice change. Descriptions of interventions were inconsistently presented, making it difficult to understand the education and other interventions provided to support implementation. A large number of studies were excluded during the review process because the education element was not described.

Included studies generally did not concentrate on policy and environmental changes, despite evidence that the built environment can contribute to improved functioning in older adults.^84^ Most of the included environmental changes were minor/superficial (e.g., clocks, decluttering). No studies described a major renovation to the built environment, possibly because the cost is prohibitive. There was little evidence of administrative changes to support

interventions; a few studies describing restraint policies or practices.^40, 71, 45, 60^ Most administrative support was minor except for Heim et al.,^39^ in which a system change was undertaken.

# Strengths and Limitations

This study adds to the literature on care for hospitalized older adults by synthesizing the evidence on education that supported intervention implementation, scaling/spread, and sustainability, and by providing a close examination of other interventions supporting the implementation of gerontological knowledge, which are typically overlooked in reviews. We also grounded our study in a robust, context-sensitive theoretical conceptualization drawn from ecological implementation science frameworks. We demonstrated that prior initiatives are mostly local in scope and piece-meal, with very few initiatives cutting across a healthcare system. We searched the literature in other languages, thereby accessing valuable studies that would have been excluded in an English-only review. However, we did not search the grey literature, dissertations/theses, or Clinical Practice Guidelines. Moreover, it is possible that education may have occurred in some studies that was not reported on (and therefore mistakenly excluded when assessing eligibility). We also focused our search on physical function and did not include details of cognitive interventions or other environmental interventions in our search terms (though we did extract any cognitive outcomes reported). Future reviews should explicitly include cognitive function. Additionally, we adhered to the definition of older adults as aged 65 + used in many World Health Organization reports^31^, admittedly an arbitrary cutoff point, and we thus excluded studies with younger definitions of older adult that may have otherwise been eligible. Finally, as with all scoping reviews, there is an unknown risk of publication bias due to the tendency of researchers to publish only positive findings. Finally, most studies were conducted in highincome countries and may not be generalizable to low- and middle-income countries.

# Directions for Future Research and Policy

We conceive of this scoping review, which mapped the breadth of the existing evidence, as a precursor to a future systematic review.^33^ Future systematic reviews should build on our work by examining which of the interventions we identified are most effective in optimizing the functioning of hospitalized older adults, and by comparing single interventions with multiintervention programs. Scholars should then develop strategies based on implementation science theory to ensure successful implementation of the most effective interventions while taking into consideration the realities of hospital units, such as short staffing, resource constraints, and overcrowding. Researchers reporting on similar interventions should describe the education component of interventions to enhance transparency and facilitate replication. Further work needs to be undertaken to integrate care of older persons across healthcare systems, and policylevel changes are required to facilitate the provisioning of function-oriented care.

# Funding

This work was supported by a Knowledge Synthesis Grant from the Seniors Health

Strategic Clinical Network	(SCN), Alberta Health Services.

**Conflict of Interest**

We have no conflict of interest to declare.

# Acknowledgements

All data and materials are included in the main manuscript and supplementary materials. This research was pre-registered with the Open Science Framework, registration #: gr7nt; https://osf.io/gr7nt

References

1. Loyd C, Markland AD, Zhang Y, Fowler M, Harper S, Wright NC, Carter CS, Buford

TW, Smith CH, Kennedy R, Brown CJ. Prevalence of hospital-associated disability in

older adults: a meta-analysis. Journal of the American Medical Directors Association. 2020 Apr 1;21(4):455-61

2. Boucher EL, Gan JM, Rothwell PM, Shepperd S, Pendlebury ST. Prevalence and outcomes of frailty in unplanned hospital admissions: a systematic review and metaanalysis of hospital-wide and general (internal) medicine cohorts. EClinicalMedicine. 2023 May 1;59.
3. Chen Y, Almirall Sánchez A, Mockler D, Adrion E, Domínguez Vivero C, Romero Ortu o R. Hospital associated deconditioning: Not only physical, but also cognitive. International Journal of Geriatric Psychiatry [Internet]. 2022 Feb 10;37(3). Available from: https://www.ncbi.nlm.nih.gov/pmc/articles/PMC9303382/
4. Fisher SR, Graham JE, Ottenbacher KJ, Deer R, Ostir GV. Inpatient walking activity to predict readmission in older adults. Archives of physical medicine and rehabilitation. 2016 Sep 1;97(9):S226-31.
5. Sallis R, Roddy Sturm Y, Chijioke E, Litman K, Kanter MH, Huang BZ, Shen E, Nguyen HQ. Stepping toward discharge: Level of ambulation in hospitalized patients. Journal of hospital medicine. 2015 Jun;10(6):384-9.
6. Zisberg A, Shadmi E, Gur Yaish N, Tonkikh O, Sinoff G. Hospital associated functional decline: the role of hospitalization processes beyond individual risk factors. Journal of the American Geriatrics Society. 2015 Jan;63(1):55-62.
7. Boyd CM, Landefeld CS, Counsell SR, Palmer RM, Fortinsky RH, Kresevic D, Burant C, Covinsky KE. Recovery of activities of daily living in older adults after hospitalization for acute medical illness. Journal of the American Geriatrics Society. 2008 Dec;56(12):2171-9.
8. Manning L, Islam MdS. A systematic review to identify the challenges to achieving effective patient flow in public hospitals. The International Journal of Health Planning and Management. 2023 Feb 28;38(3).
9. Montero-Errasquín B, Cruz-Jentoft AJ. Acute sarcopenia. Gerontology. 2023 May 30;69(5):519-25.
10. Zisberg A, Shadmi E, Gur-Yaish N, Tonkikh O, Sinoff G. Hospital-Associated Functional Decline: The Role of Hospitalization Processes Beyond Individual Risk Factors. Journal of the American Geriatrics Society. 2015 Jan;63(1):55 62. 11. Dharmarajan K, Han L, Gahbauer EA, Leo Summers LS, Gill TM. Disability and Recovery After Hospitalization for Medical Illness Among Community Living Older Persons: A Prospective Cohort Study. Journal of the American Geriatrics Society. 2020 Feb 21;68(3):486 95.
12. Damschroder LJ, Aron DC, Keith RE, Kirsh SR, Alexander JA, Lowery JC. Fostering implementation of health services research findings into practice: a consolidated framework for advancing implementation science. Implementation Science. 2009 Aug 7;4(1).
13. Nilsen P, Bernhardsson S. Context matters in implementation science: a scoping review of determinant frameworks that describe contextual determinants for implementation outcomes. BMC Health Services Research. 2019 Mar 25;19(1).
14. Dahlke S, Hunter KF, Negrin K, Reshef Kalogirou M, Fox M, Wagg A. The educational needs of nursing staff when working with hospitalised older people. Journal of Clinical Nursing. 2018 Aug 28;28(1-2):221 34.
15. Swinnerton E, Price A. Recognising, reducing and preventing deconditioning in hospitalised older people. Nursing Older People. 2023 Feb 8;35(2).
16. Baldwin CE, Phillips AC, Edney SM, Lewis LK. Recommendations for older adults physical activity and sedentary behaviour during hospitalisation for an acute medical illness: an international Delphi study. International Journal of Behavioral Nutrition and Physical Activity. 2020 May 25;17(1).
17. Taylor NF, Harding KE, Dennett AM, Febrey S, Warmoth K, Hall AJ, et al. Behaviour change interventions to increase physical activity in hospitalised patients: a systematic review, meta-analysis and meta-regression. Age and Ageing. 2021 Jul 24;51(1). 18. Beltran SJ, Miller VJ. COVID-19 and Older Adults: The Time for GerontologyCurriculum across Social Work Programs is Now! Journal of Gerontological Social Work. 2020 Jul 7;1 4.
19. Dahlke S, Kalogirou MR, Swoboda NL. Registered nurses reflections on their educational preparation to work with older people. International Journal of Older People Nursing [Internet]. 2021 Mar 1;16(2):e12363. Available from: https://www.ncbi.nlm.nih.gov/pmc/articles/PMC7988531/#opn12363-bib-0040
20. Kalánková D, Stolt M, Scott PA, Papastavrou E, Suhonen R. Unmet care needs of older people: A scoping review. Nursing Ethics. 2020 Oct 1;28(2):096973302094811.
21. Tavares J, de Lurdes Almeida M, Duarte SFC, Apóstolo J. Older adult care in nursing education: How have curricula been developed? Nurse Education in Practice. 2021 Jan;50:102947.
22. Rangachari P, Rissing P, Rethemeyer K. Awareness of evidence-based practices alone does not translate to implementation: insights from implementation research. Quality management in health care [Internet]. 2013 [cited 2019 Oct 13];22(2):117 25. Available from: https://www.ncbi.nlm.nih.gov/pubmed/23542366
23. Kavanagh A, O Brien L, Maloney S, Osadnik C. Barriers and facilitators to adopting functional maintenance initiatives for acutely hospitalised older adults. Disability and Rehabilitation. 2019 May 13;42(26):3808 15.
24. Soong C, Shojania KG. Education as a low-value improvement intervention: often necessary but rarely sufficient. BMJ Quality & Safety. 2019 Dec 16;29(5):353 7.
25. Hunter KF, Dahlke S, Negrin K, Kalogirou MR, Fox M, Antonio N, et al. The feasibility of implementing education on older person care to practice on medical units: Nurses perceptions and the influence of practice context. International Journal of Older People Nursing. 2019 Aug 22;14(4).
26. Hanson HM, Cowan K, Wagg A. Identifying what matters most for the health of older adults in Alberta: results from a James Lind Alliance Research Priority Setting Partnership. CMAJ Open. 2021 Apr;9(2):E522 8.
27. Campbell F, Tricco AC, Munn Z, Pollock D, Saran A, Sutton A, et al. Mapping reviews, scoping reviews, and evidence and gap maps (EGMs): the same but different	the Big Picture review family. Systematic Reviews. 2023 Mar 15;12(1).
28. Munn Z, Pollock D, Khalil H, Alexander L, McInerney P, Godfrey CM, et al. What are scoping reviews? Providing a formal definition of scoping reviews as a type of evidence synthesis. JBI Evidence Synthesis. 2022 Mar 4;20(4).
29. Peters MDJ, Marnie C, Tricco AC, Pollock D, Munn Z, Alexander L, et al. Updated Methodological Guidance for the Conduct of Scoping Reviews. JBI Evidence Synthesis.

2020 Sep 22;18(10):2119 26.

30. Tricco AC, Lillie E, Zarin W, O Brien KK, Colquhoun H, Levac D, et al. PRISMA Extension for Scoping Reviews (PRISMA-ScR): Checklist and Explanation. Annals of Internal Medicine. 2018 Sep 4;169(7):467 73.
31. World Health Organization. Physical activity. 2004. https://www.who.int/initiatives/behealthy/physical-activity
32. May CR, Johnson M, Finch T. Implementation, context and complexity. Implementation Science [Internet]. 2016 Oct 19 [cited 2024 Jul 16];11(1). Available from: https://www.ncbi.nlm.nih.gov/pmc/articles/PMC5069794/
33. Pollock et al., 2023. Pollock D, Peters MDJ, Khalil H et al. Recommendations for the extraction, analysis, and presentation of results in scoping reviews. JBI Evid Synth. 2023 Mar 1;21(3):520-32.
34. Allegri N, Rosi A, Del Signore F, Cremascoli R, Cappa S, Tassorelli C, et al. Dementiafriendly intervention for hospitalized older adults with cognitive impairments: results of the Italian Dementia-Friendly Hospital Trial (IDENTITÀ). Aging & Mental Health. 2021 Mar 24;1 9.
35. Apolinario D, Yagamuti STF, Dutra AF, de Sousa Lara EM, de Cassia Pires Coli R, & Weber B. Safe hospital for the elderly program: An observational study on the impact in reducing functional decline. *Cad. Saúde Pública*. 2022;*38*(2), 1 16
36. Bakker FC, Persoon A, Sebastian J.H. Bredie, Jolanda van Haren-Willems, Leferink VJ, Luc Noyez, et al. The CareWell in Hospital program to improve the quality of care for frail elderly inpatients: results of a before after study with focus on surgical patients. 2014 Nov 1;208(5):735 46.
37. Bryant EA, Tulebaev S, Castillo-Angeles M, Moberg E, Senglaub SS, O Mara L, et al. Frailty Identification and Care Pathway: An Interdisciplinary Approach to Care for Older Trauma Patients. Journal of the American College of Surgeons. 2019 Jun;228(6):852859.e1.
38. Cohen Y, Zisberg A, Chayat Y, Gur-Yaish N, Gil E, Levin C, et al. Walking for Better Outcomes and Recovery: The Effect of WALK-FOR in Preventing Hospital-Associated Functional Decline Among Older Adults. Newman A, editor. The Journals of Gerontology: Series A. 2019 Mar 2;74(10):1664 70.
39. Heim N, Rolden H, van Fenema EM, Weverling - Rijnsburger AWE, Tuijl JP, Jue P, et al. The development, implementation and evaluation of a transitional care programme to improve outcomes of frail older patients after hospitalisation. Age and Ageing. 2016 Jun 13;45(5):642 50.
40. Inouye SK, Bogardus ST, Charpentier PA, Leo-Summers L, Acampora D, Holford TR, et al. A Multicomponent Intervention to Prevent Delirium in Hospitalized Older Patients. New England Journal of Medicine [Internet]. 1999 Mar 4 [cited 2020 Jan 7];340(9):669

76. Available from: https://www.nejm.org/doi/full/10.1056/NEJM199903043400901

41. Martínez-Velilla N, Garrués-Irisarri M, Ibañez-Beroiz B, Gil-Cabanas J, Richarte-García A, Idoate-Saralegui F, de Paz PC, Cambra K. An exercise program with patient s involvement and family support can modify the cognitive and affective trajectory of acutely hospitalized older medical patients: a pilot study. Aging Clinical and Experimental Research. 2016 Jun;28:483-90.
42. Naylor MD, Hirschman KB, Hanlon AL, Bowles KH, Bradway C, McCauley KM, et al.

Comparison of evidence-based interventions on outcomes of hospitalized, cognitively impaired older adults. Journal of Comparative Effectiveness Research. 2014 May;3(3):245 57.

43. Mudge AM, Giebel AJ, Cutler AJ. Exercising Body and Mind: An Integrated Approach to Functional Independence in Hospitalized Older People. Journal of the American Geriatrics Society. 2008 Apr;56(4):630 5.
44. Mudge AM, McRae P, Donovan PJ, Reade MC. Multidisciplinary quality improvement programme for older patients admitted to a vascular surgery ward ^*^. Internal Medicine Journal. 2020 Jun;50(6):741 8.
45. VidÃ¡n MT, SÃ¡nchez E, Alonso M, Montero B, Ortiz J, Serra JA. An Intervention Integrated into Daily Clinical Practice Reduces the Incidence of Delirium During Hospitalization in Elderly Patients. Journal of the American Geriatrics Society [Internet].

2009 Nov [cited 2019 Sep 16];57(11):2029 36. Available from: https://onlinelibrary.wiley.com/doi/10.1111/j.1532-5415.2009.02485.x

46. Suwanpasu S, Pongpaew W, Praparpak W. Geriatric resource nurse programme to maintain functional status of elderly patients hospitalised for community-acquired pneumonia. Asian Journal of Gerontology & Geriatrics. 2015 Dec;10(2):10.
47. Boltz M, Resnick B, Chippendale T, Galvin J. Testing a Family-Centered Intervention to Promote Functional and Cognitive Recovery in Hospitalized Older Adults. Journal of the American Geriatrics Society. 2014 Dec; *62*(12):2398 2407
48. Chang E, Hancock K, Hickman L, Glasson J, Davidson P. Outcomes of acutely ill older hospitalized patients following implementation of tailored models of care: A repeated measures (pre- and post-intervention) design. International Journal of Nursing Studies. 2007 Sep;44(7):1079 92.
49. Hamilton L, Lyon PS. A Nursing-Driven Program to Preserve and Restore Functional Ability in Hospitalized Elderly Patients. JONA: The Journal of Nursing Administration. 1995 Apr;25(4):30 7.
50. Holt R, Young J, Heseltine D. Effectiveness of a multi-component intervention to reduce delirium incidence in elderly care wards. Age and Ageing. 2013 Aug 26;42(6):721 7. 51. Inouye SK, Wagner DR, Acampora D, Horwitz RI, Cooney LM, Tinetii ME. A Controlled Trial of a Nursing-Centered Intervention in Hospitalized Elderly Medical Patients: The Yale Geriatric Care Program. Journal of the American Geriatrics Society. 1993 Dec;41(12):1353 60.
52. Inouye SK, Bogardus ST, Baker DI, Leo-Summers L, & Cooney, L. M. The hospital elder life program: A model of care to prevent cognitive and functional decline in older hospitalized patients. Journal of the American Geriatrics Society. 2000;*48*(12):1697 1706.
53. Kratz T, Heinrich M, Schlauß E, Diefenbacher A.Preventing postoperative delirium. Deutsches Ärzteblatt International, 2015;*112*:289 296.
54. Liu B, Moore JE, Almaawiy U, Chan WH, Khan S, Ewusie J, et al. Outcomes of Mobilisation of Vulnerable Elders in Ontario (MOVE ON): a multisite interrupted time series evaluation of an implementation intervention to increase patient mobilisation. Age and Ageing. 2017 Jul 15;47(1):112 9.
55. Milisen K, Foreman MD, Abraham IL, De Geest S, Godderis J, Vandermeulen E, et al. A Nurse-Led Interdisciplinary Intervention Program for Delirium in Elderly Hip-Fracture Patients. Journal of the American Geriatrics Society. 2001 May;49(5):523 32.
56. Peyrusqué E, Buckinx F, Bolduc A, Law C, Kergoat MJ, Aubertin-Leheudre M. Potential Efficacy of Pragmatic Exercise Program (Sprint) During Hospitalization in Older Adults on Health Care and Physical Performance: A Pilot Study. The Journal of nutrition, health & aging. 2020 Oct 12; *25*(1):126 133.
57. Von Renteln-Kruse W, Krause T. Incidence of In-Hospital Falls in Geriatric Patients

Before and After the Introduction of an Interdisciplinary Teamâ€ “ Based Fall-

Prevention Intervention. Journal of the American Geriatrics Society. 2007 Dec;55(12):2068 74.

58. Rubin FH, Neal K, Fenlon K, Hassan S, Inouye SK. Sustainability and Scalability of the Hospital Elder Life Program at a Community Hospital. Journal of the American Geriatrics Society. 2011 Feb;59(2):359 65.
59. Wanich CK, Sullivan-Marx EM, Gottlieb GL, Johnson JC. Functional Status Outcomes of a Nursing Intervention in Hospitalized Elderly. Image: the Journal of Nursing Scholarship. 1992 Sep;24(3):201 8.
60. Wand APF, Thoo W, Sciuriaga H, Ting V, Baker J, Hunt GE. A multifaceted educational intervention to prevent delirium in older inpatients: A before and after study. International Journal of Nursing Studies [Internet]. 2014 Jul [cited 2019 Nov 21];51(7):974 82. Available from: https://www.sciencedirect.com/science/article/abs/pii/S0020748913003556
61. Gil E, Zisberg A, Efrat Shadmi, Nurit Gur-Yaish, Ksenya Shulyaev, Yehudith Chayat, et al. Still WALKing-FOR: 2-year sustainability of the WALK FOR intervention. Age and ageing. 2023 Jun 1;52(6).
62. Miller J, Campbell J, Moore K, Schofield A. Elder Care Supportive Interventions Protocol: Reducing Discomfort in Confused, Hospitalized Older Adults. Journal of Gerontological Nursing. 2004 Aug 1;30(8):10 8.
63. Gazineo D, Godino L, Decaro R, Calogero P, Pinto D, Chiari P, et al. Assisted Walking Program on Walking Ability in In Hospital Geriatric Patients: A Randomized Trial. Journal of the American Geriatrics Society. 2020 Nov 12;69(3):637 43.
64. Mudge AM, McRae P, Banks M, Blackberry I, Barrimore S, Endacott J, et al. Effect of a Ward-Based Program on Hospital-Associated Complications and Length of Stay for Older Inpatients. JAMA Internal Medicine. 2022 Mar 1;182(3):274.
65. Mudge A, McRae P, Young A, Blackberry I, Lee Steere K, Barrimore SE, et al. Implementing a ward-based programme to improve care for older inpatients: process evaluation of the cluster randomised CHERISH trial. BMC Health Services Research. 2023 Jun 21;23(1).
66. Rodrigues C, Mendonça D, Martins MM. Effects of a nursing care program focused on basic self-care in older acute medical in-patients: a randomized controlled trial. Porto Biomedical Journal. 2020 Nov;5(6):e086.
67. Wang YY, Yue JR, Xie DM, Carter P, Li QL, Gartaganis SL, et al. Effect of the Tailored, Family-Involved Hospital Elder Life Program on Postoperative Delirium and Function in Older Adults. JAMA Internal Medicine. 2020 Jan 1;180(1):17.
68. King BJ, Steege LM, Winsor K, VanDenbergh S, Brown CJ. Getting Patients Walking: A Pilot Study of Mobilizing Older Adult Patients via a Nurse-Driven Intervention. Journal of the American Geriatrics Society. 2016 Aug 22;64(10):2088 94.
69. Juneau A, Bolduc A, Nguyen P, Leclerc BS, Rousseau J, Dubé F, et al. Feasibility of Implementing an Exercise Program in a Geriatric Assessment Unit: the SPRINT Program. Canadian Geriatrics Journal. 2018 Sep 14;21(3):284 9.
70. Zisberg A, Agmon M, Gur-Yaish N, Rand D, Hayat Y, Gil E. No one size fits all	the development of a theory-driven intervention to increase in-hospital mobility: the WALK-FOR study. BMC Geriatrics. 2018 Apr 13;18(1).
71. Inouye SK, Baker DI, Fugal P, Bradley EH. Dissemination of the Hospital Elder Life Program: Implementation, Adaptation, and Successes. Journal of the American Geriatrics Society. 2006 Oct;54(10):1492 9.
72. World Health Organization. Nursing and Midwifery; 2022.

https://www.who.int/news-room/fact-sheets/detail/nursing-and-midwifery.

73. Butler R, Monsalve M, Thomas GW, Herman T, Segre AM, Polgreen PM, et al. Estimating Time Physicians and Other Health Care Workers Spend with Patients in an Intensive Care Unit Using a Sensor Network. The American Journal of Medicine [Internet]. 2018 Aug;131(8):972.e9 15. Available from: https://www.ncbi.nlm.nih.gov/pubmed/29649458
74. World Health Organization. (2010) Framework for action on interprofessional education and collaborative practice. Geneva, Switzerland: WHO. https://iris.who.int/bitstream/handle/10665/70185/WHO_HRH_HPN_10.3_eng.pdf?sequ ence=1
75. Yi Chia Wei, Heather C, Wen Yi Huang, Yao Liang Chen, Lin C, Chih Ken Chen, et al. White Matter Integrity Underlies the Physical-Cognitive Correlations in Subjective Cognitive Decline. Frontiers in Aging Neuroscience. 2021 Aug 2;13.
76. Fox MT, Sidani S, Butler JI, Skinner MW, Macdonald M, Durocher E, et al. Optimizing hospital-to-home transitions for older persons in rural communities: a participatory, multimethod study protocol. Implementation Science Communications. 2021 Jul 22;2(1).
77. Lennox, L., Maher, L., & Reed, J. (2018). Navigating the sustainability landscape: A systematic review of sustainability approaches in healthcare. Implementation Science,

13(27), 1-17. DOI: 10.1186/s13012-017-0707-4

78. Lanham HJ, Leykum LK, Taylor BS, McCannon CJ, Lindberg C, Lester RT. How complexity science can inform scale-up and spread in health care: understanding the role of self-organization in variation across local contexts. Social science & medicine. 2013 Sep 1;93:194-202.
79. Dermody G, Kovach CR. Nurses Experience With and Perception of Barriers to Promoting Mobility in Hospitalized Older Adults: A Descriptive Study. Journal of Gerontological Nursing. 2017 May 30;43(11):22 9.
80. Fox MT, Butler JI. Nurses perspectives on how operational leaders influence functionfocused care for hospitalised older people. Journal of Nursing Management. 2016 Sep 16;24(8):1119 29.
81. Hower KI, Vennedey V, Hillen HA, Kuntz L, Stock S, Pfaff H, et al. Implementation of patient-centred care: which organisational determinants matter from decision maker s perspective? Results from a qualitative interview study across various health and social care organisations. BMJ Open [Internet]. 2019 Apr;9(4):e027591. Available from: https://bmjopen.bmj.com/content/9/4/e027591
82. Sepulveda-Pacsi AL, Soderman M, Kertesz L. Nurses perceptions of their knowledge and barriers to ambulating hospitalized patients in acute settings. Applied Nursing Research. 2016 Nov;32:117 21.
83. Yous ML, Ploeg J, Kaasalainen S, Martin LS. Nurses Experiences in Caring for Older Adults with Responsive Behaviors of Dementia in Acute Care. SAGE Open Nursing [Internet]. 2019 Jan;5. Available from: https://journals.sagepub.com/doi/full/10.1177/2377960819834127
84. Parke B, Boltz M, Hunter KF, Chambers T, Wolf-Ostermann K, Adi MN, et al. A Scoping Literature Review of Dementia-Friendly Hospital Design. The Gerontologist. 2016 Nov 9;57(4):gnw128.

S-1 **Table:** *Preferred Reporting Items for Systematic reviews and Meta-Analyses extension for Scoping*

*Reviews (PRISMA-ScR) Checklist*

S-2 **Table:** *Table of Included Studies (N=38)*
S-3 **Table**: *Search Strategies for MEDLINE*
S-4 **Table**: *Inclusion and Exclusion Criteria*
S-5 **Figure:** *Preferred Reporting Items for Systematic Reviews and Meta-Analyses (PRISMA)*

*Flow Diagram*

660 Table 1.
661 *Details on Focus of Educational Strategies Utilized by Studies*

S. N First	HCPs included in	Topics covered.	Education author	education	(e.g., delirium, mobility)	strategies used

(Year)	(e.g., in-service,

workshops, pockets cards)

1. Allegri et Yes: Staff (doctors, Dementia, cognitive Training using al. (2022) nurses, physical impairment, depression teaching modules.

therapists and	bipolar psychotic

healthcare assistants).	disorder, delirium, pain,

communication and interaction with patients

2. Apolinario	Yes (nurses,	Fall, pressure injury,	Education targeted

et al.	physiotherapist, speech bronchoaspiration, delirium,	to train

(2022)	therapist, nutritionist,	mechanical restraint,	professionals from

psychologist, and malnutrition, medication different areas. clinical iatrogenesis, and functional Staff were given pharmacologist). decline. resources such as

booklets, video

lessons, infographics, onsite small group training, and training workshops.

| 3. | Bakker et al. (2014). | Yes (Nurses and Physicians). | Delirium, physical decline, fall, frailty screening, medical review, geriatric assessment, stimulation of cognitive and physical activities. | Educational session, on the job coaching. |
| --- | --- | --- | --- | --- |
| 4. | Boltz et al.  (2014) | Yes (Nurses). | Prevention and management of delirium and functional decline; assessment of mood, cognition, and function; incorporation of function-focused care into care routines; ways to communicate, partnering, and motivating patients and family; and discharge planning. | Education session. |

5. Bryant et Yes (nurses, ancillary Delirium prevention, Educational al. (2019) staff, residents, mobility, medication presentation,

physician assistants management/prognostic pocket card, and attending staff assessment, reducing other mandatory who frequently care complications, social educational for older patients in needs/goal setting and injury modules. trauma unit and in the prevention.

ICU).

6. Chang et al Yes (nurses).	Mobility.	In-service.

(2007)

7. Cohen et al Yes (Nurses, nurse	Mobility.	Theoretical and

(2019)	aides).	practical training.

8. Gazineo et	Yes (only one nurse).	Mobility.	Training.

al. (2021)

9. Gill et al	Yes (unit staff	Mobility.	Theoretical and

(2023)	including nurses, nurse	practical training.

aides and physical

therapists).

10. Hamilton	Yes (nurses).	Mobility.	Staff education.

& Lyon

(1995)

11. Heim et al.	Yes (nurses and	Frailty, ADL functioning.	On the job

(2016).	medical staff).	training.

12. Holt et al.	Yes (ward staff	Delirium.	Interactive lecture,

(2013)	including staff nurses	handout, quiz, and healthcare	poster, reference assistants).	materials and case

vignettes.

13 Inouye et Yes (Nurses). Geriatric care (Delirium, Education, al. (1993b) functional impairment, training, lecture

incontinence and pressure	series.

sores).

14. Inouye et	Yes (Research and	Delirium risk factors:	Training.

al. (1999)	clinical nurses).	cognitive impairment, sleep

deprivation, immobility, visual impairment, hearing impairment and dehydration.

15. Inouye et	Yes (Nurses and	Mobility, vision/hearing,	Formal didactic al. (2000)	Physicians) and	volume repletion, feeding	sessions, small

volunteers.	assistance, sleep.	group training, and

resource materials.

16. Inouye et	Unsure (volunteers).	Delirium.	Regular education

al. (2006)	sessions

17. Juneau et Yes (Nurses, Mobility. Training on al. (2018) physicians and exercise program.

physiotherapist).

18. King et al.	Yes (Nurses and	Mobility.	Training.

(2016).	certified nursing

assistants).

19. Kratz et al.	Yes (Nurses).	Delirium.	Training.

(2015)

20. Liu et al.	Yes (Physicians,	Mobility.	Education

(2018)	nurses).	modules.

21. Martinez-	Yes (ward and	Mobility and functional	Education.

Velila et	research team staff).	independence.

al. (2016)

22. Milisen et	Yes (Nurses).	Delirium, depression, and	Educational

al. (2001).	dementia.	poster, training

23. Miller et	Yes (Nurses and	Confusion, discomfort,	Education

al. (2004)	undergraduate nursing	physical care, assessment of	program.

students).	basic needs, patient safety

and therapeutic communications.

24. Mudge et	Yes (Nurses).	Functional independence	Education, group

al. (2008)	and mobility	in-services

25. Mudge et	Yes (Nurses or allied	Nutrition and hydration,	Training, videos.

al. (2022).	health professional).	mobility, meaningful

cognitive and social engagement and in multidisciplinary teamwork.

26. Mudge et	Yes (allied health	Nutrition and hydration,	Training.

al. (2020).	assistants).	mobility, meaningful

cognitive and social engagement and in multidisciplinary teamwork.

27. Mudge et	Yes (allied health or	Nutrition and hydration,	Training including al. (2023)	nursing assistants).	mobility, meaningful	didactic and

cognitive and social	interactive engagement and in	content.

multidisciplinary teamwork.

28. Naylor et	Yes (nurses and	Cognitive deficit.	Web based

al. (2014).	advanced practice	modules,

nurses).	seminars.

29. Peyrusque	Yes (nurses,	Mobility.	Group training.

et al.	physicians,

(2021)	physiotherapists).

30. Rodrigues	Yes (nurses and	Mobility, nutrition.	Training and

et al.	nursing assistants).	regular education

(2020).	sessions.

31. Rubin et	Unsure (Volunteers).	Nutrition, delirium, overall	Training program.

al. (2011)	care of frail older adults.

32. Suwanpasu Yes (nurses).	Sleep disorder, problem with	Training program.

et al.	eating or feeding,

(2015)	incontinence, confusion,

mobility, falls, and skin breakdown.

33. Vidan et	Yes (nurses).	Delirium.	Educational

al. (2009)	session, poster,

cards with recommendations included in the first page of the treatment book.

34. von	Yes (Nurses,	Mobility.	Presentations,

Renteln-	therapeutic staff, and	training.

Kruse &	physicians).

Krause

(2007).

35. Wand et	Yes (nurses, medical	Delirium.	Ward based in-

al. (2014).	staff).	services,

workshops,

tutorials, education session, posters.

36. Wang et	Yes (Medical	Delirium.	Training.

al. (2020)	postgraduates,

majoring in geriatrics or geriatric nursing were trained as coordinators, who took charge of delivering the intervention plan to nurses, surgical doctors and other team

staff).

37. Wanich et	Yes (Nurses).	Delirium.	In-service

al. (1992)	program.

38. Zisberg et	Yes (Nurses, nurse	Mobility.	Online tutorials,

al. (2018).	aides).	face to face

training.

662

| o et al.  (2022) | Apolinar  i |  | al. (2022) | Alleg  ri et |  |  |  |  | **Study** |
| --- | --- | --- | --- | --- | --- | --- | --- | --- | --- |
|  |  |  |  |  | Large |  | **ion** | **intervent** | **Level of** |
| ***** |  |  | ***** |  | Medium |  |  |  |  |
|  |  |  |  |  | Small |  |  |  |  |
| I  P |  |  | N |  | Multifocal geriatric protocols |  |  | (  Nurse only (N); Interprofessional (IP  )) | **Care process changes** |
|  |  |  |  |  | Mobility  interventions |  |  |  |  |
| I  P |  |  |  |  | Family involvement |  |  |  |  |
|  |  |  | N |  | Cognitive interventions |  |  |  |  |
|  |  |  |  |  | Continence interventions |  |  |  |  |
|  |  |  | N |  | Pain interventions |  |  |  |  |
|  |  |  | N |  | Medication review/protocol |  |  |  |  |
|  |  |  |  |  | Pressure ulcer prevention |  |  |  |  |
|  |  |  |  |  | Nutrition/Dehydration |  |  |  |  |
|  |  |  |  |  | Sleep protocol |  |  |  |  |
|  |  |  |  |  | Physical Function/Falls |  |  |  |  |
| * |  |  |  |  | Geriatric/Frailty screening |  |  |  |  |
| I  P |  |  |  |  | Geriatric p  atient rounds |  |  |  |  |
|  |  |  |  |  | Volunteers |  |  |  |  |
|  |  |  |  |  | Walking trails |  |  | **changes** | **Environment** |
|  |  |  |  |  | Orientation (clocks) |  |  |  |  |
|  |  |  |  |  | Modifications/Declutter |  |  |  |  |
|  |  |  |  |  | Adjusting seating/bed height |  |  |  |  |
|  |  |  |  |  | Sensory and mobility aids |  |  |  |  |
|  |  |  |  |  | Equipment purchase |  |  | **Supp**  **o**  **rt** | **Administrative** |
|  |  |  |  |  | Additional staffing |  |  |  |  |
|  |  |  |  |  | Restraint use/policy |  |  |  |  |
|  |  |  |  |  | Patient/family in shift report |  |  |  |  |
|  |  |  |  |  | Electronic record  modifications |  |  |  |  |
|  |  |  |  |  | Regional Intersectoral |  |  |  |  |
|  |  |  |  |  | Advanced practice nurse |  |  |  | **Facilitation/Leadership** |
|  |  |  |  |  | Geriatric resource nurse/staff |  |  |  |  |
| * |  |  |  |  | Nurse supervisor/case manager |  |  |  |  |
|  |  |  |  |  | Geriatrician |  |  |  |  |
|  |  |  |  |  | IP resource person |  |  |  |  |
|  |  |  |  |  | IP geriatric team |  |  |  |  |
|  |  |  |  |  | IP family meetings |  | **Planning** | **Transition** | **Discharge/** |
| * |  |  |  |  | Links to discha  rge settings |  |  |  |  |
|  |  |  |  |  | Minimum data set/digital info |  |  |  |  |
|  |  |  |  |  | Patient follow up |  |  |  |  |
| ADL  -    Yes | **Physical:** | No | Maintained    **Cognitive** | **Physical** |  | **nt** | **improveme** | **outcomes** | **Functional** |

Table

2

.

663

*Inte*

*r*

*ventions in*

*A*

*ddition to*

*E*

*ducation*

664

SUPPORTING

GERONTOLOGICAL EDUCATION

| u et al.  (2015) | Suwanpas |  | Rubin et  al. (2011) | | Rodrigues  et al.  (2020). | Peyrusque  et al.  (2021) |  | al. (2014). | Naylor et |
| --- | --- | --- | --- | --- | --- | --- | --- | --- | --- |
|  |  |  |  | |  |  |  |  |  |
| * |  |  | * | | * |  |  |  |  |
|  |  |  |  | |  | * | * |  |  |
| N |  | P | I | | N |  |  |  |  |
| * |  |  | * |  | * | I  P |  |  |  |
|  |  |  | * |  |  | * |  |  |  |
| * |  |  | * |  | * |  | N |  |  |
| * |  |  |  |  |  |  |  |  |  |
|  |  |  |  |  |  |  |  |  |  |
|  |  |  |  |  |  |  |  |  |  |
| * |  |  |  |  |  |  |  |  |  |
| * |  |  | * |  | * |  |  |  |  |
| * |  |  | * |  |  |  |  |  |  |
| * |  |  |  |  |  |  |  |  |  |
|  |  |  |  |  |  |  |  |  |  |
| N |  |  |  |  |  |  |  |  |  |
|  |  |  |  |  |  |  |  |  |  |
|  |  |  |  |  | * |  |  |  |  |
|  |  |  |  |  | * |  |  |  |  |
|  |  |  | * |  | * |  |  |  |  |
|  |  |  |  |  |  |  |  |  |  |
|  |  |  |  |  |  |  |  |  |  |
|  |  |  |  |  |  | * |  |  |  |
|  |  |  |  |  |  |  |  |  |  |
|  |  |  |  |  |  |  |  |  |  |
|  |  |  |  |  |  |  |  |  |  |
|  |  |  |  |  |  |  |  |  |  |
|  |  |  |  |  |  |  |  |  |  |
| * |  |  | * |  |  |  |  |  |  |
|  |  |  | * |  |  |  | * |  |  |
|  |  |  |  |  |  |  |  |  |  |
|  |  |  |  |  |  |  |  |  |  |
|  |  |  | * |  |  |  |  |  |  |
| * |  |  | * |  |  | * |  |  |  |
|  |  |  |  |  |  |  |  |  |  |
|  |  |  |  |  |  |  |  |  |  |
|  |  |  |  |  |  |  |  |  |  |
|  |  |  |  |  |  |  | * |  |  |
| ADL No | **Physical** | Yes | Delirium | **Cognitive** | **Physical**    ADL    Yes | **Physical**    ADL,  Mobility    Yes | No | ADL/IADL | **Physical** |

SUPPORTING

GERONTOLOGICAL EDUCATION

INTERVENTIONS SUPPORTING GERONTOLOGICAL EDUCATION

666

43

Re pon e o Re ie er

# Response to Reviewer Comments Received Dec. 30th, 2024

**Manuscript ID:** PONE-D-24-35120

**Title:** Interventions Supporting the Translation of Gerontological Knowledge into Practice to

Improve Functional Outcomes for Hospitalized Older Adults: A Scoping Review

| **Comment** | **Response** | **Changes Made** |
| --- | --- | --- |
| **Journal Requirements** |  |  |
| Please ensure that your manuscript meets PLOS ONE's style requirements, including those for file  naming. The PLOS ONE style templates can be found at https://journals.plos.org/pl osone/s/file?id=wjVg/PL OSOne_formatting_sampl e_main_body.pdf and https://journals.plos.org/pl osone/s/file?id=ba62/PLO SOne_formatting_sample _title_authors_affiliations. pdf | We have ensured that the manuscript meets PLOS ONE's style requirements, including those for file naming. |  |
| We notice that your supplementary  [figures/tables] are included in the manuscript file. Please remove them and upload them with the file type 'Supporting Information'.  Please ensure that each Supporting Information file has a legend listed in the manuscript after the references list. | We have relocated the table  to the supplementary material  S-1 . | P. 43. |
| We note you have included a table to which you do not refer in the text of your manuscript. Please ensure that you refer to Table 4 in your text; if accepted, production will need this reference to link the reader to the Table. | We have updated references to the tables throughout the manuscript. |  |
| **Reviewer 1** |  |  |

| The paper chooses to review the papers that contain the element of education for staff. In addition to knowledge deficit, there are numerous factors of why there were insufficient interventions that aimed at physical and cognitive functions. No sufficient reasons and theory support were described to backup the choice of focusing on education. While a typical staff training that aims at improving functional outcomes usually involves staff education, such choice led to authors excluding papers that did not elaborate staff knowledge training. More justifications are needed to support this choice. | We now explain in the introduction why we focused on education. | P. 5. We now indicate that  The implementation of function-oriented approaches, however, has been hampered by inadequate gerontological knowledge on the part of healthcare professionals, which explains our focus on education as a starting point in this review. |
| --- | --- | --- |
| More quantitative findings could be cited in the introduction section to show the adverse effects of acute hospital in worsening physical and cognitive function, and thereby highlight the importance of the paper. | We have integrated several recent sources substantiating the adverse effects of acute hospitalization on physical and cognitive function. | P. 3. We now indicate that  P  hospitalized older adults is highly limited^4, 5^ and loss of ability to perform activities of daily living (ADL) is a common problem. Almost  one third of this patient population experience functional decline at discharge.^6^ Crucially, if they do not return to their prehospitalization level of function within the 30 days of discharge, they are at high risk for functional decline becoming permanent.^7^ |
| The research priority mentioned on line 94 can be moved to the introduction section. | This line has been moved to the introduction section. | P. 3. |
| More elaborations of the terms in the introduction | See above. We have clarified  that we mean an | P.4. We now state that  I |

| section are desirable, e.g.      62,  80 . | lens from implementation sc .                We have also clarified that wide array of    examples. | lens from implementation science, we conceptualize intervention implementation as influenced by determinants at multiple levels (micro, meso, and macro), which must be addressed conjointly.    P. 5.  wide array of micro, meso and macro factors factors (e.g. patient attitudes, organizational culture/climates, financial resources, leadership)^9^ shape |
| --- | --- | --- |
| As per my understanding, acute hospital focuses on short-term medical treatment. For each study, for how long do the participants stay in the hospital and receive the interventions? I wonder if the focus of acute hospital interventions should be    functional outcomes as stated on line 107 due to the short-term nature of hospital stay. | Length of stay was not consistently reported on across studies, so we are not able to systematically extract  this information. We have replaced with    explanation. | P. 4. We now indicate that  I ,  healthcare professionals  endeavour to maintain older ioning at their  pre-hospitalization baseline,  to avoid functional losses  . |
| On line 122-124,  search focused on physical function and BADL, but both physical and cognitive outcomes were abstracted from any  .  Please explain this in the introduction section. | Although our search focused on physical function BADL, both physical and cognitive outcomes were abstracted because evidence indicates that attending to physical function and BADL can also support cognition. Accordingly, if cognitive function was measured and reported on, we included it in our abstraction to lend context to the physical domains that were measured. | P. 7. |

| What are the major physical and cognitive function domains and measures among the papers reviewed? | We now summarize the major physical and cognitive function domains and measures included in the papers reviewed. | P. 12. We now indicate that    domains explored were related to mobility, dexterity, and ADL. The major cognitive function domains examined domains including orientation to time and place, recall, etc. The most common measures included the Barthel  Index, Katz Activities of  Daily Living Scale, Mini Mental State Exam (MMSE), and the Montreal Cognitive Assessment (MoCA). |
| --- | --- | --- |
| The intervention categories did not seem to be discussed in detail in discussion section.      line 243? Please explain in sufficient detail to show that this is the objective of the paper as stated in the  Objective section. | We now discuss the intervention categories in the discussion in greater detail. | P. 13.  The educational interventions that supported functional outcomes for hospitalized older adults in this review focused on changes to care processes (e.g. multifocal protocols, mobility interventions, and cognitive interventions), environmental changes (e.g. walking trails, clocks, decluttering), administrative support (e.g.  purchase of equipment, electronic record modification), facilitation/leadership (e.g. geriatricians and specialized nurses), and discharge/transition planning. |
| Nurses as primary interventionist vs  I    in the first paragraph in discussion section. What are the professions in | We now more clearly define  I using a WHO definition. | P. 13. We now explain that an interprofessional approach  multiple  health workers from different professional backgrounds working together with patients, families, carers and communities to deliver the highest possible quality care |

| I  ? |  | across settings. The professionals involved differ on a case-by-case basis, but in the acute care context, this would typically entail physicians, nurses, and allied health professionals (e.g. physiotherapists, occupational therapists, dieticians etc.). |
| --- | --- | --- |
| How are the division of roles and cooperation among the professions in the papers reviewed? | We explain in the methods that the division of professional roles in the studies included in our scoping review were analyzed using the same inductive, descriptive approach described in our analysis section in the methods. | P. 9. We now indicate that we used our descriptive  summary to map the  educational interventions/practice change initiatives, their purposes, the division of interprofessional roles, as well as the outcomes examined and how they were  . |
| The third paragraph in the discussion section concerns sustainability but elaborate if some interventions were expanded to other hospitals. Please define sustainability. | We now include a definition of sustainability. | P. 14. We now indicate that    how the intervention would  be maintained after the study  (L ., 2018). |
| **Reviewer 2** |  |  |
| Title  - To simplify/clarify the  : I  Supporting  Implementation of  Gerontological  Education for  Healthcare  Professionals to  Improve Functional  Outcomes for  Hospitalized Older Adults: A Scoping  R ; :  interventions supporting gerontological | We have simplified/clarified the title. | P. 1. The title now reads  Interventions Supporting the Translation of Gerontological  Evidence into Practice to  Optimize Functional  Outcomes for Hospitalized  Older Adults: A Scoping Review. |

| education for healthcare professionals to  OR  interventions supporting the translation of gerontological knowledge into practice to improve functional  OR  Interventions  Supporting the Implementation of  healthcare    Gerontological knowledge to Improve |  |  |
| --- | --- | --- |
| Abstract: |  |  |
| L.30: examine what?  Authors may want to    or type of | This change has been implemented. | P. 2. |
| L40: Interventions included in addition to educational strategies, | This change has been implemented. | P. 2. |
| L40: environmental changes and leadership roles. | This change has been implemented. | P. 2. |
| Add relevant key words to facilitate retrieval of the study (may include healthcare professionals, implementation science) | Additional keywords    have now been included. | P. 2. |
| Add source of evidence to the abstract (as per Prisma) | We now explain in the abstract that was primary studies were the source of evidence. | P. 2. We now indicate that  Target studies were primary research studies that |
| Words describing the main examined concept should be unified and maintained throughout the manuscript starting | We have ensured consistent language across the different section of the manuscript. To enhance clarity, we have | P. 2. We now indicate that    was to examine the characteristics or type of interventions supporting the |

| with the title and especially when talking about aims and objectives in different sections to avoid misleading the readers.  When I first read the  , I  sure whether the focus was merely on educational interventions or other supportive strategies to implement knowledge and improve functional outcomes. It is until the results section that things got clear. It    supporting educational interventions for healthcare professionals to improve functional outcomes among hospitalized older  OR  nterventions  supporting gerontological education for healthcare professionals to  RA HER  HAN  that have been used to support the implementation of gerontological knowledge by hospitalbased healthcare professionals and/or interprofessional teams    (  abstract) | implemented the same phrasing as the title. | translation of gerontological knowledge into practice to  improve functional outcomes for . |
| --- | --- | --- |
| - L34:  examined initiatives or | This change has been implemented. | P. 2. We now indicate that Included studies were |

| care delivery models to improve functional  :  be removed and keep in  L35: I  described an  educational intervention for healthcare professionals as part of a wider initiative to promote practice change and assessed functional outcomes (physical and/or  ) . |  | primary research studies that described an educational intervention for healthcare professionals as part of an initiative to promote practice change and assessed functional outcomes (physical  / ). |
| --- | --- | --- |
| Introduction:  - Justify the use of the ScR approach; why authors did not choose other approaches? As per Prisma checklist  P .,  objectives of this review should be aligned with those of the ScR approach:  E  review questions/objectives lend themselves to a scoping review  . (  instance: explore the breadth or depth of the literature, map and summarize the evidence, inform future research, and identify or address knowledge gaps, heterogeneous literature, precursor of systematic review, identify key characteristics or factors related to the concept). | A justification for our SCR approach was included in the methods section and has now been moved up to the introduction section to contextualize the objectives. | P. 6. |

| Methods  - L92  steps for scoping reviews recommended  P .  to say guidelines as this reference does not include steps. It rather clarifies guidelines related to ScR conduct, in line with PRISMAScR. | This change has been implemented. | P. 6. |
| --- | --- | --- |
| L93 O  sought to understand how healthcare    gerontological knowledge can be improved and applied, one of the research priorities identified in a provincial priority-  :  wording of the ScR aim should be consistent. While it was in the aim  , what interventions for hospital-based healthcare professionals and/or interprofessional teams, in addition to gerontological education, have been implemented to    functional outcomes in  , ~~here~~  ~~healthcare~~    ~~gerontological~~ ~~knowledge can be~~ ~~improved and applied~~ . Those are two different research questions. | We have ensured that the wording of the ScR aim is consistent throughout the manuscript. |  |

| Line 95-102:  justification for the use of ScR approach: I suggest this is removed to the introduction section, to anchor the choice of the approach in the available literature, what do we know about the topic and what could this review add. Align the objective of this study with the objectives of the ScR itself. How could ScR help to meet the study aims? | This paragraph has been relocated to the introduction/objectives section. | P. 6. We now indicate that  As we could not locate any existing review in the hospital setting, we determined that a broader scoping review to map the breadth of the available evidence (as opposed to a more focused systematic review) was most appropriate. |
| --- | --- | --- |
| L104: refer to th availability of the checklist as supplementary file. | This change has been implemented. | We now indicate that readers should (See S-2 Table). |
| Inclusion in L106 (all designs) contradicts L11 (excluded reviews): add to L106 all designs of primary research | This change has been implemented. | P. 6. We now indicate that we  We included primary studies  . |
| L106: there is a need to explain how studies with different languages were handled (translation?  Multilingual authors?) | Non-English sources were translated using Google Translate. | P. 6. We now indicate that    not published in English were translated using were  translated using Google  Tr |
| L112 65  justify here the cutoff age (used by WHO as explained in later sections) | We selected 65+ as our cutoff because this a common cutoff used in the literature (e.g. in many WHO studies. | P. 6. We now indicate that  65+ (in line with the age cutoff used in many World Health Organization reports) in acute care hospital settings. |
| L113: specify  (supplementary table 3) | This change has been implemented. | P. 6. |
| L119: search strategy:  add here the main examined concept: ducational  . | Our search strategy was guided an experienced by a medical librarian. We have | P. 7. We now indicate that  N  derived from three main concepts: 1) educational interventions, 2) older |

| concept was mentioned in the Medline strategy; however, the population (PCC mnemonic) was absent (health care professionalsinterprofessional teams). The cognitive function was also absent but authors mentioned this as a limitation. | concept. We will consider the population (PCC mnemonic) in our searches for future scoping reviews. | hospitalized patients, and 3) Basic Activities of Daily  L (BADL) |
| --- | --- | --- |
| L115-139: the review is not blinded so pay attention to add authors initials. | Author initials have been added. |  |
| Results  - L185: suggest to  " | This change has been implemented. | P. 10. |
| L188: managerial intervention, leadership or facilitation roles | This change has been implemented for leadership or facilitation roles, but we prefer our phrasing  /  to managerial intervention. | PP. 10, 12. |
| L201: medium scale interventions and not studies | This change has been implemented. | P. 10. |
| L201:  focused | This change has been implemented. | P. 10. |
| Table 1 include details about the studies and is lengthy; authors may decide to include a summarized table in the text and add this one as supplementary file. | The table is now included as supplementary file S-2 Table. |  |
| Discussion  - S :  grounded our study in a robust theoretical  :  explained-registration link not accessible | We now explain that we are referring to our ecological conceptualization. We have updated the OSF registration link. | P. 16. We now indicate that study in a robust, context-  sensitive theoretical conceptualization drawn from  ecological implementation  . |

| L309: referring to 65+ as a WHO definition of older age is not accurate, even in the cited reference. UN defines older people as aged over 60. WHO use 60+ in some reports and 65+ in others. Better to say 65+ as a cutoff age used in many WHO reports. | We now indicate 65+ as used in many WHO reports. | PP. 6, 16. |
| --- | --- | --- |
| Supplementary files: - At the end of references add all supplementary files (search strategy, inclusion criteria table, and PRISMA flow  diagram in addition to the PRISMA checklist) | This change has been implemented. | PP. 24. |
| Prisma flow diagram: o reverse first arrow to the right. | We believe the arrow directions are accurate. |  |
| If 4482 are removed at the level of title and abstract screening, what are the 3921 removed  first? Are they duplicates? | The 4482 papers were removed because they were not relevant, which is now specified in the PRISMA Flow Diagram. | S-1 Figure. We now indicate  S  ( = 4482) |
| The box of excluded studies (screening level) is incomplete. The rest might include other exclusion causes like not describing the education intervention (as mentioned earlier in the text). | We have corrected the box on excluded studies. | S-1 Figure. |
