# Supplementary material for: Interventions supporting the translation of gerontological evidence into practice to optimize functional outcomes for hospitalized older adults: A scoping review
Source: PLoS One. 2025 Jun 16;20(6):e0324953. doi: 10.1371/journal.pone.0324953 (PMC12169582; doi:10.1371/journal.pone.0324953)
Supplement: Attachment — Submitted filename: PLos_One_Scoping Review_Response to Reviewers_27_02_2025.docx [file pone.0324953.s010.docx]

**Response to Reviewer Comments Received Feb. 24th, 2025**

**Manuscript ID:** PONE-D-24-35120

**Title:** Interventions Supporting the Translation of Gerontological Evidence into Practice to Optimize Functional Outcomes for Hospitalized Older Adults: A Scoping Review

| **Comment** | **Response** | **Changes Made** |
| --- | --- | --- |
| **Journal Requirements** |  |  |
| Please review your reference list to ensure that it is complete and correct. If you have cited papers that have been retracted, please include the rationale for doing so in the manuscript text, or remove these references and replace them with relevant current references. Any changes to the reference list should be mentioned in the rebuttal letter that accompanies your revised manuscript. If you need to cite a retracted article, indicate the article’s retracted status in the References list and also include a citation and full reference for the retraction notice. | We have reviewed the reference list to ensure that it is complete and correct. |  |
| **Reviewer 2** |  |  |
| The manuscript technically reads better after addressing reviewers' comments. However, proofreading is needed to enhance its quality and clarity (rephrasing, syntax, etc). Spotted ambiguous sentences and mistakes are highlighted in the attached file that include my comments. Specific attention is required to the strengths and limitations section; in addition to syntax issues, the discussion related to generalizibility and positive results within the scoping review approach requires reconsideration. | Thank you for your editing suggestions, which we have summarized below and implemented throughout the manuscript.  Regarding syntax errors, we note that Reviewer 2 exported the pdf proof of the manuscript to a Microsoft word document to allow editing with tracked changes. Unfortunately, this conversion introduced the highlighted syntax errors (missing quotations, dashes, apostrophes, etc.) throughout the manuscript. As those syntax errors are not present in the uploaded manuscript, no related changes were required.  We address your comments about generalizability and positive results below. |  |
| Line 28: suggested changing “objective” to "aim". | The required header from the journal is “Background and Objectives”. For clarity and consistency, we have removed any references to “aims” and now use “objective” throughtout the manuscript. | Lines 26, 29, 97, 98, 100. |
| Line 29: “the characteristics or type”. | Suggested change implemented. | Line 29: We have revised to “the characteristics and type” |
| Line 31: “the methods and steps”. | Suggested change has been implemented. | Line 32: We have revised to “guidelines”. |
| Lines 33 & 35: Number eligibility criteria. | Suggested change has been implemented. | Lines 33-35: We now indicate that “Included studies were primary research studies that 1) described an educational intervention for healthcare professionals as part of an initiative to promote practice change and 2) assessed functional outcomes (physical and/or cognitive).” |
| Line 38: “environment”  Reviewer 2: Ambiguous, similar to leadership/facilitation. You might say: other interventions addressed, care processes, environment, administrative support, facilitation/leadership, and discharge/transition planning | We have revised the category names to “leadership support/facilitation” “changes to the built environment” “administration/management support”. | Lines 38-42: We now indicate that “In addition to educational strategies, other interventions addressed care processes, changes to the built environment, administration/management support, facilitation/leadership, and discharge/transition planning. |
| Line 40: “functional outcomes in hospitalized older adults” | Suggested change implemented. | Line 42: We have revised to “functional outcomes among hospitalized older adults” |
| Line 54: “and loss of ability” | Suggested change implemented. | Line 56: We have revised to “and the loss of ability” |
| Line 68: “This scoping review sought to examine? interventions to support implementation”.  Reviewer 2: Characteristics and types? Or use “map” or “synthesize”. | Suggested change implemented. | Line 70: We now indicate that this “This scoping review sought to synthesize” |
| Line 91: “healthcare professionals’ perceptions of the feasibility of implementing”. | Suggested change implemented. | Line 93: We have revised to “healthcare professionals’ perceptions around the feasibility of implementing” |
| Lines 95-98: “In line with a research priority identified in a provincial priority-setting partnership, our aim was to determine what educational interventions for hospital-based healthcare professionals and/or interprofessional teams, have been implemented to optimize older adults functional outcomes in hospital settings.”  Reviewer 2: It was hard to understand this long sentence. You might consider rephrasing it. | Suggested change implemented. | Line 98: We have revised to “Our objective was to determine what educational interventions for hospital-based healthcare professionals and/or interprofessional teams, have been implemented to optimize older adults’ functional outcomes in hospital settings. The objective was identified as a research priority in a provincial priority-setting partnership.” |
| Line 108: “Using this approach,” | Suggested change implemented. | Line 111: We have revised to “Guided by this approach,” |
| Reviewer 2: number Inclusion/Exclusion criteria in lines 118-121. | Suggested change implemented. | Lines 121-124: We have revised to “Eligible studies 1) described educational interventions for healthcare professionals as a part of an initiative to promote practice change (e.g., care process change, environmental modifications), 2) assessed functional outcomes in older adults (physical or cognitive), and 3) addressed single or multiple interventions.” |
| Line 121: “and could address single or multiple interventions | Suggested change implemented. | Line 124: We have revised to “and 3) addressed single or multiple interventions” |
| Line 125: “An academic health sciences librarian (MK)…”  Reviewer 2: I don’t think initials are needed if MK is not among authors. | We have removed the initials of the academic librarian. | Line 128: We have revised to “An academic health sciences librarian…” |
| Line 152: “Research assistants (NM and SS) conducted data extraction”  Reviewer 2: see previous comment about inititlas. | Both NM and SS are co-authors. |  |
| Lines 165-166: “We did not re-frame the study findings beyond what was reported in the original studies.”  Reviewer 2: unclear | We have clarified this phrasing. | Lines 168-169: We have revised to “We developed preliminary categories, which were iteratively refined as analysis progressed, to provide a descriptive summary of the findings reported in the studies.” |
| Lines 166-167: “Given that this was a scoping review which should not address more targeted questions of intervention feasibility or effectiveness…”  Reviewer 2: need rephrase. | Suggested change implemented. | Line 169: We have revised to “Given that scoping reviews should not address targeted questions of intervention feasibility or effectiveness,^33^ we used our descriptive summary to map…” |
| Lines 201-202: “promote practice change: level of intervention, care process changes, environmental changes administration/management, leadership or facilitation roles, and discharge/transition planning”  Reviewer 2: Suggest: changes to the built environment. **"environmental changes,"** may be too broad, as it can also refer to ecological or climate-related changes. | Suggested change implemented. | Lines 203-204: We have revised to “promote practice change: intervention level, care process changes, changes to the built environment, administration/management support, leadership support/facilitation, and discharge/transition planning.” |
| Line 205: “Level of Intervention” header | Suggested change implemented. | Line 207: We have revised to “Intervention level” |
| Line 228: “Environmental Changes” header | Suggested change implemented. | Line 230: We have revised to “changes to the build environment” |
| Line 232: “Administration/Management” | Suggested change implemented. | Line 234: We have revised to “Administration/Management Support” |
| Line 235: “Leadership/Facilitation” | Suggested change implemented. | Line 238: We have revised to “Leadership Support/Facilitation” |
| Line 244: “Outcomes measured” | Suggested change implemented. | Line 247: We have revised to “Measured Outcomes” |
| Lines 259-261: The educational interventions that supported functional outcomes for hospitalized older adults in this review focused????????? on changes  ATTENTION TO THE WORDING THAT INDUCE MEANING CHANGE. Is it focused or were supported by/coupled with?  This sentence from the previous version was clearer: “In addition to education, a variety of interventions supported implementation of knowledge from the educational initiatives to improve functional outcomes for hospitalized older adults.” | We have reverted to the sentence from the previous version and updated the category naming. | Lines 262-267: “In addition to education, a variety of interventions supported implementation of knowledge from the educational initiatives to improve functional outcomes for hospitalized older adults: changes to care processes (e.g. multifocal protocols, mobility interventions, and cognitive interventions), changes to the built environment (e.g. walking trails, clocks, decluttering), administration/management support (e.g. purchase of equipment, electronic record modification), leadership support/facilitation (e.g. geriatricians and specialized nurses), and discharge/transition planning.” |
| Line 279: “The studies reported that educational interventions to enhance knowledge uptake, had a measurable effect on functional outcomes, but were overwhelmingly physical and/or cognitive, with very few focusing on cognitive function exclusively.”  Reviewer 2: Refer to studies or outcomes? rephrase needed/  …had a measurable effect on functional outcomes, THAT were overwhelmingly | Suggested change implemented.  Suggested change implemented. | Line 282-284: We have revised to “The studies reported that educational interventions to enhance knowledge uptake had a measurable effect on functional outcomes. However, the outcomes measured were overwhelmingly physical and/or cognitive, with very few studies focusing on cognitive function exclusively.”  Line 283: We have revised to “on functional outcomes that were overwhelmingly…” |
| Line 305: “Other gaps identified” | Suggested change implemented. | We have revised to “other identified gaps” |
| Lines 307-311: “While we are conscious that randomized controlled trials (RCTs), typically viewed as the gold standard in clinical research, are not always the most appropriate method for every research question and can be undermined by high costs, ethical considerations, and insufficient generalizability to broader, more diverse populations, we believe that more rigorous studies on the topic are needed.”  Reviewer 2: Long | Suggested change implemented. | Lines 311-314: We have revised to “We are conscious that randomized controlled trials (RCTs) – typically viewed as the gold standard in clinical research – are not always the most appropriate method for every research question. RCTs can be undermined by high costs, ethical considerations, and insufficient generalizability to broader, more diverse populations. We therefore believe that more rigorous studies on the topic are needed.” |
| Line 314: “to understand the education and other interventions provided to support implementation.” | Suggested change implemented. | Lines 316-318: We have revised to “understand the education intervention and other initiatives provided to support implementation” |
| Lines 321-322: “There was little evidence of administrative changes to support interventions; a few studies describing restraint policies or practices” | Suggested change implemented. | Lines 324-325: We have revised to “There was little evidence on administrative changes to support interventions; a few studies describing restraint policies or practices were identified” |
| Lines 326-328: “This study adds to the literature on care for hospitalized older adults by synthesizing the evidence on education that supported intervention implementation, scaling/spread, and sustainability, and by providing a close examination of other interventions supporting the implementation of gerontological knowledge, which are typically overlooked in reviews.”  Reviewer 2: unclear-rephrase needed. First sentence is long. | Suggested change implemented. | Lines 329-332: We have revised to “This study adds to the literature on care for hospitalized older adults by synthesizing the evidence on education that supported intervention implementation, scaling/spread, and sustainability. The study also provides a close examination of other interventions supporting the implementation of gerontological knowledge, which are typically overlooked in reviews.” |
| Line 330: We also grounded our study in a robust, context-sensitive theoretical conceptualization drawn from ecological implementation science frameworks.”  Reviewer 2: it is still unclear for me how this theory was used or linked to the scoping review? Did it guide the analysis, the reporting of results, or else? Either explain or remove. | Consequently, our ecological lens (which differentiates between micro, meso, and macro levels) guided both our analysis in terms of the level of healthcare practice change, and our reporting on that aspect of the studies in the text and in our S-1 Table. |  |
| Line 334: “…excluded in a review with language filters.” | We have not implemented this suggested edit as we feel it is important to specify explicitly that we conducted the review in English only. |  |
| Lines 335-336: “…education may have occurred in some studies that was not reported on (and therefore mistakenly excluded when assessing eligibility).  Reviewer 2: unclear. | Suggested change implemented. | Lines 338-340: We have revised to “…education may have been delivered to healthcare providers in some studies that was not reported on (and therefore those studies were mistakenly excluded when assessing eligibility). |
| Line 338: “…and did not include details of cognitive interventions or other environmental interventions in our search terms (though we did extract any cognitive outcomes reported).” | Suggested change implemented. | Lines 341-342: We have revised to “and did not include cognitive interventions or other environmental interventions in our search terms (though we did extract any cognitive outcomes reported). |
| Lines 339-340: “Future reviews should explicitly include cognitive function.” | Suggested change implemented. | Line 343: We have revised to “Future reviews should explicitly address cognitive function. |
| Line 342: “and we thus excluded studies with younger definitions of older adult…” | Suggested change implemented. | Lines 345-346: We have revised to “and we thus excluded studies which defined older adults as 64 years of age or younger.” |
| Lines 343-344: “Finally, as with all scoping reviews, there is an unknown risk of publication bias due to the tendency of researchers to publish only positive findings.”  Reviewer 2: what do you mean by positive. Is there in scoping reviews positive and non-positive findings? | We now explain that we mean that research studies with statistically significant, positive results tend to be published more than those with statistically insignificant or negative results. | Lines 346-347: We have revised to “As with all scoping reviews, there is also an unknown risk of publication bias due to the tendency of researchers to publish positive, statistically significant findings, and to not publish statistically insignificant or negative ones.” |
| Lines 344-346: “Finally, most studies were conducted in high-income countries and may not be generalizable to low- and middle-income countries.”  Reviewer 2: ScR are not concerned with generalizability. They map and summarize the available literature and provide descriptions on what is available as per my understanding | Suggested change implemented. | Lines 348-349: We have revised to “Finally, most studies were conducted in high-income countries and their findings may not be applicable to low- and middle-income countries. |
| Lines 348-349: “We conceive of this scoping review, which mapped the breadth of the existing evidence, as a precursor to a future systematic review”  Reviewer 2: Better to re-phrase | Suggested change implemented. | Lines 351-352: We have revised to “This scoping review, which mapped the breadth of the existing evidence, lays the foundation for future systematic reviews…” |
| Lines 349-350: “Future systematic reviews should build on our work by examining which of the interventions that we”  Reviewer 2: Rephrase suggested: for example, Building on this study, future systematic reviews can identify … | Suggested change implemented. | Lines 352-353: We have revised to “Building on this study, future systematic reviews should identify which…” |
| Lines 366-369: “Acknowledgements”  Reviewer 2: Inappropriate title: data availability? | Suggested change implemented. | Line 366: We have revised the header to “Data availability”. |
