# Supplementary material for: Interventions supporting the translation of gerontological evidence into practice to optimize functional outcomes for hospitalized older adults: A scoping review
Source: PLoS One. 2025 Jun 16;20(6):e0324953. doi: 10.1371/journal.pone.0324953 (PMC12169582; doi:10.1371/journal.pone.0324953)
Supplement: Attachment — Submitted filename: PLos_One_Scoping Review_Response to Reviewers_15_04_2025.docx [file pone.0324953.s011.docx]

**Response to Reviewer Comments Received April 15, 2025**

**Manuscript ID:** PONE-D-24-35120

**Title:** Interventions Supporting the Translation of Gerontological Evidence into Practice to Optimize Functional Outcomes for Hospitalized Older Adults: A Scoping Review

| **Comment** | **Response** | **Changes Made** |
| --- | --- | --- |
| **Reviewer 2** |  |  |
| Below, I draw your attention to minor required editing (in the track change version):  - L229: built instead of  build  - L309: "identified  gaps"  instead of "gaps  identified" - L340: were instead of  was | Thank you for catching these editing errors. These suggestions have been implemented in the manuscript. | PP 11, 15, & 16. |
